# A Review of In-Situ Techniques for Probing Active Sites and Mechanisms of Electrocatalytic Oxygen Reduction Reactions

**DOI:** 10.1007/s40820-022-00984-5

**Published:** 2022-12-29

**Authors:** Jinyu Zhao, Jie Lian, Zhenxin Zhao, Xiaomin Wang, Jiujun Zhang

**Affiliations:** 1 College of Materials Science and Engineering, Taiyuan University of Technology, Taiyuan, 030024 People’s Republic of China; 2 Institute for Sustainable Energy/College of Sciences, Shanghai University, Shanghai, 200444 People’s Republic of China

**Keywords:** Oxygen reduction reaction, Catalysts, In-situ techniques, Active sites, Mechanisms

## Abstract

Electrocatalytic oxygen reduction reaction (ORR) is one of the most important reactions in electrochemical energy technologies such as fuel cells and metal–O_2_/air batteries, etc. However, the essential catalysts to overcome its slow reaction kinetic always undergo a complex dynamic evolution in the actual catalytic process, and the concomitant intermediates and catalytic products also occur continuous conversion and reconstruction. This makes them difficult to be accurately captured, making the identification of ORR active sites and the elucidation of ORR mechanisms difficult. Thus, it is necessary to use extensive in-situ characterization techniques to proceed the real-time monitoring of the catalyst structure and the evolution state of intermediates and products during ORR. This work reviews the major advances in the use of various in-situ techniques to characterize the catalytic processes of various catalysts. Specifically, the catalyst structure evolutions revealed directly by in-situ techniques are systematically summarized, such as phase, valence, electronic transfer, coordination, and spin states varies. In-situ revelation of intermediate adsorption/desorption behavior, and the real-time monitoring of the product nucleation, growth, and reconstruction evolution are equally emphasized in the discussion. Other interference factors, as well as in-situ signal assignment with the aid of theoretical calculations, are also covered. Finally, some major challenges and prospects of in-situ techniques for future catalysts research in the ORR process are proposed.

## Highlights

- A comprehensive discussion of monitoring the structure evolutions of catalysts during ORR via multiple in-situ techniques to identify the active sites is presented.
- The extensive applications of in-situ techniques in elucidating the oxygen reduction reaction (ORR) mechanisms/pathways are reviewed.
- The challenges and recommendations of current in-situ techniques for monitoring the various dynamic evolutions in ORR are pointed out.

## Graphical abstract

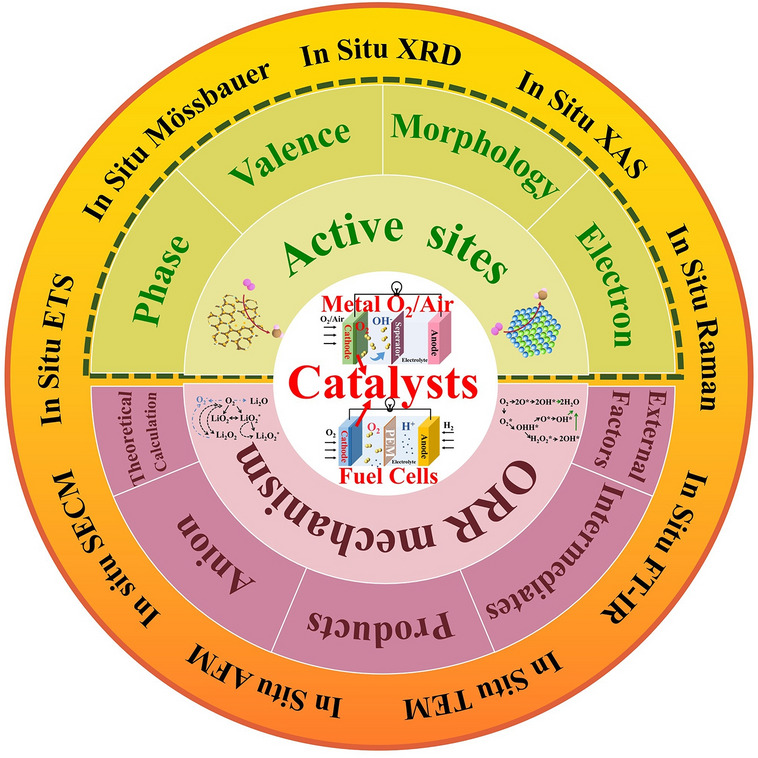

## Introduction

Exploration of clean and sustainable energy sources, such as wind, solar, and hydroelectric power and the development of their associated electricity energy storage and conversion technologies are inevitable to undertake a secure and sustainable prospect [1]. For electricity storage and conversion technologies, electrochemical energy technologies including water electrolysis to produce hydrogen, fuel cells to convert hydrogen to electricity, metal–O_2_/air batteries to store/convert energy, etc., have been awaked as reliable, safe, efficient, environmental, and sustainable selections [2–4]. For fuel cells, such as proton exchange membrane fuel cells (PEMFCs), direct borohydride fuel cells (DBFCs), and metal–O_2_/air batteries such as lithium–O_2_/air (Li–O_2_/Air) battery, sodium–O_2_/air (Na–O_2_/Air) battery, as well as zinc–O_2_/air (Zn–O_2_/Air) battery, the oxygen reduction reaction (ORR) of their cathodes has been distinguished as the reaction dominating the performance of these devices owing to its slow reaction kinetics [5–7]. Therefore, some active and stable electrocatalysts coated on the cathodes are necessary to catalyze the ORR to a practical rate.

Currently, the most practical ORR electrocatalysts are platinum (Pt)-based catalysts [8, 9], and their multiple designs for superior ORR activity have been reported extensively. In detail, Pt nano-catalysts with multi-dimensional morphologies [10–12], such as nanocages (NC) [13], nano-frames [14], nanosheets [15], nanowires (NW) [16], nanotubes (NT) [17], and nano-dendrites (ND) [18], have been developed. Each catalyst either possesses open, hollow, porous nanostructures, or high-energy surfaces, which can either contribute to increase the exposure density of Pt active sites, facilitate electron transport/mass exchange, or help to enhance Pt atom utilization, eliminate Pt dissolution and aggregation, thereby improving the ORR activity and stability [19–21]. Also, a great deal of research has focused on the introduction of economic transition metals (M = Fe, Co, Ni, Cu, etc.) into Pt to form Pt-based alloys (Pt–M) catalysts with high ORR performance. As identified [22, 23], the synergistic effect of geometry, ligands, and optimized electronic structure in the Pt-M catalyst can reduce Pt consumption, enhance Pt intrinsic activity, and reduce other side reactions, thereby strengthening its ORR activity and durability. Furthermore, owing to resource shortage and the high price of Pt-based materials, many low-cost non-platinum catalysts have been explored [24–26], containing heme centered mimetic macromolecules (e.g., metal-phthalocyanines or -porphyrins) [27–29], metal oxides [30–33], and transition metals based catalysts [34, 35] etc. Subsequently, nitrogen-functionalized graphene catalysts are widely proven to be metal-free catalysts with high ORR performance [36–38]. Thereafter, the field of metal-free catalysts has undergone rapid development and miscellaneous carbon-based catalysts with heteroatom doping have been reported [39]. Expressly, transition metal (i.e., M = Fe, Co, Ni, Cu, and Mn, etc.) and nitrogen dual doped carbon catalysts (i.e., M–N–Cs, containing relevant single-atom catalysts (SACs)) [40–42] have stood out among these candidates, owing to their beginning-of-life ORR activities approaching those of Pt-based catalysts in both acidic and alkaline electrolyte liquors [43, 44].

Considering that the superior performance is markedly dependent on the active site of the catalyst [45, 46], it is imperative to clarify the active ingredient and monitor the correlative evolution. Over the past decades, various usual ex-situ characterization techniques are applied to describe the phase, valence, electron transfer, coordination, and spin state of the catalyst before and after the electrocatalytic reaction to identify the active site and infer its variations. However, since the catalyst is prone to irreversible changes when exposed to air during transfer in ex-situ tests, the conclusions drawn may not match the reality and may cause misinterpretation [47]. More importantly, the catalyst always undergoes some dynamic structural evolution under practical conditions, leading to the evolution of active site, also increasing the difficulty of identification. Thus, the development of in-situ characterization techniques to monitor the catalyst structure evolution in real time is essential to determine the active sites, which can also reveal the rationality of the catalyst structure design and guide the synthesis of the highly active catalyst. In addition, despite a rational design of the catalyst with the highly active sites being crucial for efficient ORR, the reactions that occur during synthesis are not known, leading to a complex synthesis process, and exacerbating costs [48, 49]. Thus, the visual monitoring of the structural morphological changes, including nucleation, growth, reconstruction, Ostwald ripening, etc., for the catalyst during the synthesis process is also essential to simplify the synthesis process and shorten the synthesis time, which cannot be ignored.

In view of the ORR pathways are multiple, during which there are abundant and complex intermediates/products, and they are also related to the active site of the catalyst and are vital for the revelation of the ORR mechanism. However, extensive works have confirmed that they will also occur in a series of conversions and reconstructions under experiment conditions, resulting in them being hard to be distinguished accurately [50, 51], and their transient evolutions are difficult to capture due to the fast rate of the catalytic reaction [52–54]. As well as, the postprocess nature of common ex-situ characterization techniques limit the recognition of their true changes in real time. Hence, the exploitation of in-situ characterization techniques to accurately determine the evolutionary behavior of the intermediates (adsorption/desorption) or products (such as nucleation, growth, and reconstruction) is very necessary and should be equally emphasized [48]. Expressly, in-situ techniques can reveal their dynamic changes during the reaction and do not require the dismantling of the test cell, thus obtaining more valuable information to reversely clarify the active site and intuitively elucidate the ORR mechanism [55–57]. Notably, the electrolyte anions have competitive adsorption features with intermediates on the catalyst surface, which can cause catalyst surface poisoning and thus affect the reaction pathway. Thus, the chemisorption of electrolyte anions on the catalyst surface is a non-negligible object to be detected during ORR, which can provide information on the dynamic evolution of the catalyst surface closer to the experimental conditions. Furthermore, to ensure accurate identification of active site and precise elucidation of ORR mechanism, a great deal of works have been devoted to combining theoretical calculations to assist in facilitating the assignment of detected signals [58, 59]. Of course, the proper in-situ cell design is a guarantee for efficient in-situ detection and has also received a lot of attention [45, 60]. And each technique has its own unique detection characteristics and limitations; therefore, the combination of techniques to provide more adequate and useful information has also been the focus of researchers.

**Fig. 1:**
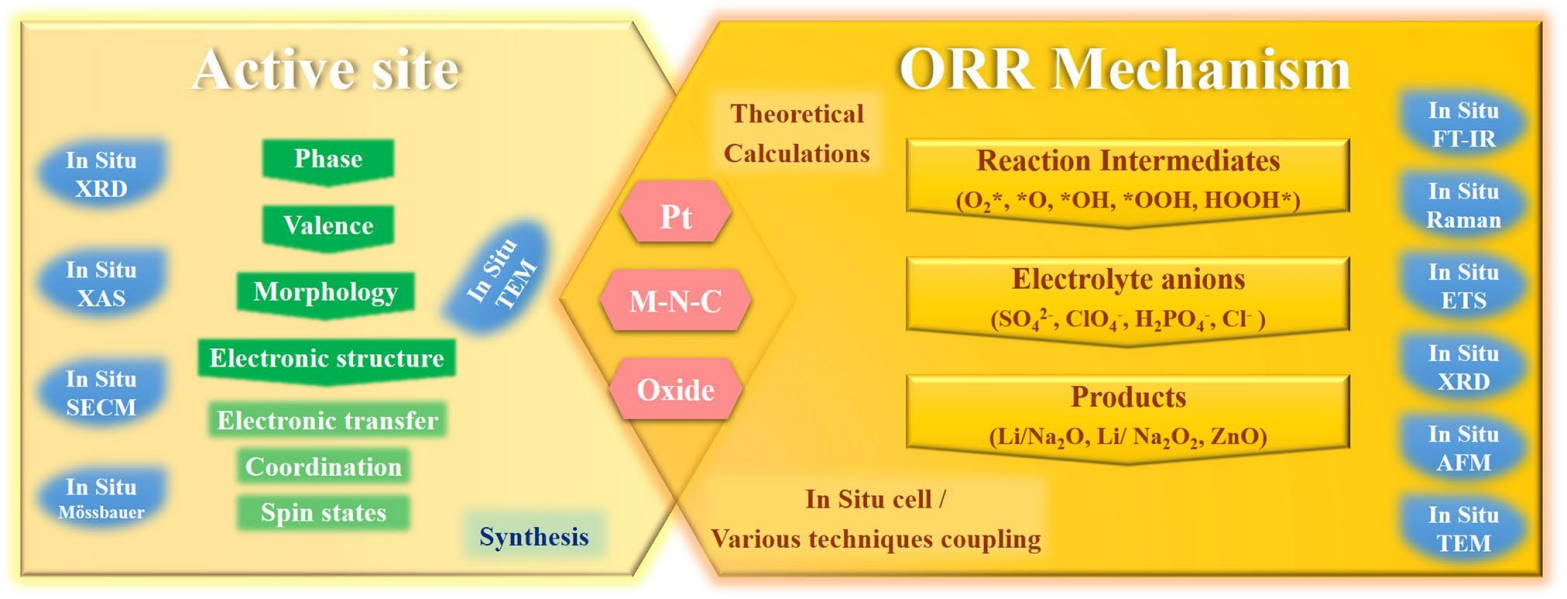
Schematic diagram of the main overview content

This work reviews the main advances in characterizing the catalytic processes of different catalysts in fuel cells and metal–O_2_/air batteries by using various in-situ techniques, and with a focus on Pt-based, M–N–C and some oxide catalysts (Fig. 1). In detail, the discuss starts with the accepted ORR mechanism form the representative batteries, and listing possible intermediates and products involved in the reaction. The working principles of various in-situ techniques and their unique detection for ORR are briefly outlined. The direct in-situ characterizations of catalyst structure evolution during ORR are systematically summarized, while, in part, covering a discussion of its morphological monitoring in the synthesis process. Then, the focus is on outlining the dynamic evolution information of intermediates and products, and providing some chemisorption information of solvent anions. These two sections reveal the factors affecting the catalytic performance of catalysts from direct or indirect perspectives and thus guide the direction of optimization of their structural design. More importantly, these provide important guidance to clarify the active center and elucidate the ORR mechanism. In addition, the integration of theoretical calculations is further emphasized to promote in-situ signals assignment. The design of in-situ cells, and the coupling of several techniques are also covered to ensure accurate information acquisition. Finally, based on achievements and challenges of present in-situ techniques, some future research directions are proposed for overcoming challenges for a better understanding of the ORR mechanism.

## Brief Overview of the ORR Mechanism and Intermediates

### Mechanism of ORR in Fuel Cells and Intermediates

**Fig. 2:**
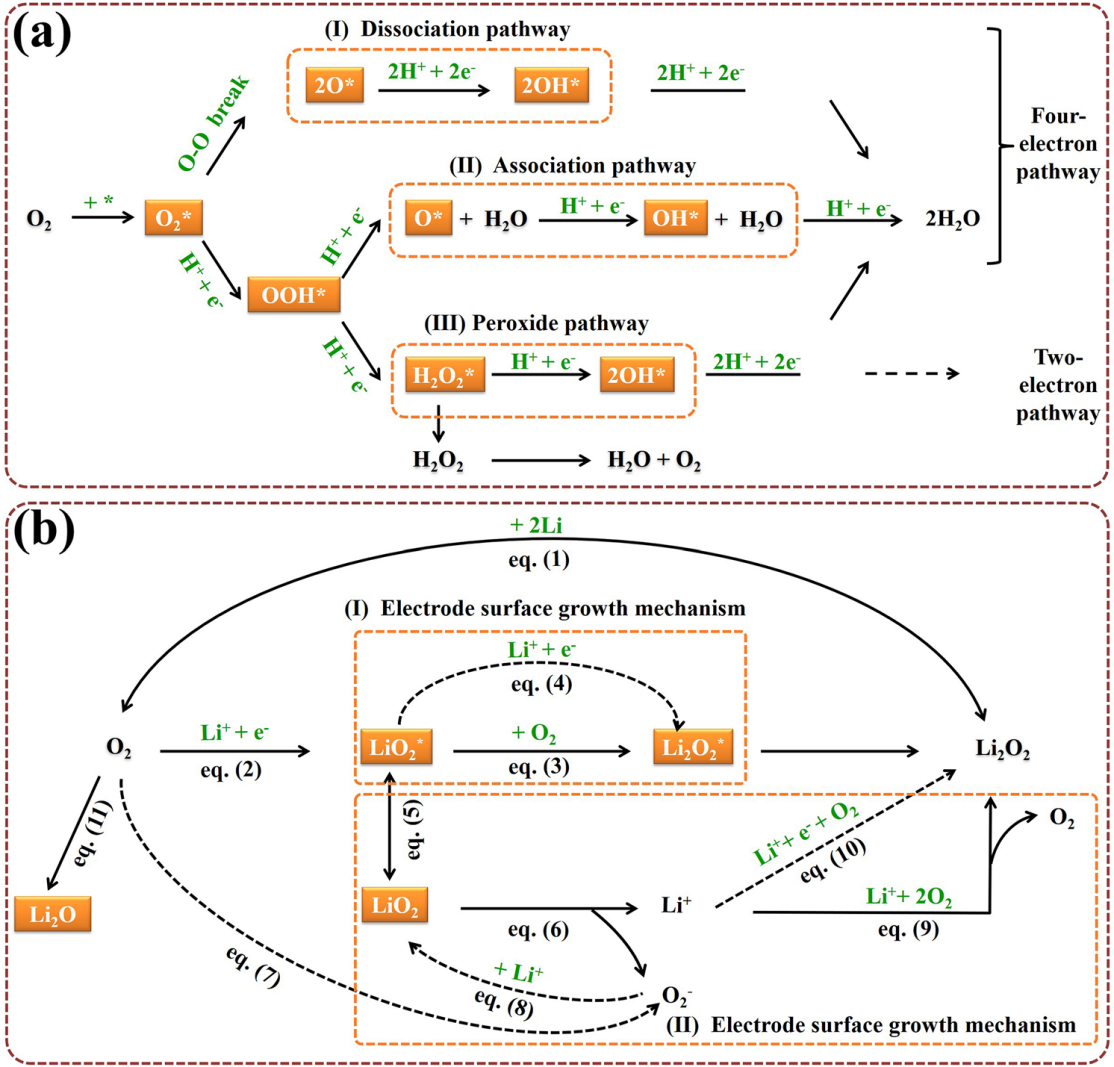
**a** ORR mechanism of PEMFC (one kind of fuel cell), and **b** Li_2_O_2_ battery (one kind of metal–O_2_/air battery), both with reaction pathways in the orange dash frames, with intermediates in orange solid frames

For different fuel cells, the difference is the anode reaction, the cathode reaction is the same, namely ORR. Taking PEMFC (typical one) as an example, its ORR occurs at the cathode, where O_2_ get electrons to produce a series of oxygen-containing species [61, 62]. Specifically, the oxygen molecules first diffuse to the catalysts surface to form adsorbed oxygen molecules (O_2_*, where * denotes the active site) (Fig. 2a). Then the adsorbed oxygen molecules, O_2_*, undergo a reduction reaction, which can be divided into three pathways based on the breakage order of the O–O bonds. The first pathway is the dissociation pathway, where the O–O bonds directly break to form O* intermediates, followed by the successive reductions to OH* and H_2_O*. The second pathway is the association pathway, where O_2_* first forms OOH*, and then the O–O bonds are cleaved to produce O* and OH* intermediates. The third pathway is the peroxide pathway, where O_2_* intermediates are reduced sequentially to OOH* and HOOH* before the breaking of O–O bonds. This means monitoring the catalytic process and capturing intermediates information are beneficial for elucidating the ORR mechanism.

Notably, the peroxide pathway is the two-electron pathway, which only needs to overcome an energy barrier of 146 kJ mol^−1^ [23, 63]. However, the peroxide intermediates are difficult to be completely reduced, and some may be reversibly decomposed to form oxygen molecules, resulting in low current efficiency [64, 65]. Also, these intermediates can oxidize/corrode the catalytic active center and carbon carrier, thus significantly weakening the activity and stability of the catalyst. In contrast, the former two pathways both are the four-electron pathways, although with a higher energy barrier to overcome, but can efficiently convert oxygen molecules to H_2_O without producing oxidizing/corrosive intermediates. Thus, it is essential to monitor the intermediate changes during the reaction and further clarify the catalytic pathway, which can help guide the design of highly active and stable catalyst (i.e., following a four-electron pathway not a two-electron pathway).

Moreover, the adsorption/desorption process of oxygen-containing intermediates (e.g., O_2_*, *O, *OH, *OOH, and HOOH*) on the catalyst surface is the key to affecting the ORR kinetics [66, 67]. The adsorption process of O_2_ (i.e., O_2_ → O_2_*), as the initial step, determines the course of the subsequent reaction steps. The adsorption/desorption of OH occurs at a high potential, which determines the initial potential at which the reaction begins to proceed. Also, the amount of OH adsorbed determines the number of available active centers, while the ability of adsorbed OH to bind with proton/electron further limits the reaction rate. More importantly, the adsorption energy of intermediates on the given catalyst surface is positively correlated with the ORR catalytic activity [68–70]. It is often considered as a catalytic activity descriptor, which can be obtained by the theoretical calculation. If the adsorption strength is too weak, it will limit the proton/electron transfer; conversely, too strong adsorption will cause desorption and become difficult. Therefore, it is necessary to combine theoretical calculations to guide the design of catalysts with moderate adsorption energy.

### Mechanism of ORR in Metal–O_2_/Air Batteries and Intermediates

For a series of metal–O_2_/air batteries, the ORR is responsible for their discharge processes and undergoes similar reaction mechanism. Taking a typical Li–O_2_ battery as an example (Fig. 2b), the dominant reaction equation is as follows:

1 $${\text{2Li}} + {\text{O}}_{{2}} \leftrightarrow {\text{Li}}_{{2}} {\text{O}}_{{2}} \left( {\text{s}} \right),\;\;\left( {E_{0} = {2}.{96}\;{\text{V}},\;{\text{vs}}.\;{\text{Li}}/{\text{Li}}^{ + } } \right)$$

The forward direction is the discharge reaction, during which different kinds of intermediates (i.e., lithium oxides/mixtures) and ideal Li_2_O_2_ products can be produced by reduction reaction of O_2_ [71–73]. Concretely, the first step is the O_2_ combines one electron, resulting in superoxide ion (O_2_^−^), and followed by meeting the Li^+^ to form LiO_2_. Then, LiO_2_, as the intermediator, is thermodynamically unstable and can rapidly convert to Li_2_O_2_. Based on the degree of solvent solubilization of the cations produced by LiO_2_, the growth process of Li_2_O_2_ can be divided into two categories [74–77].

Firstly, for electrode surface growth mechanism, in the weak Li^+^ solubilized solution, LiO_2_ will be adsorbed on catalysts surface of the electrode, forming LiO_2_*,

2 $${\text{Li}}^{ + } + e^{ - } + {\text{ O}}_{{{2}({\text{sol}})}} \to {\text{ LiO}}_{{2}}^{*}$$

Then, LiO_2_* undergoes disproportionation to form Li_2_O_2_* and O_2_; or it receives a single *e*^*−*^ and Li^+^ from the electrode and electrolyte, respectively, and continues to be reduced to Li_2_O_2_^*^,

3 $${\text{2LiO}}_{{2}}^{*} \to {\text{ Li}}_{{2}} {\text{O}}_{{2}}^{*} + {\text{ O}}_{{2}}$$

4 $$\left( {{\text{or}}} \right){\text{ LiO}}_{{2}}^{*} + {\text{ Li}}^{ + } + e^{ - } \to {\text{ Li}}_{{2}} {\text{O}}_{{2}}^{*}$$

Secondly, for solution growth mechanism, in a strong Li^+^ solubilized solution, adsorbed LiO_2_* breaks away from the electrode surface to form soluble LiO_2(sol)_ and dissolves into electrolyte, followed forming Li^+^_(sol)_ and ${\text{O}}_{{2\left( {{\text{sol}}} \right)}}^{ - }$,

5 $${\text{LiO}}_{{2}}^{*} \leftrightarrow {\text{ LiO}}_{{{2}\left( {{\text{sol}}} \right)}}$$

6 $${\text{LiO}}_{{{2}\left( {{\text{sol}}} \right)}} \to {\text{ Li}}^{ + }_{{\left( {{\text{sol}}} \right)}} + {\text{ O}}_{{2\left( {{\text{sol}}} \right)}}^{ - }$$

In addition, the LiO_2(sol)_ can also be grown from the reaction between soluble superoxide ion ${\text{O}}_{{2\left( {{\text{sol}}} \right)}}^{ - }$ and Li^+^,

7 $${\text{O}}_{{2}} + \, e^{ - } \to {\text{ O}}_{{2\left( {{\text{sol}}} \right)}}^{ - }$$

8 $${\text{O}}_{{2\left( {{\text{sol}}} \right)}}^{ - } + {\text{ Li}}^{ + } \to {\text{ LiO}}_{{{2}\left( {{\text{sol}}} \right)}}$$

Finally, the formed Li^+^_(sol)_ and O_2 (sol)_ in the electrolyte can either undergo disproportionation to form Li_2_O_2_ and O_2_, or again undergo a single *e*^*−*^ transfer electrochemical process to produce Li_2_O_2_,

9 $${2}\left( {{\text{Li}}^{ + }_{{({\text{sol}})}} + {\text{O}}_{{{2}({\text{sol}})}} } \right) \to {\text{ Li}}_{{2}} {\text{O}}_{{2}} + {\text{ O}}_{{2}}$$

10 $$\left( {{\text{or}}} \right){\text{2Li}}^{ + }_{{({\text{sol}})}} + e^{ - } + {\text{ O}}_{{{2}({\text{sol}})}} \to {\text{ Li}}_{{2}} {\text{O}}_{{2}}$$

This means adopting in-situ characterization techniques to monitor the intermediates behavior, such as LiO_2_^*^ et al., can help to reveal the ORR mechanism.

Furthermore, O_2_ can be reduced to Li_2_O by a four-electron transfer process, following,

11 $${\text{4Li}}^{ + } + {4}e^{ - } + {\text{ O}}_{{2}} \to {\text{ 2Li}}_{{2}} {\text{O}}$$

Notably, a discharge of Li_2_O can augment the theoretical specific energy, but the electrochemical irreversibility of it results in an even higher charge overpotential [78]. So that it is desired to design the catalyst to help to form the ideal Li_2_O_2_ product during ORR process.

## Succinct Overview of Various In-Situ Characterization Techniques

In this section, the operational principles, unique characteristics, and drawbacks of each characterization-technique have been summed up. An in-depth comprehension of characteristics for each technique will facilitate its targeted use in the ORR dynamic monitoring process. Some summaries of related in-situ cells are also included.

### In-Situ X-Ray Diffraction (XRD)

**Fig. 3:**
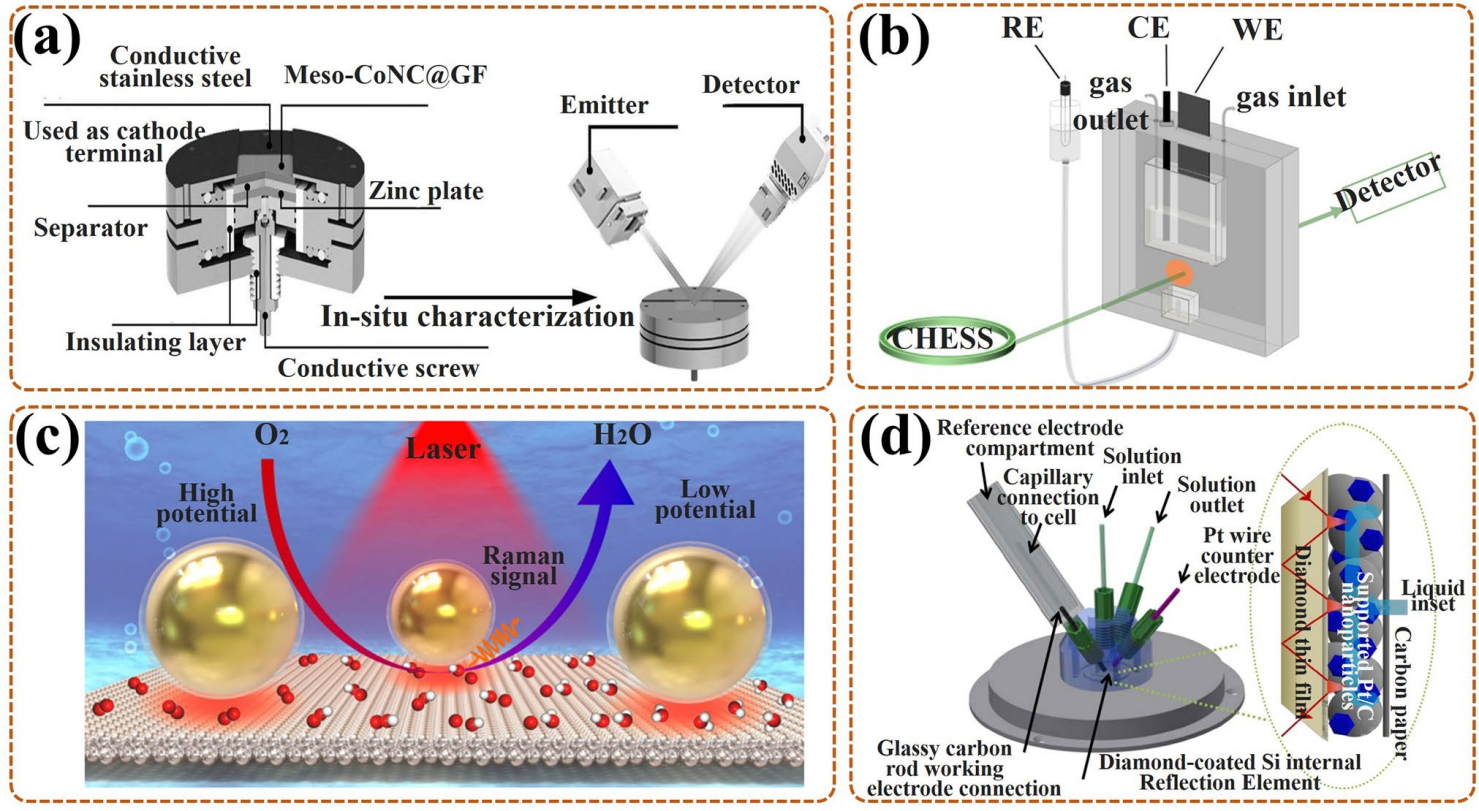
**a** Schematic diagram of in-situ XRD cell and detection principle for Meso-CoNC@GF air electrode during ORR in Zn–Air battery [81]; Reused with approval; Copyright 2017 WILEY-VCH Verlag GmbH & Co. KGaA, Weinheim. **b** Sketch map of in-situ XAS cell, with WE, i.e., carbon paper coated with catalysts and CE, i.e., carbon rod dipped in KOH solution (1 M) [84]; Reused with approval; Copyright 2018 American Chemical Society. **c** The model of Au@SiO_2_ NPs on low-index (111) Pt surface, and the ORR mechanism promulgated by in-situ SHINERS, with Au core (golden), SiO_2_ shell (transparent), Pt (silver-white), O (red), and H (white) [89]; Reused with approval; Copyright 2018 Springer Nature. **d** Schematic diagram of in-situ ATR-IR cell, with Pt/C nanoparticles as catalysts in HClO_4_ solution (0.1 M) [93]; Reused with approval; Copyright 2018 WILEY-VCH Verlag GmbH & Co. KGaA, Weinheim. (Color figure online)

XRD, as a form of elastic scattering, can provide unique diffraction patterns for periodically structured materials, revealing their precise crystallographic information [47, 79]. Concretely, the size, dimension, shape, and orientation of the crystal unit determine the location of the diffraction peak, and the type and location of the atoms determine the intensity of the diffraction peak [60, 80]. In-situ XRD can detect the crystalline phases during ORR in real time, and the time-varying diffraction patterns can exhibit phase transition information on catalysts and intermediates. For real-time detection, the special in-situ cell needs to be assembled, such as, a tailor-made cell with a radial X-ray penetration window for XRD analysis was designed by Liu et al. [81]. Also, the experimental conditions were integrated in their cell (Fig. 3a), including MesoCoNC@GF, Zn plate, Whatman glass microfiber filter, and KOH + zinc acetate, as the air cathode, anode, separator, and electrolyte. However, if the intermediates are very thin with very low crystallinity, the existing in-situ XRD cannot complete the detection, in addition, it is not possible to detect the local sites of the catalyst.

### In-Situ X-Ray Absorption Spectroscopy (XAS)

XAS (also name as X-ray absorption fine structure, XAFS), is a form of inelastic scattering, including X-ray absorption near-edge structure (XANES) (with a region up to ~ 25 eV above the edge), and extended X-ray absorption fine structure (EXAFS) (with a region of higher energies) [48, 79]. The former (XANES) can reveal the oxidation state and electronic configuration information. In detail, the target atom of a higher oxidation state, always with a higher effective charge of its nucleus, requires extra energy to excite its core electrons in comparison to other atoms [82]. The latter (EXAFS) is used to supply information on interatomic spacing or coordination numbers, which are attributed to the constructive or deconstructive interference caused by electron scattering, respectively [83]. Thus, in-situ XAS is regarded as a mighty tool for in-situ verification of electronic structure changes around the excited atom during ORR. As shown in Fig. 3b [84], an in-situ cell was made by Teflon material with chemical inert, in which the working electrode (WE) was constructed by spraying the catalyst layer (with thick of 40 μm) on carbon paper (200 μm). Also, it had an X-ray penetration window, and the electrolyte thickness was less than 200 μm, both ensuring highly quality data acquisition for in-situ XAS analysis. To reduce the IR drops induced by the thin electrolyte layer resistance, the reference electrode (RE) was linked to the cell via a salt bridge. Regrettably, it is unable to research low atomic number elements, despite it can in-situ characterize amorphous materials, also with a wider range of X-ray photon energies.

### In-Situ Raman Spectroscopy

Raman spectroscopy, also as inelastic scattering, can identify structural information of molecules with Raman active vibrational modes [85, 86]. To improve the Raman intensity of necessarily and randomly adsorbed intermediates in ORR, the development of surface-enhanced Raman spectroscopy (SERS) or surface enhanced resonance Raman scattering (SERRS) is essential, especially in-situ Raman analysis [87, 88]. In-situ shell-separated nanoparticle-enhanced Raman spectroscopy (SHINERS) is the most in-depth research, particularly, revealing for ORR mechanism of low-index Pt (hkl) surface on Au@SiO_2_ nano-particles (NPs) [89]. In detail, Au nanoparticles act as cores, for amplifying signals; thin silica with pinhole-free acts as shells, for separating the target molecules from the core, thereby eliminating the interrelated effects (Fig. 3c). Even in aqueous solutions (weak scatterers), the vibrational and rotational energy levels of intermediates on the Pt surface can be acquired in real time. Nevertheless, Raman spectroscopy is not detectable in certain catalytic devices, such as pure metal catalysts.

### In-Situ Fourier Transform Infrared (FT-IR) Spectroscopy

FT-IR spectroscopy, as an absorption spectrum, can check diversification in dipole moments triggered by rotations and vibrations of bonds in fragment molecules, radicals, and functional groups [90]. In-situ FT-IR allows real-time tracking of groups generated on the catalyst surface during ORR, with the frequencies or intensities in spectrum interrelated with category or homologous substance quantity, respectively [91]. Prominently, in-situ attenuated total reflection infrared (ATR-IR) spectroscopy with better spectral reproducibility focuses on measuring IR radiation variations after contacting with intermediates in real time, providing further insight into the ORR mechanism [92]. To avoid mass transport limitations during ORR, Nayak et al. [93] developed a modified cell, with a flow field of electrolyte around catalysts, to proceed in-situ ATR-IR testing, its work mechanism likes that of a rotating disk electrode (Fig. 3d). Regretfully, in the aqueous phase experiments, the IR light is sensitive to H_2_O molecules, making the signal of in-situ FT-IR exceedingly weak.

### In-Situ Transmission Electron Microscope (TEM)

**Fig. 4:**
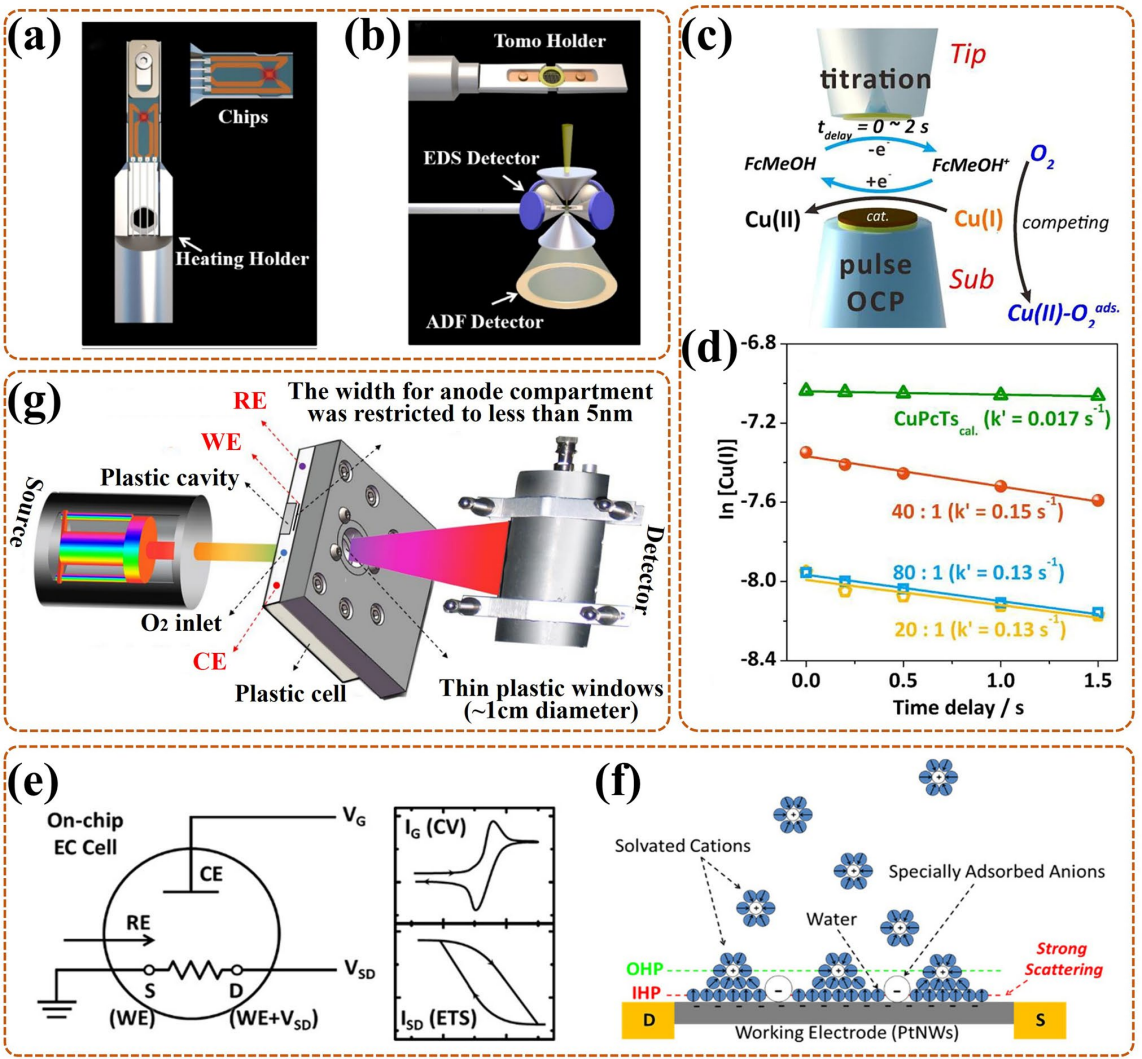
Elementary diagram of the in-situ TEM cell: **a** special heating holder and chip, **b** electron tomography, with HAADF and EDX detectors for collecting 2D HAADF and elemental allocation images at diverse orientations by rotating the holder [96]; Reused with approval; Copyright 2021 Elsevier Ltd. **c** Schematic diagram of in-situ SI-SECM cell and relevant titration mechanism (with OCP as open circuit potential); **d** The kinetic rate reflected by ln [Cu(*I*)]) against *t*_delay_; Reused with approval [101]; Copyright 2020 Elsevier Ltd. **e** The working principle diagram of ETS and CV at the same time in a three-electrode system (with CE, RE, WE) (S, source; D, drain); **f** The schematic diagram of in-situ ETS cell, with PtNWs electrode and aqueous electrolyte, with anions adsorbed on the IHP [103]; Reused with approval; Copyright 2018 American Chemical Society. **g** The illustration of in-situ ^57^Fe Mössbauer cell for in-situ testing Fe active site during ORR [109]; Reused with approval; Copyright 2020 Elsevier

TEM is another intuitive means to observe the nano-materials morphology [94]. Also, it can be applied in the authentication of the crystal structure to gain information on interplanar spacing, owing to its electron diffracted beams being related to the atomic structure of the material [45]. Thus, in-situ TEM can monitor morphological variations of catalysts during ORR in real time, and it can also realize the simultaneous dynamic monitoring of chemical reactions. Notably, in-situ aberration-corrected scanning/transmission electron microscopy (S/TEM) allows real-time characterization of material structures through quantum mechanical interactions induced by incident electron wave fields and atomic potentials [95]. There exist two imagery models, bright-field imagery, and high-angle annular dark-field (HAADF) imaging, particularly, the latter as the main one can in-situ exposit structure information from the perspective of atoms. Moreover, this in-situ test is always equipped with electron energy loss spectroscopy (EELS) and energy dispersive X-ray spectroscopy (EDX) to facilitate the provision of elemental, component, and electronic configuration information of the catalyst. More importantly, it is also possible to further introduce optical, electrical, and thermal signals into the cell to undergo this in-situ test. For example, Gong et al. [96] added a heat source into the cell to ensure in-situ observation of the ORR at variable temperature conditions (Fig. 4a); also, in-situ HAADF and elemental distributions were amassed from the relevant detectors by rotating the tomography holder under the scope of tilt angles (Fig. 4b). However, in-situ TEM places relatively stringent requirements on the material preparation and operating environment, especially for high-resolution measurements under the liquid solution.

### In-Situ Atomic Force Microscopy (AFM)

AFM, based on force, is a typical surface scanning technique, also known as scanning probe microscopy (SPM) [86]. For the main application in ORR of metal–O_2_/air battery, in-situ AFM, especially contact mode, can trace the morphological varies of the catalyst surface/interface in real time. Namely, it can in-situ scan the vertical morphology of the catalyst surface using a constant repulsive force between the probe and surface [90]. Notably, it is appropriate to a wide range of materials, independent of the electrical conductivity, and can work in a liquid solution due to there being no beam or lenses. Even though, to enhance the stable contact of probe and surface, it is crucial to ensure in-situ cell with an undisturbed liquid environment during ORR. That is, it is better to start in-situ AFM detection after the electrolyte saturating with O_2_ [97, 98]. The challenges for in-situ AFM are the contacting way of the cantilever beam with the electrode, and the inertness of the sample on the electrode.

### In-Situ Scanning Electrochemical Microscopy (SECM)

SECM, as another SPM technique, is based on current and can offer electrochemical imaging information for a wide range of catalyst surface/interface with insulating/conducting [90]. It does not require surface contact (unlike AFM) and consists of three operating modes: generation, acquisition, and feedback, with the last one being the most used. Recently, in-situ SECM is widely applied to elucidate the active site number and catalytic kinetic rate of the catalyst for ORR and it is conducted by connecting a workstation bipotentiostat. Typically, in-situ SECM cell (Fig. 4c) contains aligned tip (for generating titrant) and substrate (for distributing catalyst) ultramicroelectrodes (UMEs), also, the inter-electrode distance is always controlled in less than 5 μm [99, 100]. When the titrant detects the active intermediate formed on the catalyst surface quantitatively and sensibly at given potentials in real time, the active site number information can be offered. When a delay time (*t*_delay_) is adopted between tip and given potential on the substrate (i.e., time-delay titration), the residual active sites can be in-situ quantified, further the kinetic rate information can be obtained. In the work of Li et al. [101], the Cu(I) was easily oxidated by FcMeOH^+^ (tip-generated titrant) rather than O_2_, during Cu SAC catalytic ORR, supporting in-situ time-delay titration test, and more kinetic information derived from the log-integrated charge density (ln [Cu(I)]) against *t*_delay_ (Fig. 4d). However, the nanoscale resolution for SECM is challenging, being limited by the tip size and its distance to the substrate.

### In-Situ Electrical Transport Spectroscopy (ETS)

ETS is an electrochemical interface analysis technique, based on the fact that the chemical sensitivity of metal nano-catalysts can be converted to electrical signals [102]. For in-situ ETS, cyclic voltammetry (CV) and ETS are simultaneously measured in a three-electrode system in real time by a dual-channel source measure unit (SMU). More precisely (Fig. 4e), one supplies gate voltage (*V*_G_) for the working electrode (WE) and reference electrode (RE), while the Faradaic current (*I*_G_, in line with CV electrochemical current) is collected through counter electrode (CE). The other applies a petty bias voltage (*V*_SD_) for the source/drain electrode (i.e., WE), simultaneously, the electrical conduction current (*I*_SD_) is collected, namely, ETS signals. For ultrafine metallic nano-catalysts [103], the solution anions tend to adsorb on the inner Helmholtz plane (IHP, among solid and liquid interfaces), with difficult access for the radiation, making in-situ ETS essential for monitoring their surface adsorbed behavior. Taking platinum nanowire (PtNW) as an example, in-situ ETS provided a semaphore that was extremely susceptive to the dynamic evolution of surface states during ORR, thus revealing the effect of surface anions adsorption on ORR. In the cell (Fig. 4f), the PtNW was placed among source and drain electrodes with gold protection, also it only responded to the strong scattering effect of adsorbed anions, thus ensuring easy detection by the current. Nevertheless, for some catalysts only with weak chemical sensitivity, it is difficult for using in-situ ETS to track the related scattering effect signals on their surface.

### In-Situ Mössbauer Spectroscopy

Mössbauer spectroscopy, based on the Mössbauer effect, is a technique for detecting the nuclear state of certain specific elements (e.g., Fe, Sn, Ru, and Au). In-situ Mössbauer spectroscopy can track nuclei states of these elements in real time, including chemical state, coordination symmetry, spin state, and magnetic moment [104, 105]. Specifically, the isomer shift (IS) parameter, interrelated with the electronic structure, can ascertain the information about the chemical shifts. The quadrupole splitting (QS) parameter can reveal the electronic symmetry, further reflecting the spin state. The magnetic Zeeman splitting (B) parameter is invariably used to furnish the magnetic structure information [105, 106]. Recently, in-situ ^57^Fe Mössbauer spectroscopy has been widely used to reveal dynamic evolutions of spin state and chemical shift of Fe active site in Fe–N–C catalyst during ORR [107, 108]. Notably, to avoid excessive attenuation of γ-rays, the width for the electrode chamber of in-situ cell should be limited to a smaller size. For example, the compartment was restricted in a plastic cavity by Li et al., with a width of less than 5 mm (Fig. 4g) [109]. Unfortunately, only a limited number of nuclei have the Mössbauer effect, and the experimental conditions are harsh.

In summary, in-situ characterizations can ensure the real-time detection of catalytic process as much as possible, thereby deepening our understanding of the ORR mechanism. Also, these are very helpful to clarify relationship between the structure of the catalyst and ORR activity, further guiding the reasonable design of catalyst. Notably, each in-situ characterization technique exhibits its unique characteristic, as summarized in Table 1, and shares complementary advantages from each other. An appropriate in-situ technique should be selected based on its characteristics that match the requirements of the target catalyst. In the subsequent section, we will outline the various applications of in-situ techniques for monitoring dynamic evolution of the ORR process from multiplex perspectives.

**Table 1:** Unique characteristics, challenges, and improvement direction of different in-situ techniques

Techniques	Unique characteristics	Challenges and direction
In-situ XRD	Detect dynamic crystalline phase transitions	Both lack of highly accurate temporal resolution; XRD, tens of seconds; XAS, a few minutes; intermediates, with the lifetime of picosecond Constantly update synchrotron X-ray sources to enhance the accuracy of recognition
In-situ XAS (i.e., XAFS, including XANES and EXAFS)	Measure oxidation states and electronic configurations of elements (XANES) Track interatomic spacings and coordination numbers (EXAFS)	
In-situ Raman spectroscopy	Monitor structural variations in molecular groups with Raman active	Interface/surface analysis is influenced by the gas–liquid perturbations for both Design special cell to ensure the monitor at the same location and time
In-situ FT-IR spectroscopy	Identify the fragment molecules, radicals, and functional groups	
In-situ TEM (Especially, in-situ aberration-corrected S/TEM, equipped with EELS, EDX)	Observe morphology evolutions Trace atomic structure changes (HRTEM) Exposit structural changes from atomic level (HAADF) Reveal elemental, component, and electronic configuration information (EELS and EDX)	Low-resolution imaging for the liquid electrolyte system Develop cameras with fast electron detection Confirm the integration of rapid chemical detection with fast mapping Customize micro-chip cell
In-situ AFM	Check morphology changes of surface/interface through the tip directly touches to them	Insufficient exposure and contact (AFM), tip size and distance (SECM) limit the resolution Fabricated the new type probe with high sensitivity and wide applicability Modified the suitable in-situ cell
In-situ SECM	Supply electrochemical and topographic changes of surface/interface, without touch	
In-situ ETS	Track surface anion adsorption evolutions (without Raman or IR signal) for special catalyst, based on nano-electronic signals	Limited to metal nano-catalysts surface monitoring Develop applicable conductive matrix to broaden its application
In-situ Mössbauer spectroscopy	Detect nuclei states of special elements, including chemical state, coordination symmetry, spin state, magnetic moment	Severe γ-rays attenuation in a cell with wide size Control the chamber of in-situ cell in a relatively small size

## In-Situ Monitoring the Dynamic Evolution of Catalysts during ORR

**Fig. 5:**
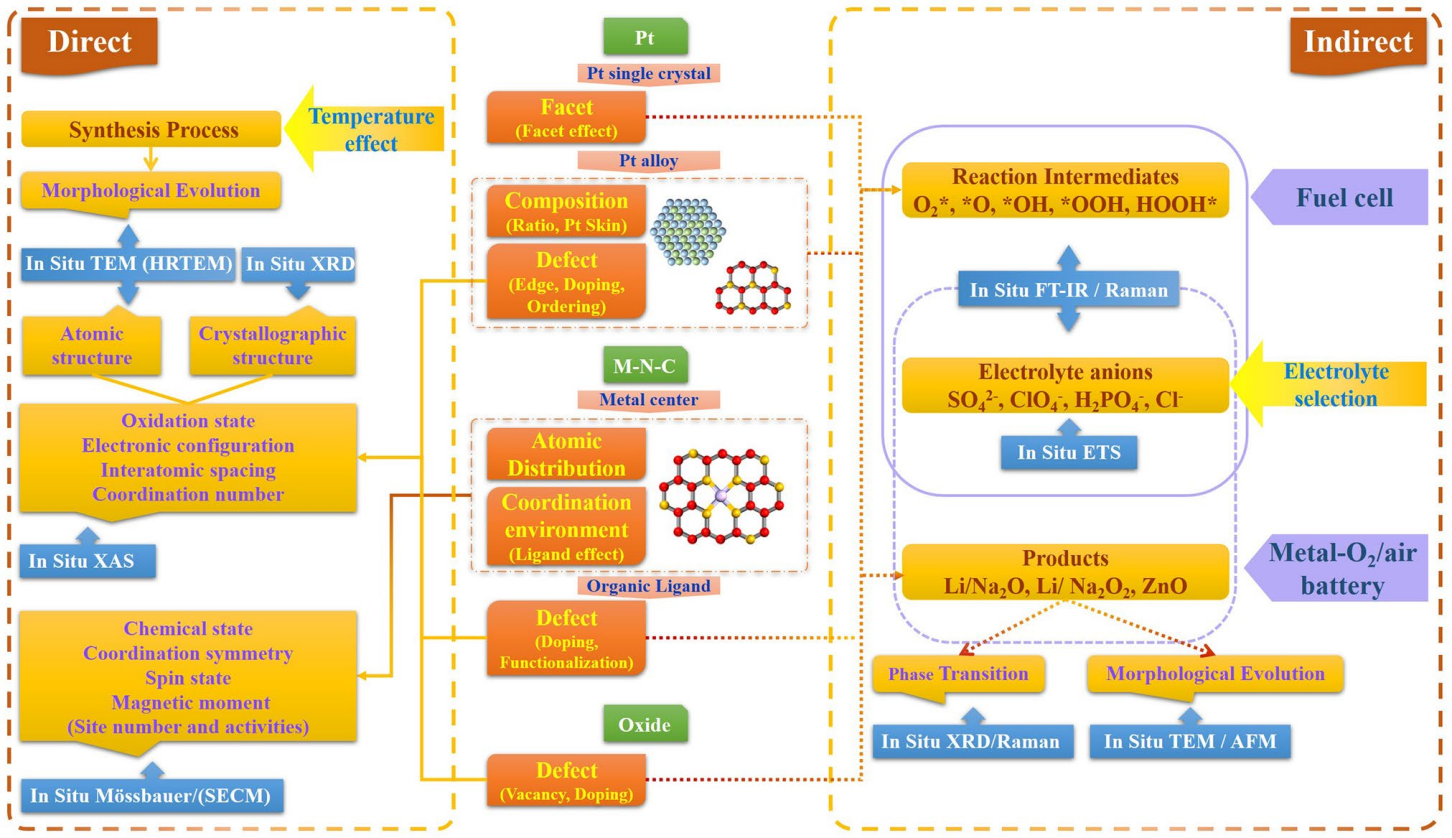
Overview diagram of various direct and indirect in-situ characterization techniques for revealing structural evolution of Pt-based, M–N–C and some oxide catalysts

In this section, we summarize the applications of multiple in-situ characterization techniques for direct monitoring of catalyst dynamic evolution during ORR (Fig. 5, left part). This in-situ revelation can help to elucidate the active site, simultaneously help to guide the synthesis of the catalyst. That is, it can help to realize the reasonable design of catalyst from the source, which in turn promotes the efficient ORR. Notably, the potential values are all relative to the reversible hydrogen electrode (RHE) in the following each section, which will not be repeated later.

### Monitoring of the Phase Transitions

Dynamic detection of crystal phase changes for the catalyst during ORR helps to ascertain active site with efficient catalytic effect in the reaction. It is also useful to identify the optimal structure of catalyst with higher activity, and provides a basis for further adjusting the structure design. Notably, the ADT is the abbreviation of accelerated durability testing in this part and will not be repeated. The correlated works are as follows.

The phase transitions also often occur in the catalyst surface coverings, more notably in the case of rare earth Pt alloy catalysts (i.e., PtxY and PtxGd). Escudero-Escribano et al. [110] investigated the phase transitions of active Pt overlayer on the Gd/Pt (111) single crystalline electrode during ORR in PEMFC by using in-situ synchrotron grazing incidence XRD (GI-XRD). For Gd/Pt (111) R_0_ (i.e., the alloy phase on the crystal with no rotate), in-situ GI-XRD revealed that the compressive strain in Pt overlayer displayed slight relaxation as the cycling gradually entering the relevant region of ORR (i.e., 0.6–1.0 V). After 3000 cycles, the structure of Pt overlayer tended to be stable, and persisted in about 0.8% compressive strain (8.3 Å) after 8000 cycles. This confirmed that the active phase was a compressed Pt overlayer formed on the Gd/Pt (111) electrode, with a relatively stable structure during ORR long cycling. For Gd/Pt (111) R_30_ (with 30° rotation), no structure varies in Pt overlayer were observed in-situ GI-XRD as the cycling from open-circuit potential to 1.0 V. After the cycling potential increasing above 1.2 V, the strain in Pt overlayer relaxed strongly, and up to 1.3 V, its crystalline part thickness increased. This could be attributed to the oxidation of Pt and the leaching of Gd, leading to Pt overlayer becoming rough and loosely thickened. This indicated that the phase transitions of the Pt overlayer were related to the cyclic potential, and the higher potential leaded to its degradation. Thus, this work indicates that Pt overlayer with a compressed strain can act as the main activity site for ORR, also determine the excellent ORR stability, further providing a basis for the construction of catalytic layers on the catalyst surface.

**Fig. 6:**
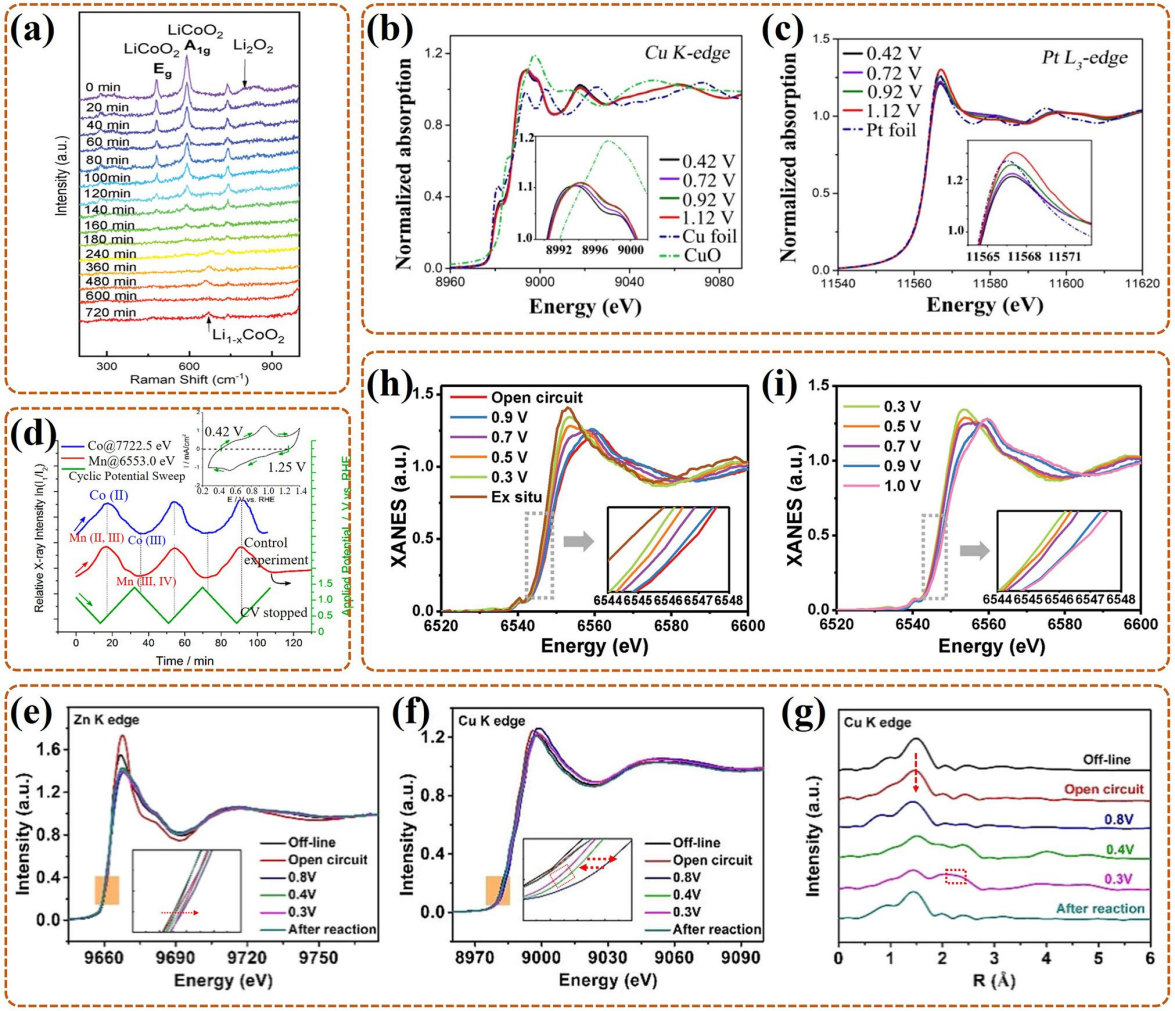
**a** In-situ Raman spectrum of LCO in Li–O_2_ battery during the first recharge [111]; Reused with approval; Copyright 2020 WILEY-VCH Verlag GmbH & Co. KGaA, Weinheim. **b** In-situ Cu K-edge XANES spectrum (with Cu foil and CuO standard as reference), **c** in-situ Pt L_3_-edge XANES (with Pt foil as reference) of Int-PtCuN/KB during ORR in PEMFC [113]. Reused with approval; Copyright 2021 American Chemical Society. **d** The changes of ln(*I*_1_/*I*_2_) for Co K-edge (at 7722.5 eV) and Mn K-edge (at 6553.0 eV) in Co_1.5_Mn_1.5_O_4_/C under the cyclic potential sweep, with the inset of the CV test [84]; Reused with approval; Copyright 2019 American Chemical Society. In-situ XANES spectra of **e** Zn K-edge, **f** Cu K-edge, and **g** in-situ EXAFS spectrum of Cu K-edge, for the Cu/Zn-NC under several constant potentials [115]; Reused with approval; Copyright 2021 WILEY–VCH GmbH. In-situ XANES spectra of Mn-SAS/CN at potentials **h** from 0.9 to 0.3 V (also at open circuit and ex situ), **i** from 0.3 to 0.9 V [116]; Reused with approval; Copyright 2020 WILEY-VCH GmbH

Interestingly, catalysts can not only catalyze phase transitions in ORR, but also participate in phase transitions in reaction. As an example, Gao et al. [111] discovered the phase transitions of LiCoO_2_ (LCO) catalyst by in-situ XRD/Raman and revealed its self-reinforcing effect as a catalyst for Li–O_2_ battery (LOB). In detail, highly ordered LCO as the ORR catalyst exhibited splendid activity during discharge (i.e., ORR), with no phase changes, also accompanied by the accumulation of Li_2_O_2_ products. During charging, the LCO underwent phase change by the extraction of Li^+^ and transformed to LixCoO_2_ phase (i.e., Li_0.6_CoO_2_ (L0.6CO)). As shown in Fig. 6a, Eg bending (O–Co–O) and A1g stretching (Co–O) vibrations of LCO gradually became weaker and changed to A1g stretching (Co–O) vibration of Li_1−x_CoO_2_. Then the formation of L0.6CO induced Li/oxygen vacancy and Co^4+^, which damaged the symmetry of CoO_6_ octahedron, further boosting the oxygen evolution reaction (OER). Synchronously, the L0.6CO reverted to the original phase (LCO), which continued to promote the next ORR. In summary, in-situ analysts of this work revealed that LiCoO_2_ could achieve self-adjustment among LCO and L0.6CO, thus performing higher ORR and OER performance. Thus, the phase changes of LCO catalyst induced by the intercalation or extraction of Li^+^ do exist in ORR, which can modulate the torsional deformation and recovery of the CoO_6_ octahedron, (i.e., active sites), further adjusting the catalysis activity to the best. Also, in-depth research on the self-reinforcing catalysts can bring up new ideas for the design of LOB catalysts.

Notably, the monitoring of phase transitions is an essential descriptor for guiding the reasonable design of ordering degrees for the bimetallic nano-catalyst (BN). For example, Gong et al. [112] used in-situ XAS to probe the varies in local and electronic structure of the *fcc*-phased PtFe ordered alloy (O–PtFe) catalyst during 10 k cycles ADT for ORR in PEMFC. The Pt L_3_-edge XANES exhibited that the O-PtFe was mainly in the metal state (Pt^0^), and with a slight negative shift, proving that Pt was reduced to form an alloy with Fe. This was consistent with the fact in EXAFS that its Pt–Pt bond length (2.708 Å) was shorter than that of pure Pt (2.775 Å). The smaller coordination number (CN) of Fe (ca. 2.6) than Pt (ca. 7) further offered evidence that the O-PtFe surface was Pt-rich. These testified that the ultrathin Pt shell phase formed on the surface of O-PtFe, as the main activity site, for the splendid ORR activity (0.68 A mg^−1^_Pt_) than that of the disordered PtFe alloy (D-PtFe). In-situ Pt L_3_-edge XANES and EXAFS revealed that the ordered structure of O-PtFe with the ultrathin Pt shell was largely preserved, both in peak position and intensity. While the Fe K-edge XANES spectra showed a slight decrease in the surface Fe leaching rate, proving that the ultrathin Pt shell layer ensured the chemical stability of O–PtFe to Fe leaching. Thus, the monitoring of phase transition indicates that the O–PtFe can exhibit excellent ORR activity and stability than D-PtFe due to the formed ultrathin and stable Pt shell phase. Namely, improving the ordering degree is a good method to modify BN, for higher ORR performance.

The role of this descriptor (i.e., the monitoring of phase transition) for the BN is further applied in the N-doped bimetallic nano-catalyst (N-doped BN). Zhao et al. [113] exploited the local and electronic structure changes of the N-doped rhombohedral ordered PtCu catalyst loaded on Ketjenblack (KB) (i.e., Int-PtCuN/KB) during ORR in PEMFC by in-situ XAS. In detail, some weak oxidations of Cu in Int-PtCuN/KB were proved by in-situ Cu K-edge XANES (Fig. 6b) with white line exhibiting slight positive shift as potential increasing, but the varies were enough less than Int-PtCu/KB (without N) and D-PtCuN/KB (disordered). The results testified that the more ordered structure and N dopants in PtCu BN were favorable for increasing the intrinsic corrosion resistance, and promoting the formation of Cu–N bond to further improve the corrosion resistance, respectively. In-situ Pt L_3_-edge XANES of Int-PtCuN/KB (Fig. 6c) reflected a little enhanced main peak (white line) along with increased potential, which was induced by some Pt oxide, in line with EXAFS analysis (i.e., with a weak Pt oxide phase peak at 1.6 Å). Compared to Int-PtCu/KB and D-PtCuN/KB, the little varies of Pt oxide phase transitions revealed that the Pt oxidation on its surfaces was prohibited, contrarily, more effective Pt active sites were exposed. This confirmed that the Pt monolayer shell was formed on Int-PtCu/KB surface, also effectively alleviating the corrosion of Cu. Further, the distance of Pt–Pt bond was 2.693 Å in Int-PtCuN/KB, which is shorter than 2.701 Å (IntPtCu/KB). This meant the presence of N dopants introduced compressive strain on the Pt surface in BN, as the main activity site, to modulate the intermediates adsorption, thus improving ORR activity. This work offers worthy insights to the design of the BN that is to ensure the synergistical effect of ordered structure with N-doping on ORR.

These conclusions declare that the correlation between the catalyst nanostructure and its ORR performance can be revealed by in-situ monitoring of and the phase transitions of the surface coverings and catalyst itself. In detail, the catalyst with little Pt overlayer [110], or higher orderliness [112, 113], dopant [113], etc. are beneficial to the ORR performance. Also, the direct changes in electronic structure, and lattice parameter, are more intuitive to show that the defect and strain are favorable for catalyst activity enhancement.

### Monitoring of the Valence Variations

For some special catalyst with complex valence, mainly transition metal-based catalyst, the determination of its effective active center depends on the in-situ dynamic monitoring of the chemical valence states during ORR. Thus, the more studies focus on it, and some relative works will be summarized in this part.

For the typical single transition metal-based catalyst, it often undergoes valence changes during its catalytic process. Such as Qin et al. [114] explored the effect of the valence of Co in a polypyrrole (PPy)-revised carbon-loaded metal Co catalyst (Co-PPy-BP) towards ORR in DBFC by in-situ XAFS and XRD. Firstly, in-situ XANES of Co-PPy-BP evolved into standard Co (OH)_2_, and then into CoOOH, which implied that the valence of Co underwent Co^0^ → Co^2+^ → Co^3+^ during ORR. Namely, both Co^0^/Co^2+^ and Co^2+^/Co^3+^ existed in Co-PPy-BP, and as the active centers for the higher ORR activity. Moreover, in-situ XRD exhibited that two Co crystal peaks (15.7° and 20.1°) were observed at first and gradually disappeared during ORR; finally, two new peaks emerged at 15.2° and 21.61° attributed to CoOOH under a maximum current. This further confirmed that Co (OH)_2_ was largely an intermediate phase, and the lower valence of Co could ensure a richer redox transition, thus as the main active center for favoring the ORR activity. Namely, this work provides a guidance that single transition metal-based catalyst with the lower initial valence is pivotal to ensure the rich redox transition during ORR.

For the dual transition metal-based catalyst, especially for transition metal oxide, in-situ monitoring of valence changes is also key for identifying the effective active center, further revealing the synergistic catalytic effect of dual transition metals on ORR. For example, Yang et al. [84] utilized in-situ XANES to pursue the valence varies of Co and Mn in the synthesized Co_1.5_Mn_1.5_O_4_/C bimetallic catalyst during ORR in PEMFC, and explored the synergistic interaction of Co and Mn. In-situ XANES of Mn K-edge indicated that the Mn valence existed in a lower value during ORR at more negative potentials. Also, linear combination fitting (LCF) of the Mn valence revealed that its average valence decreased from 3.15 to 2.91 as the potential decreased (1.15–0.4 V). Further the fact that Mn (III, IV) largely converted to Mn (II, III), indicating that various Mn could do duty for active centers for ORR. In-situ XANES and LCF of Co K-edge illustrated that the average valence of Co also decreased (2.75 → 2.57), namely, large numbers of Co (III) converted to Co (II), as the potential decreased. This change was synchronized with Mn, and thus Co and Mn were considered as co-active centers for ORR. Under the non-steady state, the valence states of Mn and Co in Co_1.5_Mn_1.5_O_4_/C changed periodically along with cyclic potential and synchronized with each other. As shown in Fig. 6d, the relative X-ray intensities (ln(I_1_/I_2_), I_1_ as incident, I_2_ as transmitted) of Co and Mn changed from minimum (higher valence Co, Mn, at 1.25 V) to maximum (lower valence Co, Mn, at 0.42 V), as potentials declining from 1.4 V (upper limit) to 0.3 V (lower limit). The actual boundary potential of 0.42/1.25 V corresponded to the oxidation/reduction currents for the cyclic voltammetry (CV) (Fig. 6d, inset). That was, Mn (III, IV) → Mn (II, III) and Co (III) → Co (II) occurred at the same time, which further indicated that Co and Mn had a synergistic catalytic mechanism for ORR. Notably, for a single transition metal-based catalyst (e.g., the above work), lower initial valence for the transition metal can ensure excellent ORR activity. For dual transition metal oxide, two high-valence transition metals can act as the bridges for e transfer when O_2_ is reduced to form H_2_O, which is more favorable to ensure the synergistic effect on efficient ORR.

There is also a synergistic effect of two transition metals in the transition metal-based SAC, and this effect can also be reflected by the valence change of the contained metals. Recently, Tong et al. [115] monitored the changes in the electronic structures of Zn and Cu in Cu/Zn bimetallic single atoms complexed with nitrogen-doped carbon (Cu/Zn-NC) catalyst during ORR in PEMFC through in-situ XAS. In-situ Zn K-edge XANES spectrum (Fig. 6e) showed that the main peak occurred a slight positive shift, owing to the electron transform (Zn to Cu) and intermediates adsorption. Notably, the higher valence than + 2 of Zn in Cu/Zn-NC was another evidence of its electron transfer to Cu. However, neither the length nor ligand number of Zn–N bond occurred changes, indicating that Zn was not the active center. In contrast, in-situ Cu K-edge XANES spectrum (Fig. 6f) showed that the main peak underwent a distinct positive shift as potential decreasing, then its energy decreased obviously at a potential of 0.4–0.3 V, and finally, it returned to initial state only with a little positive shift. In-situ Cu K-edge EXAFS spectrum (Fig. 6g) further revealed that the Cu–N bond (~ 1.5 Å) gradually weakened as potential decreasing, while a new Cu-Cu bond (~ 2.2 Å) generated at a potential of 0.4–0.3 V and disappeared after the reaction. This indicated that Cu single atoms (derived from Cu-N_4_) aggregated into Cu clusters driven by an external electric field, and finally returned to initial state (i.e., isolated and dispersed). In conclusion, in-situ results showed that Cu-N_4_ was the main active center for efficient ORR, and during the catalytic process, it changed from atomic dispersion (with Cu–N bond) to cluster (with Cu–Cu bond), with the cooperation of Zn–N_4_, and finally returned to a single atom state (with Cu–Cu bond disappearing). Notably, the cooperation effect of Zn–N_4_ referred to the electron transform of Zn to Cu regulating the Cu^2+^/Zn^2+^ ratio in Cu/Zn-NC, which further drove the high activity. Thus, this work opens a new insight into understanding of synergy mechanism, which can guide the rational design of dual transition metal-based SAC. That is, the synthesized catalyst contains the special transition metal pairs, which can ensure electron transfer from one transition metal to another.

For the transition metal single-atom site (SAS) catalyst, it is also necessary to monitor their valence changes during ORR and thus clarify the active center composition. For example, Han et al. [116] utilized in-situ XAS to pursue the valence changes of Mn-based SAS catalyst with Mn–N_4_ structure (Mn-SAS/CN) during ORR in Zn–Air battery. In in-situ XANES spectrum of Fig. 6h, both the absorption edge and the main peak occurred positive shift under open-circuit, compared to ex-situ condition. Also, both shifted to lower energy as potential decreased, corresponding to an increase in ORR overpotential. Notably, both could shift back, as potential reversed (Fig. 6i). These indicated that more Mn sites were reduced to lower valence states as overpotential increased, correspondingly, their surface OH adsorption decreased while OH^−^ desorption accelerated. Namely, low-valent Mn^L+^–N_4_ was identified as the active center, which easily promoted electron transfer to *OH and facilitated *OH desorption (corresponding the transformation of OH_ads_–Mn^H+^–N_4_/Mn^L+^–N_4_), further exhibiting efficient ORR. Thus, this work provides evidence that the ORR performance of SAS catalyst is correlated with the valence state of the activity center, and it offers an idea for the design of a highly active SAS catalyst through a coordination modulation strategy.

This part reveals that in-situ XAS technique is crucial to the disclosure the valence information in the activity site of the catalyst during ORR. In detail, this technique can effectively monitor the dynamic changes of the electronic structure and partial atomic coordination for the transition metal-based catalyst active center in the working environment. The obtained information can reveal that different catalysts have different optimal valence states contributing to higher catalytic activity. As summarized above, the low valence state makes more contribution in M–N–C like catalysts [114, 116], while high valence is better for applying in transition metal oxides [84]. Moreover, the synergistic effect often existed in dual transition metal systems, with the electron transfer between them [115]. Thus, the design of the catalyst structure should be tailored to these properties.

### Monitoring of the Morphological Evolutions

Not only does the phase and valence state affect the catalytic activity of the catalyst, but also the morphology is an important factor. Also, structural and valence variations are frequently accompanied by morphological changes, thus the development of various in-situ imaging techniques is crucial. There are several types of researches for investigating microstructural transitions of the catalyst at different scales and high temporal resolution during the synthesis process, as reviewed below.

**Fig. 7:**
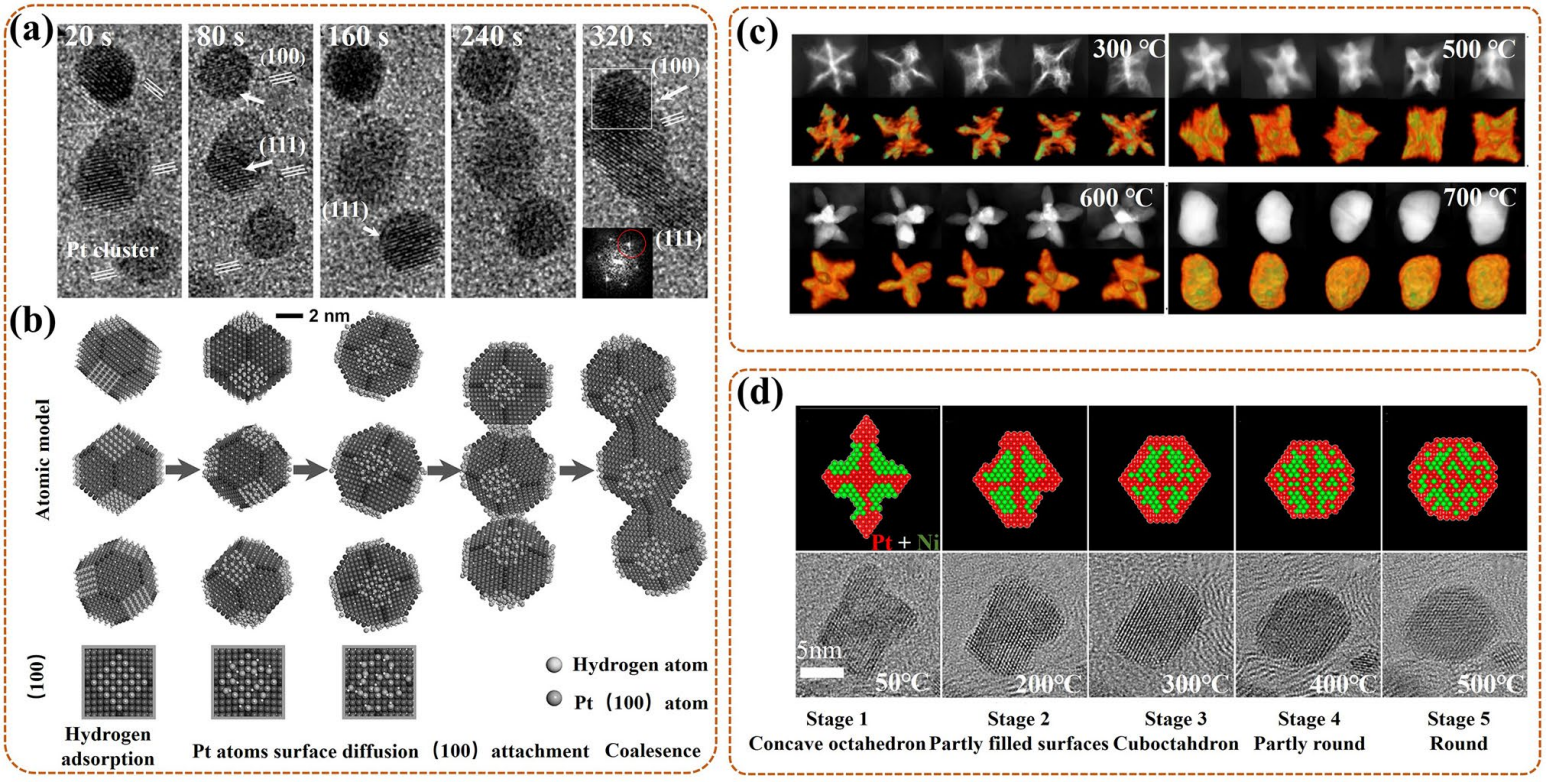
**a** In-situ TEM images at 20 s, 80 s, 160 s, 240 s, 320 s, and **b** the corresponding oriented attachment schematics, for the formation of Pt-NW [117]; Reused with approval; Copyright 2017 WILEY-VCH Verbg GmbH&Co. KGaA, Weinheim. **c** In-situ TEM images of single Pt–Cu NF with 3D visualization at 300 °C, 500 °C, 600 °C, 700 °C, with Pt (red), Cu (green), and orange derived from color overlay [96]; Reused with approval; Copyright 2021 Elsevier Ltd. **d** The schematics (upper) of Pt (red) and Ni (green) distributing, and the corresponding in-situ HRTEM images (bottom) of PtNi_1.5_ NP at 50 °C, 200 °C, 300 °C, 400 °C, 500 °C [118]. Reused with approval; Copyright 2018 American Chemical Society. (Color figure online)

Many in-situ morphological monitoring efforts have mustered on the application of synthesis process for the ORR catalysts. This is crucial to reveal the synthesis mechanism, which can help to offer guidance for optimizing the synthesis strategy, and further synthesizing high activity catalysts. As an example, Ma et al. [117] utilized in-situ TEM to view the oriented attachment process of Pt nanoparticles (NPs) on the (100) lattice planes in the synthesis of one-dimensional (1D) Pt-based nanowires (NWs) catalysts by hydrogen assisted solid-phase method. In detail (Fig. 7a), the intermediate products were collected for in-situ monitor at different reaction time during the synthetic process. At 20 s, three Pt NPs were in a different orientation, with Pt (111) fringes as reference. At 80 s, the upper Pt NP rotated to turn its (100) lattice plane toward the middle Pt NP, then approached at 160 s, attached at 240 s, and coalesced at 320 s. The same process was done for the bottom Pt NPs. Notably, the growth kinetics, including rotation, approach, attachment, and coalescence, of the NPs during the synthesis were related to the Pt surface modifications. Concretely (Fig. 7b), H_2_ preferentially adsorbed on the (100) lattice plane of Pt NP (upper and bottom), forming activated Pt–H complexes. Then special Pt–H ensured a lower diffusion potential barrier, accelerating the local diffusion rate of the modified (100) lattice plane. Finally, the attachment and coalescence tended to occur in this specific plane. In conclusion, the synthesis mechanism of 1D Pt-based NWs was that the oriented attachment of solid-state Pt NP with aid of the metal surface diffusion, which was induced by the adsorption modification of hydrogen molecules. Thus, this work further provides the scalable insight for the preparation of 1D Pt-based NWs with excellent activity for catalytic ORR in PEMFC.

For the Pt-based intermetallic catalyst, in-situ visualization of the morphological changes during the ordered process is essential to reveal the mechanism of its ordering, further helping to reconstruct its structure. Gong et al. [96] used three-dimensional (3D) tomography assisted in-situ STEM to monitor the morphological changes of the Pt-Cu nano-frame (NF) catalyst during the ordering process under in-situ heating conditions. Concretely (Fig. 7c), under 300 °C, the single Pt–Cu NF had no deformation, owing to no interatomic migration and rearrangement; but a slight Pt segregation formed on the surface induced by the high surface energy of nanomaterials. Heating to 500 °C, the NF occurred a slight shrinkage, especially, since its concave part shrank/clustered rapidly, gradually forming an octopod structure. Next to 600 °C, the NF exhibited the most evident octopod structure, with uniformly dispersed Pt and Cu on its surface, also, with a formed Pt skin. Final to 700 °C, the NF entirely evolved into solid nanoparticle (NP), also with a formed Pt–Cu@Pt (i.e., Pt-Cu wrapped by Pt) core–shell structure. Notably, at 660 °C, the transient-state NP suddenly changed into a molten state, at which the atoms migrated rapidly and rearranged into the ordered phase. This ordered process improved the resistance of NF to Cu leaching, also boosting the stability of the Pt-Cu NF catalyst for ORR in PEMFC. In conclusion, high temperature treatment could reduce atomic migration barrier, accelerate atomic diffusion, further promoting the ordering of NF. However, higher temperatures could induce overmuch atomic migration, further leading to the collapse and aggregation of NF structure. Thus, a reasonable temperature selection is conducive to the preparation of a highly active Pt-based intermetallic NF catalyst. Also, it is necessary to exploit the more available method to control the rate of atomic diffusion thereby realizing the controllable synthesis of the ordering Pt-based intermetallic catalyst.

For the Pt-M NP catalyst, considering that its catalytic activity is ascertained by the chemical structure and composition of the outmost atomic layer, it is crucial to in-situ monitor the dynamic morphological changes of them during the synthesis process for devising more efficient ORR catalyst. Gocyla et al. [118] adopted in-situ TEM to observe the morphological evolutions of outmost atomic layer for the octahedral PtNi_1.5_ nanoparticle (PtNi_1.5_ NP) catalyst in in-situ heating synthesis process. Specifically, at initial 50 °C, the PtNi_1.5_ NP had the concave octahedral structure, with rich Pt edges and rich Ni facets (Fig. 7d). Heating to 200 °C, the octahedra were partially filled with the Pt {111} [100] facets, as Pt diffused from edges to the {111} facets. Next, a truncated octahedron gradually formed, with flat Pt {111} facets and additional Pt {100} facets, as Pt continued to diffuse. Up to 300 °C, a cuboctahedron with thin Pt shell gradually formed, during which the diffusion process of Pt continuously proceeds, promoting the further truncation, and increasing the Pt {100} surface area. Then, the cuboctahedra with a gradual loss of flat Pt {111} facets, exhibited in rounding in partial facets at 400 °C, and eventually forming the spherical NP at 500 °C. In summary, the morphological evolution sequence of the PtNi_1.5_ NP followed concave octahedra, octahedra partially with Pt {111} facets (further to truncated octahedron), cuboctahedra, cuboctahedra with partial rounding facets, and spherical NP. Notably, the flat Pt {111} facet could act as the main component for a thin Pt shell, further boosting the catalytic activity. Thus, this work proves that optimizing the ratio of Pt {111} and Pt {100} facets, and adjusting an appropriate thickness of Pt shell can provide synthetic possibilities for the synthesis of highly stable and active Pt-M NP catalyst for ORR in PEMFCs.

Similar research is the work of Dai et al. [119], they also used in-situ TEM to check into the surface composition of the disordered Pt_3_Co NP, and the morphological dynamic changes of its surface during in-situ heating process. Specifically, the Co segregation occurred on Pt {111} rather than on Pt {100} surface. Then, a few Co oxidation to form CoO layer occurred on the outermost Pt {111} surface, blocking the exposure of the underlying Pt in O_2_, and preventing its diffusion and reconstruction. In contrast, the Pt {100} surface possessed oxidation resistance, as a pivotal role to preserve the ORR activity of the disordered Pt_3_Co NPs catalyst. In brief, the Pt {100} surface was the main active composition for the disordered Pt_3_Co NP. Compare to the above work of PtNi_1.5_ NP, it could be concluded that the main active composition for the Pt-M NP catalyst was relative with the element (Ni, or Co, etc.) alloyed with Pt. Thus, the real time monitoring of dynamic evolutions of surface composition and morphology can provide precise information about the active site during in-situ heating synthesis process, further providing a new light (with a rich Pt surface) on the design of the Pt–M NP catalyst.

For the non-platinum metal-based catalyst, such as ZIF-derived catalyst with high activity in fuel cell, the actual pyrolysis process is also usually simulated under in-situ TEM heating test to reveal the relationship of morphological changes and catalytic activity. Wang et al. [120] applied ZIF-67 as a matrix to investigate the effect of temperature on the microstructure evolutions of derived catalyst at every stage of pyrolysis by in-situ TEM. In detail, ZIF-67 remained initial structure at 300 °C, while its ordered structure collapsed and turned to disorder at 442 °C. Then, its unit cell (i.e., CoN_4_ tetrahedron) gradually decomposed at 500 °C, resulting in N loss and Co precipitation. Next, a new Co@N–C structure gradually formed at 550 °C, along with a hierarchical and 3D connected porous structure in carbon matrix, owing to the precipitated Co nanoparticles (NP) grew and catalyzed carbon graphitization. Finally, the matrix eventually occurred carbonization at 800 °C, due to the further growth to large one of Co NP, and further loss of N. Notably, the ZIF-derived Co@N–C catalyst exhibited the excellent ORR activity in PEMFC. The results proved that it was necessary to ensure the suitable N content, graphitization degree, and hierarchical and 3D connected porous structure, for efficient ZIF-derived catalyst. Moreover, the synergistic effect among Co single atom and Co encapsulated by less layer graphene, was also the key factor for its enhanced ORR activity. Thus, this work provides strong evidence that the ZIF-derived catalyst, prepared at relatively low temperatures, has higher performance, also with the energy-saving advantage.

In summary, in-situ TEM can directly observe the morphology varies of the catalyst itself during the synthesis process in real time, further providing some crucial information for guiding the design of more efficient ORR catalyst. Specifically, the special way, such as the hydrogen assisted solid-phase method, is essential for the synthesis of the special morphology catalyst. The rational pyrolysis temperature is proven to be important for PtM NP catalyst, which can adjust the crystal face ratio or enrich the Pt skin for high activity NP; also, it is essential for regulating N content, pore structure and graphitization for M–N–C catalyst.

### Monitoring of Electronic Structure

Many works have confirmed that the maximum exposure of active sites and the increase of intrinsic activity in each active site are key tactics to elevate the ORR activity of catalyst. Although massive progress has been acquired in the design of catalysts through the two strategies, the changes in the electronic structure of the active site remain controversial. In this part, the progress of in-situ techniques applied to identify the related changes is reviewed.

**Fig. 8:**
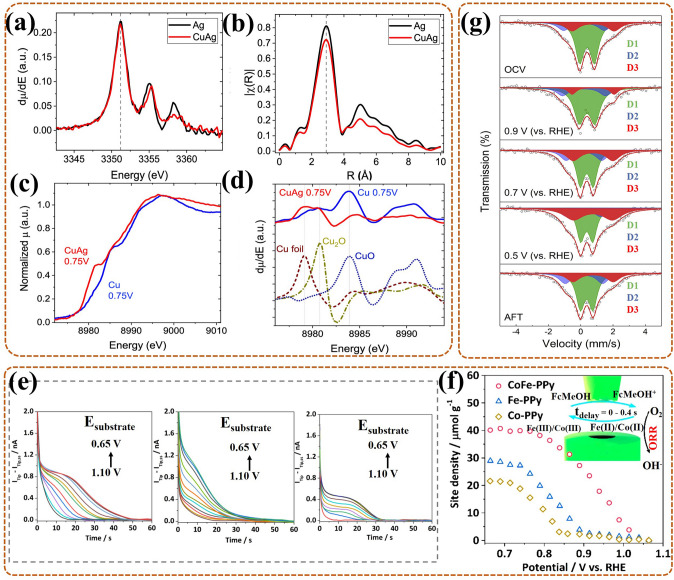
**a** The derivatives of in-situ Ag L_3_-edge XANES, and **b** the relevant EXAFS, for CuAg and Ag during ORR at 0.75 V; **c** In-situ Cu K-edge XANES spectra, and **d** the derivatives of XANES with Cu standard as reference, for CuAg and Cu also at 0.75 V [121]; Reused with approval; Copyright 2022 Wiley-VCH GmbH. **e** Titration curves from left to right for CoFe-PPy, Fe-PPy, and Co-PPy in FcMeOH (0.5 mM), KOH (0.01 M) and NaClO_4_ (0.1 M), respectively; **f** The curves of titrated site density against substrate potential, with inset for the schematic of in-situ SECM cell [122]; Reused with approval; Copyright 2019 American Chemical Society. **g** In-situ ^57^Fe Mössbauer spectra of Fe–NC–S with ^57^Fe during ORR under various potentials; the OCV was open-circuit voltage, the AFT was after ORR [109]; Reused with approval; Copyright 2020 Elsevier

Typically, the activity of the bimetallic catalyst often exceeds that of any single metal, but the real activity site of the catalyst for its enhanced performance always remains unclear. Considering that the real activity site is always related to the electron transfer between the two metals, it is essential to monitor the electron structure changes of the catalyst. Such as, to ascertain the active site of the CuAg catalyst, Gibbons et al. [121] monitored its electron transfer behavior via in-situ XAS during ORR. In-situ Ag L_3_ edge XANES of CuAg (Fig. 8a) (compare with Ag) showed that the main peak (~ 3353 eV), owing to Ag 2*p*_3/2_ electron being excited to its first non-occupied level, was unaffected by Cu, with almost no electronic changes, at 0.75 V. Other peaks (~ 3369, 3377, and 3397 eV) varied in intensity and position with or without Cu, which were caused by Cu scattering rather than Ag electronic state, thus further indirectly proving that the electronic state of Ag in CuAg with no changes. The relevant in-situ EXAFS of CuAg (Fig. 8b) showed that the first-shell peak (~ 2.9 Å) had no tensile strain, and with no peak of Ag–Cu interaction, only with a decrease of the extended peak (~ 5 Å) due to Cu-induced lattice deformation, at 0.75 V. This meant that there was no obvious change in Ag geometric state of CuAg. Conversely, in-situ Cu K-edge XANES of CuAg (Fig. 8c) (compare with Cu) showed that the edge peak shifted by a few eV, at 0.75 V. The XANES with Cu standard as reference (Fig. 8d) showed that the Cu peak in CuAg was like a mixing of metal Cu^0^ and Cu^+^ oxide, while the Cu peak in pure Cu was like standard Cu^2+^ oxide, thus Cu in CuAg existed in a more reduced state. Thus, the electronic state and local bonding of Ag in CuAg remained unchanged, while the electronic state of Cu changed dramatically, thus the high activity of CuAg catalyst was due to electronic rather than geometric effect. That is, it was more useful to create a Cu-center active site than to add an Ag-center active site for high activity CuAg bimetallic catalyst for ORR. This gives an idea of the identity of the real active site of the bimetallic catalyst.

When it comes to specifying the number of active sites in bimetallic catalyst, in-situ SECM often plays an important role and is to be used for quantification. For example, Li et al. [122] adopted in-situ surface-interrogation SECM (Fig. 8f, inset) to quantify active sites of the CoFe-PPy catalyst (Co and Fe highly distributed on polypyrrole (PPy) derived C), revealing the reason for its high activity. In-situ SI-SECM titration curves (Fig. 8e) of catalysts all showed obvious growth in feedback currents as substrate potentials in more negative, indicating that active intermediates gradually formed and increased. Then, the plots of site densities against applied potentials (Fig. 8f) revealed that, for CoFe-PPy, active intermediates formed at the lowest overpotential, also with the highest site density (40.68) in it than Co-PPy (28.2) and Fe-PPy (21.57) at 0.8 V. Moreover, the catalytic kinetics quantified by in-situ SI-SECM exhibited that, for CoFe-PPy, the binding rate to O_2_ was much faster than Co-PPy and Fe-PPy. Consequently, the bimetallic CoFe-PPy was testified to have the largest number of active sites, and each site with higher intrinsic activity, so it has the excellent activity. Thus, this work gives high-resolution proof to quantify not only the active site density, but also the kinetic rate, for the bimetallic catalyst.

For Me^n+^/Me^(n+1)+^ series of catalyst, the catalytic OOR process is often accompanied by electron transfer behavior, as mentioned in Sect. 4.2. However, there are exception, and it is more important for such exception to identify their active sites. For example, in the work of Wang et al. [123], the polypyrrole-based carbon-loaded cobalt oxyhydroxide (CoOOH-PPy-BP) catalyst did not undergo any electron transfer in the catalytic ORR of DBFC. Considering that the active site was not the conventional Co^n+^/Co^(n+1)+^, they used in-situ XRD and XAS to confirm the real effective active site. The results showed that no phase transitions or valence varies were recognized during the reaction. Contrarily, oxygen vacancy was markedly detected by the Fourier transformed k^2^-weighted EXAFS function analysis. Additionally, they proposed a new ORR mechanism, namely, the electron hole arising from oxygen vacancy captured electron from the anode further forming [Co^3+^ + e], then, the adsorbed O_2_ (on the vacancy) captured electron from [Co^3+^ + e] and occurred in reduce. In conclusion, this work testifies that the oxygen vacancy can sometimes substitute Co^n+^/Co^(n+1)+^ as the active site for the corresponding transition metal-based catalyst. Also, it lights a thought for designing catalysts with higher ORR activity by artificially introducing oxygen vacancy.

Notably, for the M–N–C catalyst, especially for the Fe–N–C catalyst, the exploration of its active site has been recognized as the meaningful work. With the development of technique, the special in-situ ^57^Fe Mössbauer spectroscopy become the most powerful monitoring method. Li et al. [124] adopted this technique to identify the real active site for Fe–N–C catalyst, and studied the contribution of different active sites to its catalytic activity and stability. In-situ ^57^Fe Mössbauer spectra revealed that it contained high-spin (HS) S1 site (identified as HS FeN_4_C_12_) and low- or medium- spin (L or MS) S2 site (identified as L or MS FeN_4_C_10_) for contributing to the catalytic activity in Fe–N–C catalyst. In the subsequent reaction, S1 was degraded by conversion to iron oxide (III/II), namely a direct/indirect process of demetallation, in which the indirect route was initiated by the local oxidation of carbon surface or the protonation of basic N in S1. While S2 remained unchanged in structure and quantity, and still with obvious contribution for high ORR activity after 50 h of operation. That was due to S2 with more locally graphitized structure, less reactive oxygen species (ROS) product, or due to an active carbon top surface. In conclusion, there existed two active sites for Fe–N–C catalyst, both of which contributed to ORR activity in the early stage, while only S2 with contribution in late stage. Thus, this work indicated that the special in-situ ^57^Fe Mössbauer technique did can act as the impactful way for ascertaining and tracing the active site for Fe–N–C catalyst during ORR in fuel cell. And it offers support that the S2 site, i.e., L or MS FeN_4_C_10_, is more significant for the development of Fe–N–C catalyst, which should be emphatically considered in the synthesis process.

Similar, in-situ ^57^Fe Mössbauer spectroscopy was also used by Li et al. [109] to identify the effective active sites and monitor their evolutions of Fe–NC (with four N-coordinated), FeNC–S0.2 (with 0.2 mL 1H-1,2,3-triazole), Fe–NC–S0.4 (0.4 mL), and Fe–NC–S (1.5 mL, with six N-coordinated) catalysts for the ORR of PEMFC. Taking Fe–NC–S with the highest ORR activity as an example, in-situ ^57^Fe Mössbauer spectra (Fig. 8g) showed that three Fe electronic states all existed in each spectrum at different potential. In detail, the low-spin (LS) Fe^2+^(D1), medium-spin (MS) Fe^2+^ (D2), and high-spin (HS) Fe^2+^ (D3) were attributed in sequence to Fe^II^N_4_C_12_, Fe^II^N_4_C_10,_ and N-Fe^II^N_4_C_10_. At 0.9 V, the D3 exhibited in decrease with the relative increase of D1, reflecting that O_2_ adsorbed on the D3, also forming a O_2_^−^–Fe^II^N_5_ intermediate. At this stage, the Fe–N bond gradually shorten with the approaching of Fe in N–Fe^II^N_4_C_10_ to N_4_-plane, also with the electronic state conversion of Fe^2+^ from HS to LS. Conversely, at 0.7 V and 0.5 V, the D3 exhibited in gradual increase with the relative decrease of D1, reflecting that O_2_ adsorbed on the D1, also forming a O_2_^−^–Fe^II^N_4_ intermediate. Here, the Fe–N bond gradually lengthen with the moving of Fe in FeN_4_C_12_ away from N_4_-plane, synchronously, the LS Fe^2+^ returned to HS. These results showed that there existed three activity sites and occurred dynamic cycle during ORR, also accompanied by the formation of some correlated intermediates. Thus, this work confirms the importance of in-situ Mössbauer technique, and points out that the dynamic cycle of the sites is crucial for the reaction process. That is, the design of the catalyst can focus on the integration of different sites.

Moreover, for the modified Fe–N–C catalyst, such as with heteroatom doping, has shown the excellent ORR activity, also with different effective active site than conventional Fe–N–C catalyst. Thus, exploring the active site of the modified Fe–N–C catalyst is a necessary work, also can help to optimize idea for catalyst modification. Recently, Chen et al. [125] used in-situ ^57^Fe Mössbauer spectroscopy to ascertain the effective active site of sulfur(S)–doped Fe_1_–NC catalyst for ORR in fuel cell. The results showed that the spin-polarized configuration of Fe_1_-NC occurred conversion with the adding of S in its second coordination sphere. And the active site of the S–doped Fe_1_–NC catalyst was identified as the low spin (LS) single-Fe^3+^-atom in C–FeN_4_–S part. Moreover, this LS Fe site could facilitate the desorption of OH*, further boosting the ORR activity. In conclusion, S-doping could improve the catalytic activity of the catalyst by modulating the spin state of the central Fe atom in Fe1–NC. This provides a new insight to clarify the effect of heteroatom on improving the ORR activity for the Fe–N–C catalyst. Also, this work can act as a reliable basic for the design of heteroatom doped Fe–N–C catalyst.

In this part, advanced in-situ techniques have been shown to straightly observe the active site structure of the catalyst, particularly, even the spin state of Fe site in Fe–N–C like catalyst can be clarified by in-situ Mössbauer spectroscopy. In addition, the electronic structure and number of the active site in catalyst are proved to be related to its intrinsic property, thus, the detection of electron transfer can also promulgate the structure of the active site in reverse. More importantly, some active sites, such as vacancies, atom-dispersed active sites with certain spin states, heteroatom doping sites, have been identified with more contribute to the high ORR activity of the prepared catalyst.

To sum up, the application of in-situ X-ray technique (XRD, XAS), electron technique (TEM, HRTEM), scanning probe technique (SECM), and Mössbauer spectroscopy can probe the phase, valence, morphology, and electronic structure varies of the catalysts in real time (Fig. 1). These are helpful to determine the active site of the catalyst and provide direction for the structural design of the catalyst. Specifically, the specific dynamic changes of the structure, morphology and electronic states of the catalyst corresponding to different in-situ techniques are depicted in Table 2. In addition, in-situ TEM monitoring of the morphology evolution is helpful to determine the reasonable synthesis conditions and to better synthesize the catalyst from the source. It has a spatial resolution of up to 0.1 nm, unlike the other two in-situ scanning probe technique techniques (SECM mentioned above; AFM to be mentioned soon) with morphological characterization capabilities. The uniqueness of each technique is also reaffirmed as above works.

**Table 2:** Capabilities of different in-situ techniques for probing the essential information to identify actives sites and reveal the ORR mechanism

Techniques	Information provided					
	Phase	Electronic states	Valence	Morphology (spatial resolution)	Others	
In-situ XRD	Crystalline phase varies	–	–	–	Changes in the characteristic peak intensity after intermediates adsorption Related crystal phase changes of the products	
In-situ XAS	Microscopic electronic structure varies and their induced phase variations			–	–	
In-situ Raman spectroscopy	New bonds and their vibration changes in the catalyst			–	Molecular vibrational varies of the relevant molecular groups of intermediates on the catalyst surface	
In-situ FT-IR spectroscopy	Vibrational changes of the functional group contained in the catalyst			–	Infrared vibration signal changes of adsorbed groups of intermediates on the catalyst surface	
In-situ TEM	Changes in the structure of selected atoms and even a single atom		–	0.1 nm	Morphological varies and the atom structure varies of the products	
In-situ AFM	–	–	–	nm	Morphological changes with the characteristic structural labels of the products	
In-situ SECM	–	–	–	10 nm–1 µm		
In-situ ETS	–	–	–	–	Varies in currents after the halogen anions adsorption	
In-situ Mössbauer spectroscopy	Changes in nuclei states of Fe, Sn, Ru, Au etc., especially for Fe–N–C		–	–	–	–

## Applying In-situ Characterization Techniques to Revealed the ORR Mechanism

As is well known, the ORR of the fuel cell is an extremely complex process, which can be two-step pathways (with two electrons transform) or one-step pathways (with four electrons transform) (Fig. 2a). The latter is the preferred pathway, and there exist two possible mechanisms, namely, association or dissociation. Any reaction pathway involves various evolutions of reaction intermediates. Thus, it is essential to monitor and capture the evolution state information on intermediates in a hurry, further facilitating the clarification of the ORR mechanism. In addition, the ORR of the metal–O_2_/air battery is also a complex reaction process with abundant catalytic products (Fig. 2b). Timely capture of the interrelated evolution state information on products is also crucial to reveal its mechanism. Meanwhile, in-situ monitoring process of reaction intermediates and products is also conducive to indirectly reveal the factors affecting the activity of the catalyst, and guide its structure design (Fig. 5, right part). In this section, many relevant in-situ characterization procedures are reviewed below. Notably, the markings in the following works, including ad/ads and *, all mean the surface-adsorbed, that will not be repeated later.

### Monitoring of the Evolutions for Reaction Intermediates

The evolution state information on reaction intermediates can directly help to exposit the specific ORR pathway. Also, the information can indirectly help to identify active site and reveal the reason for enhanced activity, which in turn can help in the design of the catalyst with two or four-electron pathway facilitation for applying in fuel cell. Moreover, considering that the intermediates have characteristics of short survival, low coverage, and vulnerability to other co-adsorbed species, it is essential to use in-situ techniques with high sensibility and quick respond capacity to monitor their dynamic evolutions. The following provides an overview of the representative efforts for in-situ monitoring the reaction intermediates.

**Fig. 9:**
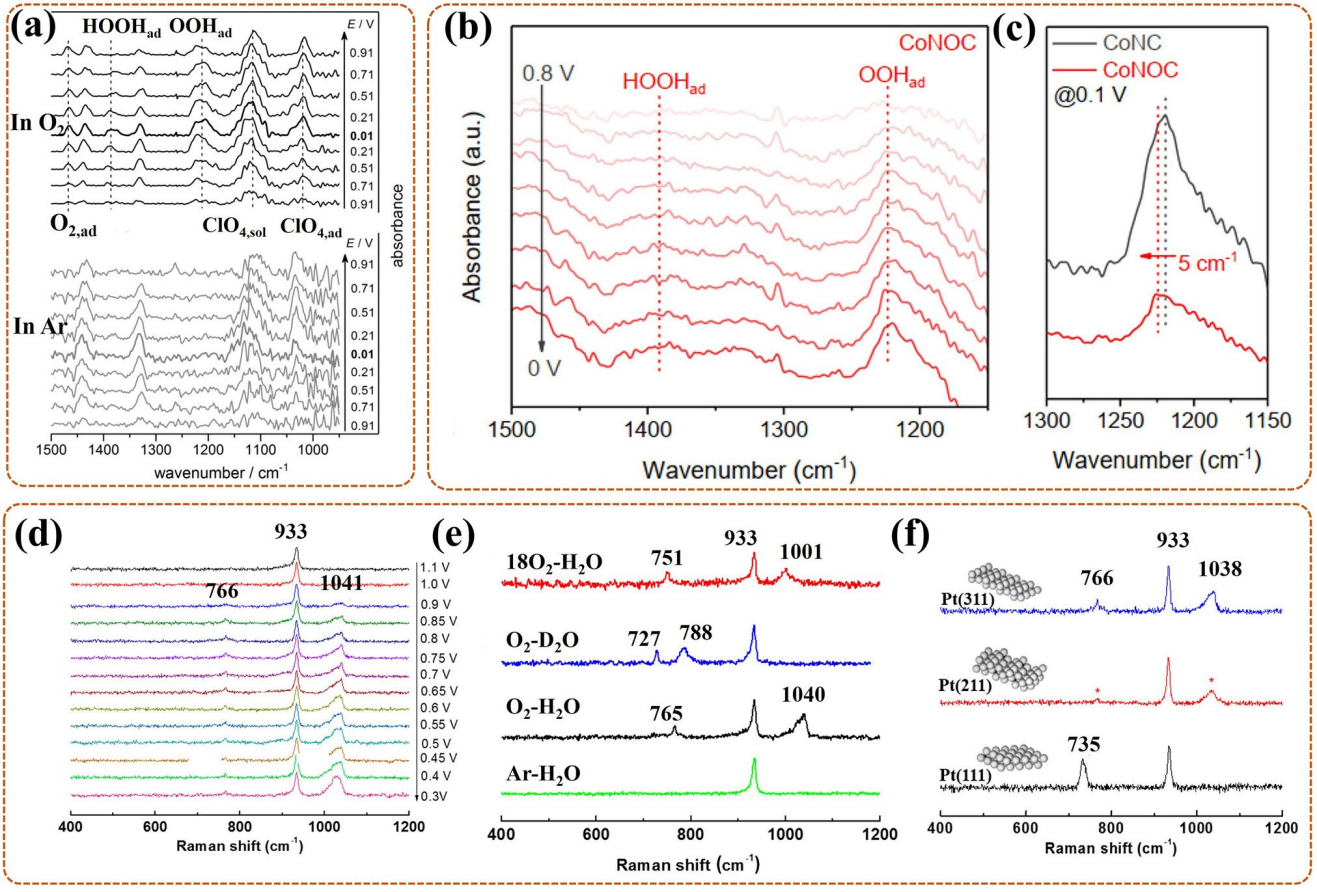
**a** In-situ IR spectra of Pt/C in 0.1 M HClO_4_ in O_2_ and in Ar [93]; Reused with approval; Copyright 2018 WILEY-VCH Verbg GmbH&Co. KGaA, Weinheim. **b** In-situ ATR-IR spectrum of CoNOC in 0.1 M HClO_4_ with O_2_ saturated; **c** The comparative spectrum of CoNOC and CoNC in the band near 1220 cm^−1^ [128]. Reused with approval; Copyright 2021 American Chemical Society. **d** In-situ SHINERS spectrum of Pt (311) in 0.1 M HClO_4_ with O_2_ saturated; **e** The isotopic control spectra on the Pt (311) surface, at 0.7 V, in aqueous with ^18^O_2_ (red curve), in deuterated 0.1 M HClO_4_ (blue curve); **f** In-situ SHINERS spectra of different Pt (hkl) surfaces at 0.8 V [129]; Reused with approval; Copyright 2020 American Chemical Society. (Color figure online)

Typically, monitoring the dynamic evolutions of intermediates on Pt/C catalyst during catalytic ORR is useful to ascertain their state and lay the basis for clarifying the complex pathways of the reaction. For example, to explore the surface chemistry changes on the surface of Pt/C catalyst, Nayak et al. [93] used in-situ ATR-IR to monitor the ORR process in fuel cell. In-situ IR spectrum showed that three oxygen-containing intermediates adsorbed on the Pt outside surface during ORR in 0.1 M HClO_4_ (O_2_-saturated) (Fig. 9a). Compared to bare carbon carriers, the three peaks were proved to belong to the O–O stretching model (1212 ± 3 cm^−1^) in superoxide (OOH_ad_), the OOH bending model (1386 ± 4 cm^−1^) in hydroperoxide (HOOH_ad_), and the O–O stretching model (1468 cm^−1^) in weakly adsorbed oxygen (O_2, ad_), respectively. Further, compared to in-situ IR spectrum of Pt/C during ORR in 0.1 M HClO_4_ (Ar-saturated) (Fig. 9a), it confirmed that the 1114 and 1030 cm^−1^ peaks belonged to the asymmetric ClO_4_ stretching model of the electrolyte and the symmetric ClO_3_ stretching model of the adsorbed ClO_4, ads_, respectively. Next, the 1435 and 1330 cm^−1^ peaks, observed on both Pt/C and bare carbon, were classified as carbon surface functional groups. More, the occasional small peak was discovered at 1260 cm^−1^ due to the Si–O–Si band of the silicone glue for sealing the optic. More importantly, the integral intensity of the intermediates fitted peak varied with the potential, which confirmed that OOH_ad_ and HOOH_ad_ could increase synchronously and dominated in the ORR process under below 0.8 V. This meant that an additional association pathway did exist in the ORR process, and with the greatest contribution at lower potentials. In general, this work gives IR information for the identification of intermediates on Pt/C catalyst surface, also can act as evidence that the association pathway and dissociation pathway often parallel in the ORR.

For the non-metal catalyst, in-situ IR testing is also applicable to ascertain the existed intermediates during its catalytic reaction, further revealing the ORR pathway. For example, Lin et al. [126] used in-situ ATR-IR to monitor intermediates on the outside surface of N-doped carbon catalyst (with various N species) during ORR. In-situ IR revealed that three intermediates, including O_2, ads_ (1468 cm^−1^), O_2_^−^* (1052 cm^−1^), and OOH* (1019 cm^−1^), emerged on the catalyst surface during ORR in 0.1 M KOH (O_2_-saturated). Also, the rate-determining step (RDS) for ORR was ascertained as followed, O_2_^−^* + H_2_O → OOH* + OH^−^. In addition, the pyridinic N was proved to facilitate ORR along a four-electron pathway, with the carbon atom at its adjacent position identified as the main active site. In this work, the IR information about intermediates on N-doped carbon catalyst surface has been supplied and its direct revealing effect for RDS has been testified. Moreover, it can be used as a basis to determine the active site in reverse by monitoring the evolution of the intermediates. Similar work was that Cheng et al. [127] used in-situ synchrotron radiation FT-IR for monitoring intermediates evolution during ORR in real time to precisely identify the active site of the NiFe MOF catalyst. The results showed that the lattice stress was introduced into the NiFe-MOF lattice through a controlled "photoinduced lattice strain" strategy, which could serve as a contributor for the main active site. During ORR, the *OOH (1048 cm^−1^) intermediate was observed to appear at the high-valence Ni^4+^ active site, indicating that the reaction was dominated by the four-electron pathway. This work enhances our awareness of reverse-identification of active site by intermediates monitoring. Further the active site information can help to confirm the structure of the catalyst with the special pathway facilitation.

For the transition metal-based SAC catalyst, the different central atomic coordination environments can lead to different ORR pathway. In-situ surveying the dynamic evolutions of intermediates on the catalyst surface by in-situ IR can help to identify the active site, and reveal the relationship of the coordination environment and the ORR pathway selectivity. Taking Co SAC catalyst as an example, Tang et al. [128] used in-situ ATR-IR to reveal the adsorption behaviors of *OOH on CoNC (with pure N) and CoNOC (with combination of O and N) during ORR in 0.1 M HClO_4_ (O_2_-saturated), further revealing the ORR pathway selectivity. In theory, for CoN_4_ (with pure N), the adsorption site of *OOH was on the Co center, with the ORR dominated in four electrons pathway. For CoN_2_O_2_ and CoO_4_(O), the site migrated from the Co to the C adjacent to O, also the pathway turned to two electrons. In fact, as shown in in-situ ATR-IR spectrum of CoNOC, the 1224 cm^−1^ (main) and 1030 cm^−1^ (weak) peaks were proven to belong to the O–O stretching model of OOH_ad_ and the OOH bending model of HOOH_ad_, respectively (Fig. 9b). Notably, the OOH_ad_ peak of CoNOC emerged at a relatively lower potential and was weaker in intensity, compared to CoNC (Fig. 9c), confirming the existence of OOH_ad_ adsorption migration in CoNOC from Co to C (i.e., new active site), in line with above theories. In summary, the ORR pathway selectivity on Co-SAC could be estimated by the synergistic effect of the modified first coordination spheres (CSs) (with N or/and O), and the modified second CSs (C–O–C groups). Namely, the CoNOC catalyst exhibited ORR catalysis along the two electrons pathway, due to the integration of N, O-dual coordination with C–O–C groups. Different from other above works, this work provides a basis for designing catalysts with specific ORR pathway selectivity at the molecular level.

In addition to in-situ IR spectrum, in-situ Raman spectrum is another effective tool for detecting reaction intermediates, tracking their dynamic evolutions state, and further revealing the ORR mechanism. Typically, Dong et al. [89] monitored the real-time chemistry changes on the surface of the low-index Pt (hkl) single crystal catalyst during ORR in fuel cell by their self-developed in-situ SHINERS. In-situ Raman evidence of intermediates during ORR in 0.1 M HClO_4_ (O_2_-saturated) displayed that, on Pt (111), there existed a stable HO_2_* adsorption (732 cm^−1^). However, for Pt (110) and Pt (100), no HO_2_* adsorption was observed, but the OH* adsorption on their surface all emerged at 1080 cm^−1^, with differences in intensity and initial potential. Thus, the possible ORR mechanism in acid solution could be interpreted as follows, firstly, the O_2_ adsorbed on the Pt surface to form the adsorbed O_2_*. Then the O_2_* combined with ‘H’, also with an electron transfer, forming HO_2_*, which as the main intermediate for Pt (111) needed higher activation energy to further combine with ‘H’ forming H_2_O. Next, the HO_2_* always easily split into OH* and O*, then, the OH* as the main intermediate for Pt (110) and Pt (100) easily further combined with ‘H’ to form H_2_O. Furthermore, in 0.1 M NaClO_4_ (O_2_-saturated), only O_2_^−^ adsorption (1150 cm^−1^) was detected on the surfaces of the three single crystals. Therefore, a series of Raman information of key intermediates (OH*, HO_2_* and O_2_^−^) on the surface of low-index Pt single crystal during ORR were offered in this work. It can be used as the reference information for the detection of intermediates of Pt-based catalysts with Pt (111), (110) and (100) crystal planes, which can further help to reversely explain ORR mechanism.

In their subsequent work [129], the Raman information of intermediates on the high-index Pt (hkl) crystal planes, including Pt (211) and Pt (311), during ORR in 0.1 M HClO_4_ (O_2_-saturated), were further obtained also via in-situ SHINERS. As shown in in-situ Raman spectrum of Pt (311) (Fig. 9d), there existed a stable peak at 933 cm^−1^, due to the ClO_4_^−^. More, the 1041 and 766 cm^−1^ peaks appeared at 0.9 V, then the former became stronger while the latter remained constant, as potential decreasing. Notably, both peaks were not observed in the Ar-saturated HClO_4_ test, so it was confirmed to be related to the ORR process. To clarify the attribution of the both peaks, an amount of isotope labeling experiments were subsequently performed. In the deuteration experiment (Fig. 9e, red curve), the 1041 cm^−1^ peak shifted to the 788 cm^−1^, and the 766 cm^−1^ peak shifted to 727 cm^−1^, reflecting that they were correlated with "H". In detail, the large change for 1041 cm^−1^ peak indicated it belonged to "OH", and the change for the 766 cm^−1^ peak was related to proton interactions, corresponding to "OOH". Then, in the ^18^O_2_ isotope experiment (Fig. 9e, blue curve), the 1041 and 766 cm^−1^ peaks shifted to 1001 and 751 cm^−1^, proving that they were also associated to the "O" atom, further confirming the conclusion of the deuteration experiment. Moreover, both OH and OOH intermediates also existed on Pt (211), and with larger potential-dependent changes, corresponding to higher ORR activity. Different from the low-index Pt (111), the OOH and OH were more susceptive to the high-index Pt (311) and (211) (Fig. 9f). In conclusion, this work provides the Raman information about OOH and OH intermediates, also proves that the ORR activity of the high-index Pt crystal plane is determined by its surface structure and the intermediate adsorption. Further, the isotope labeling experiments are confirmed as a powerful means of revealing classes of intermediates. Thus, this offers a basis for monitoring the evolution of intermediates in real time to guide the design of Pt-based catalysts with different crystal plane structures.

**Fig. 10:**
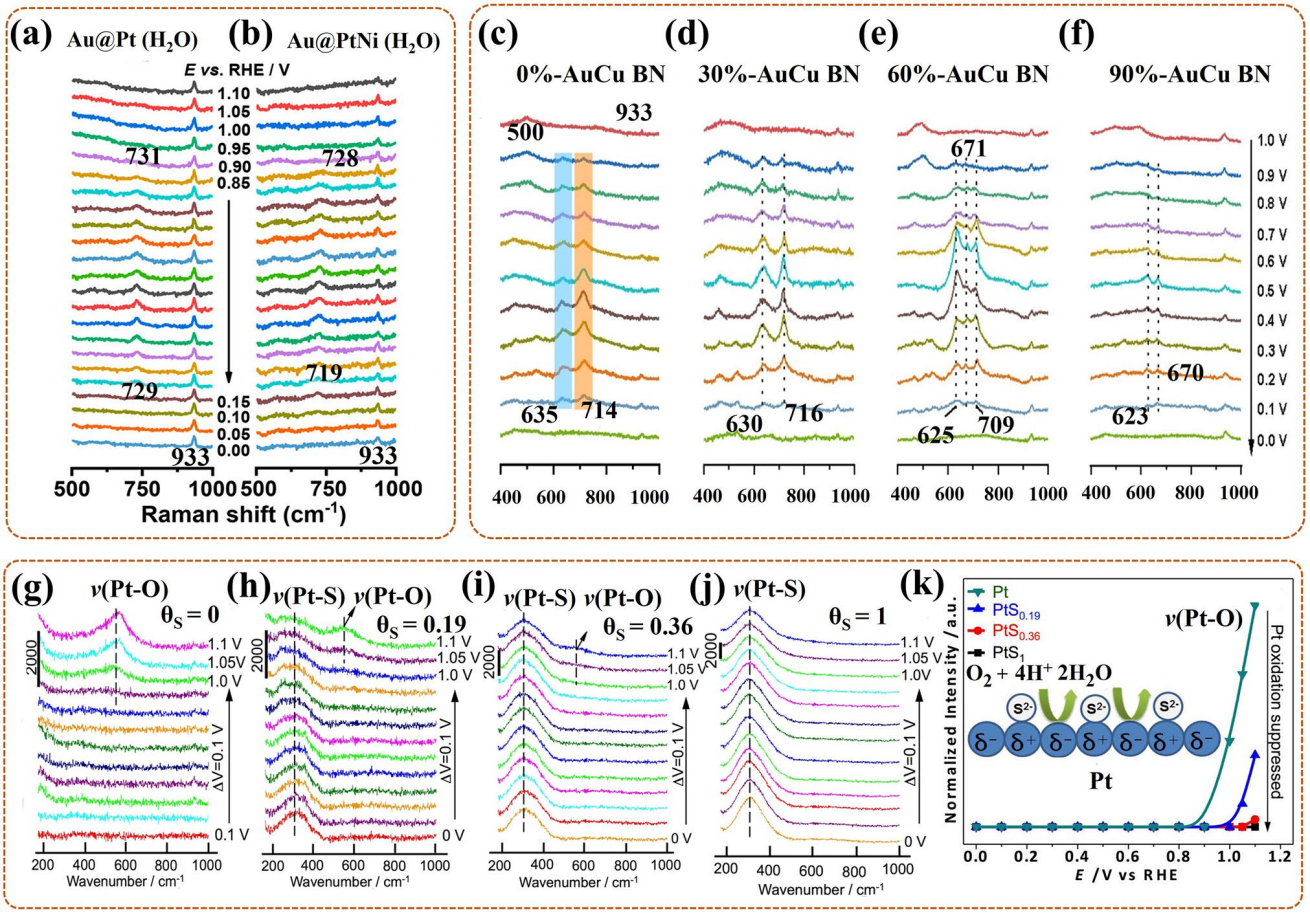
In-situ Raman spectra of **a** Au@Pt, **b** Au@PtNi NPs adsorbed by oxygenated species [130]. Reused with approval; Copyright 2021 American Chemical Society. In-situ Raman spectra of **c** 0%-AuCu, **d** 30%-AuCu, **e** 60%-AuCu, **f** 90%-AuCu BNs in 0.1 M NaClO_4_ + 0.1 mM NaOH with O_2_ saturated [131]; Reused with approval; Copyright 2022 Wiley-VCH GmbH. In-situ SERS spectra on the Pt surfaces with diverse sulfide coverages, **g** PtS_0_ (*θ*_S_ = 0), **h** PtS_0.19_ (*θ*_S_ = 0.19), **i** PtS_0.36_(*θ*_S_ = 0.36), **j** PtS_1_ (*θ*_S_ = 1); **k** The normalization of intensities of ν(Pt-O) band of PtS_0_, PtS_0.19_, PtS_0.36_, PtS_1_, with inset of the schematic of ORR on the Pt surface with sulfide-adsorbed [132]; Reused with approval; Copyright 2016 American Chemical Society

The dynamic adsorption/desorption behaviors of intermediates on the Pt alloy catalyst surface during ORR have also been widely reported. For instance, Ze et al. [130] used in-situ SERS to monitor the dynamic changes of intermediates on the Au@PtNix NP catalyst surface for revealing the nature of its catalytic performance optimization. For Au@Pt NP (Fig. 10a), there only existed a single peak (933 cm^−1^) at starting potential, which was ascribed to the ClO_4_^−^, and then, as the potential dropped to 0.85 V, another new peak caused by O–O vibration of *OOH appeared at 731 cm^−1^. For Au@PtNi NP with higher ORR activity (Fig. 10b), this new peak (associated with *OOH) appeared at a slightly lower position (728 cm^−1^), and occurred a clear shift toward 719 cm^−1^ at low potential (0.15 V). That was, the PtNi surface electron structure underwent changes, compared to pure Pt, also with a potential-dependent, further affecting the bond vibration of *OOH. Combining theories, the Ni doping promoted the generation of the binding bond among *OOH and Pt surface, also, the *OOH on PtNi with smaller adsorption energy than that on Pt, could facilitate the electron transfer, and consequently boosting the ORR activity. More, for Au@PtNix NP, as the Ni content increased, the *OOH occurred in more red-shift, due to the increase of O–O bond length, the decrease of bond energy, and the decrease of *OOH adsorption energy, ultimately more favorable for ORR. In conclusion, the adding of Ni could induce the electron structure changes of the Pt alloy catalyst, and independent with its content, further affecting the intermediates adsorption/desorption to improve the ORR activity. Thus, it proves that the intermediates adsorption/desorption behaviors can help to reveal the nature of the high activity of the catalyst, and this points to a certain direction for in-situ Raman detection.

Similar work has been done for bimetallic nano-catalysts (BNs), such as Chen et al. [131] used in-situ Raman to monitor the distinction of intermediates on the AuCu BNs surface with diverse ordering degrees for evaluating the effect of the ordering degree during ORR at the molecular level. In detail, for 0%-AuCu BN (completely disordered) (Fig. 10c), the *OH intermediate was proved to exist on its surface, corresponding to the Raman peak at 714 cm^−1^, with disordered Au–Cu as the active site. For 30%-AuCu BN (Fig. 10d), its Raman peak (716 cm^−1^) belonged to *OH was like to that of 0%-AuCu BN, indicating that its electrochemical behavior was similar as 0%-AuCu BN due to the lower atomic ordering. For 60%-AuCu BN (Fig. 10e), expect for Cu–O_ad_ (625 cm^−1^) specie and *OH (709 cm^−1^) intermediate, a new Raman peak emerged at 671 cm^−1^, due to the swing mode of *OH absorption on the ordered Au–Cu site. For 90%-AuCu BN (Fig. 10f), the 623 cm^−1^ peak and 670 cm^−1^ new peak still existed, while, the 710 cm^−1^ peak vanished, then, the new peak (670 cm^−1^) was further confirmed also due to the *OH absorption on the ordered Au–Cu site. Notably, after the disordered-to-ordered transition, the ORR activity of AuCu BN increased more than 2 times, and the one with highly ordered exhibited surpassed performance even than Pt/C. It indicated that both disordered and ordered Au–Cu could act as the sites for *OH adsorption, while the ordered Au–Cu site could make the catalysts with high ORR activity due to its ability to promote the desorption of *OH and its lower affinity for O_2_. Thus, not only the phase change detection can reveal the relationship between the BN order degree and its catalysis activity as discussed in Sect. 4.1, but also the dynamic change detection in intermediates also has the same effect based on this work. Further this extends our synthesis design basis and gives new ideas for the preparation of BNs precisely ordering degrees in favor of ORR activity.

Except for the adsorption of intermediates, external adsorbents also have a notable impact on the ORR activity of the catalyst, thus, the corresponding real-time characterization is an equal emphasis to reveal the mechanism. As an example, Wang et al. [132] utilized in-situ SERS to explore the ORR mechanism of the commercial Pt black with appropriate sulfide adsorption (*θ*_S_ = 0, 0.19, 0.36, 1). In-situ SERS spectra of Pt surface showed that (Fig. 10g–j), the ν(Pt–S) peak (300 cm^−1^) intensity was positively correlated with the sulfide content, however, the ν(Pt–O) peak (580 cm^−1^) intensity exhibited in negative correlate, and disappeared on the PtS_1_ (*θ*_S_ = 1) sample. Normalization of the integrated peak intensities of ν(Pt–O) for PtS_0_, PtS_0.19,_ and PtS_0.36_ showed that (Fig. 10k), the onset of surface Pt oxidation (SPO) shifted toward higher potentials of 0.9, 1.0, and 1.05 V, respectively. Both data demonstrated that the sulfide adsorption made SPO located in a low degree, namely, the Pt surface with more sulfide exhibiting more resistance to oxidation. The reasons were speculated as follows (Fig. 10k, inset), the residual available Pt sites had more negative chargers, caused by the sulfide adsorption, thereby with enhanced oxidation resistance. To the end, it proved that the moderate external adsorption (such as, sulfide, *θ*_S_ = 0.2–0.4) contributed to the residual available Pt sites in a better state, exhibiting better ORR activity and stability. Also, it points out a new idea for designing highly active catalyst, i.e., to focus not only on the adsorption of reaction intermediates, but also on the adsorption of external adsorbents.

As mentioned above, a variety of intermediates are proven to be formed during ORR, which then further adsorb on the active site, and finally precipitating from the catalyst surface after a corresponding reaction. In-situ monitoring their dynamic evolutions can directly reveal the ORR mechanism. In addition, their adsorption/desorption on the active center depend on the electronic structure of the catalyst, which has close relation to the active site. Thus, the detection of intermediates is help to identify the active site, which in turn guides the design of the efficient catalyst (main with four-electron pathway facilitation). Notably, the adsorption energy of them is a vital descriptor of ORR performance, and too weak adsorption can make the activation of the catalyst difficult, while too strong can cause poisoning, thus limiting the catalytic activity. Therefore, the developed catalyst should with suitable adsorption effect for intermediates. Many factors, such as dopant, high-valence metal center, special crystal plane, ordering, external adsorbent et.al, should be considered in the developing of the catalyst.

### Monitoring of the Evolutions for Products

For metal–O_2_/air battery, the complex pathways of ORR are closely related with the catalytic products in the catalytic process. The detecting of their evolution states is vital for indirectly revealing the active site of the catalyst, and directly identifying the ORR pathway. The different products are different phase, so in-situ XRD and Raman are important for tracing their evolution process. Except for in-situ monitoring of the phase transitions of the products formed on the catalysts surface, in-situ description of dynamic morphological evolutions of the charger/discharger products is also crucial to reveal the catalytic process. The following representative corresponding progress is reviewed in this part.

**Fig. 11:**
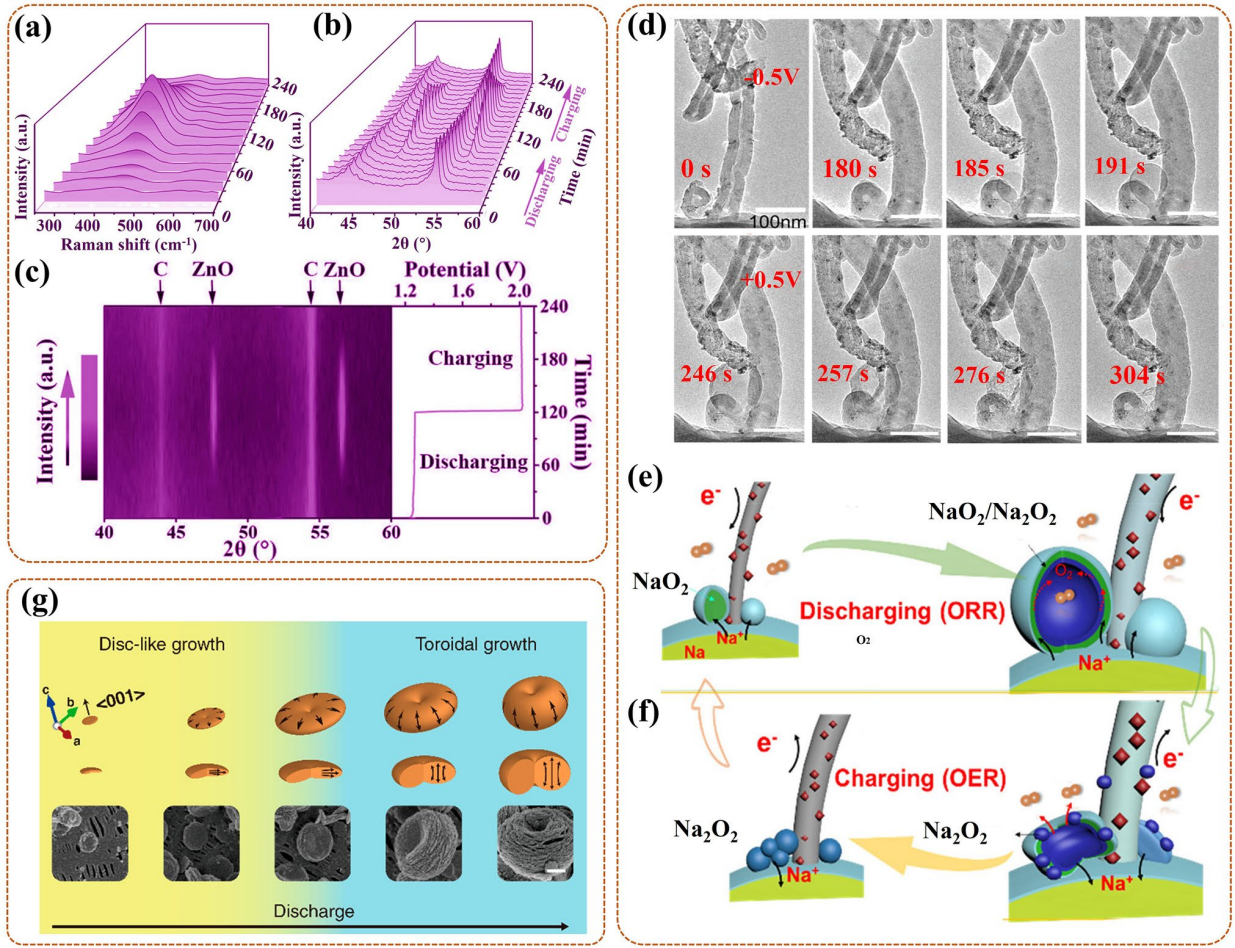
**a** In-situ Raman spectrum, **b** in-situ XRD pattern, **c** the in-situ XRD intensity map of Meso-CoNC@ GF during discharging (i.e., ORR) and charging in Zn–Air battery [81]; Reused with approval; Copyright 2017 WILEY-VCH Verlag GmbH & Co. KGaA, Weinheim. **d** In-situ TEM images of the product on the Pt_0.8_Ir_0.2_@CNT surface, at 0–191 s during discharge (ORR), and at 246–304 s during charging in Na-O_2_ battery, with schematics of** e** ORR process and **f** OER process [133]. Reused with approval; Copyright 2019 American Chemical Society. **g** Growth mechanism schematics of the product in the electrolyte (upper part), and the corresponding SEM image (bottom part), with DBBQ as catalyst, in the Li-O_2_ battery [134]. Reused with approval; Copyright 2019 American Chemical Society

For the detection of phase transitions, there exists a typical example. To ascertain active site of the heteroatom-doped carbon air electrode (i.e., Meso-CoNC@GF) during ORR and reveal the reversibility of Zn–Air battery, Liu et al. [81] used in-situ Raman and XRD to track the phase transitions of the products on its surface. The superior reversibility of ZnO product was visually displayed in-situ Raman spectrum. A peak attributed to ZnO gradually appeared at 413 cm^−1^ and enhanced, during discharge (i.e., ORR); in contrast, this trend was reversed for charging (Fig. 11a). The similar phase transitions were further confirmed in-situ XRD pattern, the peak of ZnO (JPCDS No. 36-1451) continuously enhanced during ORR and then disappeared after charging (Fig. 11b). Notably, two peaks belonging to carbon, at 44° and 54°, gradually became weaker during ORR (Fig. 11c); contrarily, there were no changes of the two peaks in the non-nitrogen doped electrode. This indicated that the carbon with nitrogen dopant, as the main active site, caused the electron deficiency, exhibiting a stronger catalytic effect for the reaction. In detail, the nitrogen with higher electronegativity doping in carbon could cause adjacent carbon atom to fall into electron deficiency, favoring the chemisorption of O_2_/oxygen-containing intermediates. The successive chemisorption gradually modulated the carbon, leading to the weakening of two carbon peaks. Finally, the two weak peaks could recover as before after charging, proving the reversible of the phase transforms on the specific active site. That is, in-situ results showed that Meso-CoNC@GF as the air electrode ensured the superior reversibility (Zn + O_2_ ↔ ZnO) of Zn–Air battery, also nitrogen-doped carbon was high activity for catalyzing ORR. Thus, this work proves that nitrogen-doped carbon can act as specific active site with highly catalytic activity in ORR, providing a basis for designing the Zn–Air battery catalyst with excellent reversibility.

For the detection of morphological transitions, including nucleation, growth and so on, the power tool is in-situ TEM. In addition to the application of in-situ TEM to monitor the morphological transitions of the catalysts in their synthesis process (as discussed in Sect. 4.3), many studies have also focused on its application of charger/discharger products generated on the catalysts surface in the catalytic reaction process. This process can reversely determine the optimal structure of the catalyst, which can promote products generation and reversible evolution. Zhu et al. [133] applied in-situ aberration-corrected TEM with a rational environmental cell to promulgate morphological evolutions of the products during ORR catalyzed by the bimetallic Pt_0.8_Ir_0.2_-doped carbon nanotube (Pt_0.8_Ir_0.2_@CNT) in a Na–O_2_ battery. During start discharge, Na^+^ rapidly diffused to Pt_0.8_Ir_0.2_@CNT driven by a potential of − 0.5 V, resulting in volume swell (from 50 to 80 nm) (Fig. 11d, 0–180 s). After the entire intercalation of Na^+^ at 180 s, a hollow sphere gradually formed on the catalyst surface (Fig. 11d, 185 s). Subsequently, this sphere continuously grew (from 30 to 120 nm), also with a raising in the shell thickness (from 4 to final 15 nm, at 246 s) (Fig. 11d, 185–246 s). Notably, during the process (i.e., ORR), Na^+^ firstly reacted with O_2_ at the Pt_0.8_Ir_0.2_ site on the CNT surface to form the NaO_2_ phase, followed by ceaselessly amplifying to form a larger sphere, and further breaking down to Na_2_O_2_ sphere with hollow structure (Fig. 11e). During charging, the special sphere quickly shrank and gradually disappeared driven by a potential of + 0.5 V at 257–304 s (Fig. 11d). This was due to the deintercalation of Na^+^ and the escape of O_2_ leading to the complete decomposition of NaO_2_ and Na_2_O_2_ (Fig. 11f). That was the Pt_0.8_Ir_0.2_@CNT catalyst with Pt_0.8_Ir_0.2_ as activity site could efficiently facilitate product generation and reversible evolution. This work has realized the visual monitoring of the dynamic changes of catalyst surface morphology during ORR. Further, it provides visual evidence for identifying the product, and guides the structural design of the highly active catalyst (such as with reasonable Pt/Ir ratio) in reverse.

More importantly, a serious problem for metal–O_2_/air batteries is that the O_2_/air electrode is easy to occur passivation, induced by the property of the formed discharge products. More researches focus on the redox mediator, based on its unique characteristic of transferring charge from electrode to electrolyte. To develop available redox mediator, in-situ tracking the morphological evolutions of their products can help to provide rational guidance. For example, Lee et al. [134] used in-situ TEM also with liquid cell to pursue the growth morphology changes of Li_2_O_2_ discharge product in the electrolyte with the catalysis of redox media (2,5-di-tert-butyl-1,4-benzoquinone (DBBQ)) during discharge (i.e., ORR) of the Li–O_2_ battery. In detail, the Li_2_O_2_ product firstly grew laterally to form a disk shape, and then grew along the vertical direction, especially with high speed in periphery edge, eventually, forming a toroidal shape (Fig. 11g). The growth rate was related to the distance between Li_2_O_2_ and the cathode, during which the DBBQ facilitated the transferring of charge from electrode to solution. The results proved that DBBQ could serve as one kind of redox mediator for promoting the growth of the Li_2_O_2_ product in the electrolyte not in the electrode during ORR, further avoiding the electrode passivation. This work can act as the evidence for in-situ visualization of the ORR process in liquid system, and point out the ORR mechanism going along the solution growth mechanism. Also, it proved that the charge transform ability (from electrode to solution) of the catalyst is essential for efficient ORR, which is basic for the design of the ORR catalyst for the metal–O_2_/air battery.

Another serious problem is that the crucial factors with effect on morphological evolutions of products for metal–O_2_/air batteries are mostly unknown. Combining in-situ electron diffraction and imaging to identify the factors is necessary. In the works of Han et al. [135], the dynamic evolutions of products in the ORR and OER processes, with Cu-based catalyst on the cathode, were observed by in-situ TEM. During ORR, NaO_2_ formed at the early stage, and then disproportionated to mesoporous particle-shaped Na_2_O_2_ (with orthorhombic/hexagonal structure), eventually as the main product equably covering on the whole cathode. During OER, the Na_2_O_2_ reversely converted to NaO_2,_ accompany by volume expansion, and the latter further reversibly decomposed to Na^+^ and O_2_. Notably, in-situ electron diffraction revealed that the reversible cycle depended on Cu nanocluster formed by in-situ solidified CuS. It proves that the size, composition, and distribution information of Cu nanocluster are essential for the modulation of the morphology, component, and size of product in the reaction process. In summary, this can act as another pivotal evidence for the rational design of the catalyst. It is necessary to regulate the indicators mentioned above affecting its structure to promote the reversibility of a Na-O_2_ battery.

**Fig. 12:**
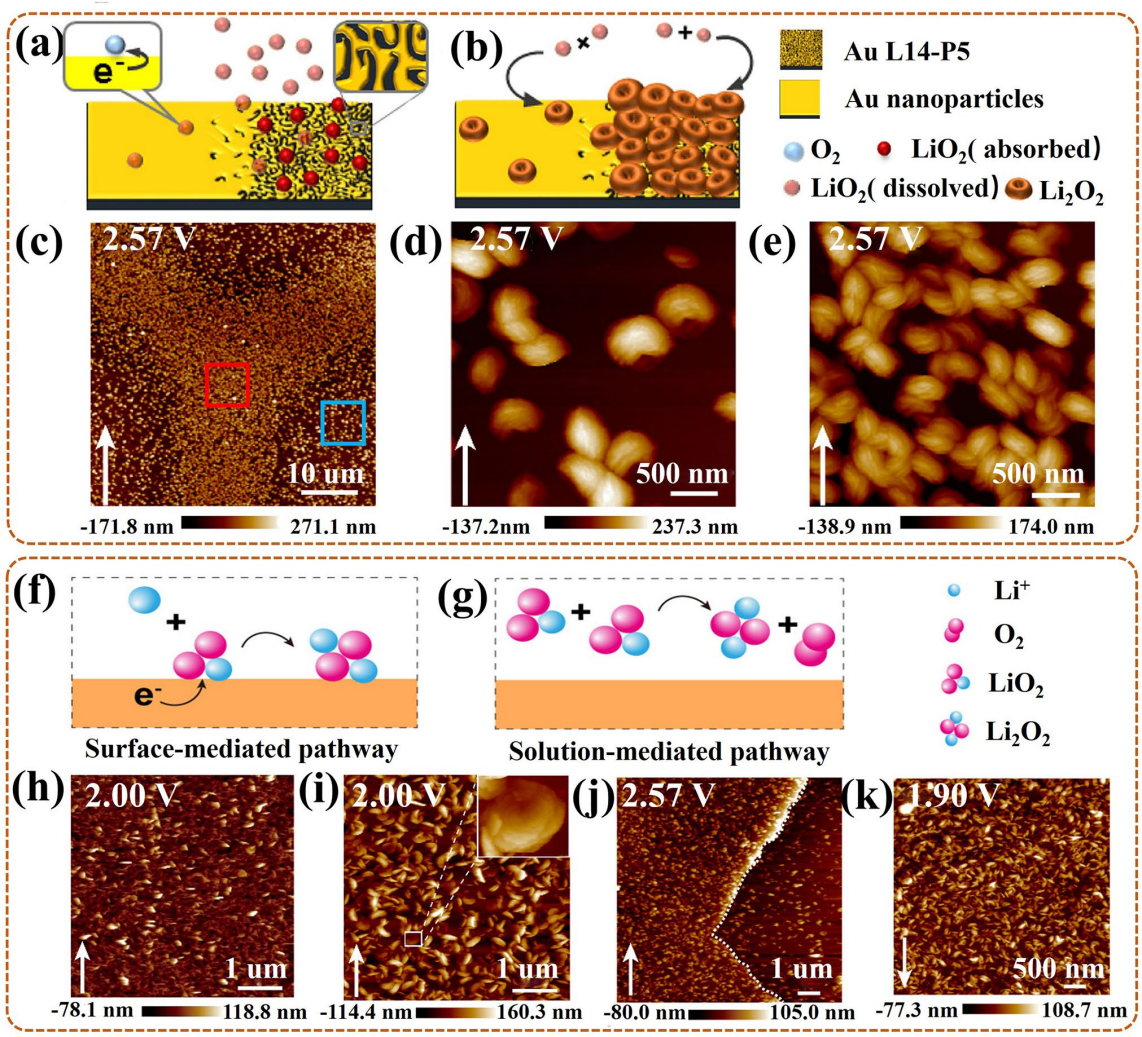
Schematic of ORR processes on the Au NPs/Au L14-P5 electrode, **a** at early stage, **b** at end stage; In-situ AFM images of the composite electrode, **c** with the large-scale Li_2_O_2_ deposition, **d** the ring Li_2_O_2_ on Au NPs region (the enlarged blue frame of **c**), **e** the ring Li_2_O_2_ on Au L14-P5 region (the enlarged red frame of **c**), with white arrow as scanning direction [97]; Reused with approval; Copyright 2020 American Chemical Society. Schematic of ORR processes reaction paths on, **f** Pt-0 electrode, **g** Pt-80 electrode; In-situ AFM images of **h** Pt-0 electrode, **i** Pt-80 electrode, **j** Pt-250 electrode, **k** Pt/Au NPs electrode, with white arrow as scanning direction [98]. Reused with approval; Copyright 2021 American Chemical Society. (Color figure online)

In addition, in-situ AFM is also a powerful tool for the monitoring of morphological evolutions of the products during ORR in metal–O_2_/air battery, especially for interface. That is, it is another useful way to clarify the pivotal effect factors guiding the design of catalyst structures and to unravel the ORR mechanism. Shen et al. [97] proceeded this monitoring on the three-phase interface (Au electrode/electrolyte/O_2_) during ORR in the Li–O_2_ battery by in-situ AFM. In detail, the Au nanoparticles (NPs) and Au L14-P5 (with pore size ~ 5 nm and ligament width ~ 14 nm) were used as electrodes, which were prepared by sputtering Au onto highly oriented pyrolytic graphite (HOPG), also with Teflon tape as the mask, at different sputtering time. Then, in-situ AFM of Au NPs/Au L14-P5 composite electrode (Fig. 12c) showed that the products, ring Li_2_O_2_ (diameter ~ 500 nm, thickness ~ 150 nm), mainly concentrated on Au L14-P5 (Fig. 12e), and only little distributed on Au NPs (Fig. 12d) during ORR. That indicated that Au L14-P5 had a stronger ORR catalytic activity. This was due to the higher nucleation potential of Li_2_O_2_ on Au L14-P5 (2.61 V) than on Au NPs (2.54 V), making the prioritized nucleation and growth of Li_2_O_2_ on Au L14-P5 (Fig. 12a). Even in the end of ORR (Fig. 12b), large-sized Li_2_O_2_ products were also primarily distributed on AuL14-P5, and there were little products on the Au NPs area. This further proved that Au L14-P5 had a more rational structure for higher ORR catalytic. This work shows that the nanostructure of Au determines the catalytic behavior of its surface, and the suitable size and nano-porous are the important factors in the design of the catalyst structure. The observed ring Li_2_O_2_ products on the composite electrode point out the ORR mechanism going along the electrode growth mechanism.

Afterwards, they used in-situ AFM again to proceed with similar monitoring on the Pt nanoparticles (NPs) electrode during ORR cycle, also in the Li–O_2_ battery [98]. In first oxidation reduction cycle (ORC), the original electrode (Pt-0) with 5 nm Pt NPs boosted ORR mainly through the surface path (LiO_2_ + Li^+^  + *e*^−^ → Li_2_O_2_) (Fig. 12f). Also, the O_2_ reduction peak of Pt-0 was at 2.51 V. And in-situ AFM showed that many products NPs (thickness ~ 10 nm) firstly deposited on Pt-0 surface, then converted to nanosheets (Li_2_O_2_) and further grew (finally with thickness to 20 nm) to cover on its surface (Fig. 12h). During 0–80 ORCs, the Pt-80 electrode with 10 nm Pt NPs boosted the ORR mainly through the solution-mediated route (2LiO_2_ → Li_2_O_2_ + O_2_) (Fig. 12g). Compared to Pt-0, the O_2_ reduction peak of Pt-80 occurred positive shift (from 2.51 to 2.58 V), also with an increased peak intensity. And in-situ AFM showed that many products NPs (diameter ~ 10 nm) firstly deposited on Pt-80 surface, then quickly grew into new structures with disk-shaped, with further increase in thickness and diameter, and finally grew into the ring-like Li_2_O_2_ (diameter ~ 300 nm) (Fig. 12i). After 250 ORCs, the Pt-250 electrode with overgrown Pt NPs exhibited seriously decreased catalytic activity, also, with smaller and less Li_2_O_2_ on its surface (Fig. 12j). Notably, the modified Pt/Au electrode, namely, with adding Au NPs on Pt NPs, could exhibit high ORR activity like Pt-80, also with ring-like Li_2_O_2_ (diameter ~ 200 nm) as the final products (Fig. 12k). In conclusion, it proved that the Pt NP diameter could grow with the ORCs, and the Pt NP with the suitable diameter could ensure high ORR activity, promoting the reaction along a solution-mediated route. Then the excessive growth of the Pt NP could reduce the catalytic activity, and the electrode modification was useful to maintain high catalytic activity. This work further strengthens our awareness to use in-situ AFM to detect the evolution state of the products and thus to reveal the ORR mechanism. And it provides a powerful basis for revealing the ORR mechanism and in-depth clarifying the effect factors for the enhanced catalytic activity during ORCs. Also, a suitable size or modification structure is essential for developing high-activity catalyst.

In summary, the different products have different phases and different morphologies, so in-situ Raman/XRD and in-situ TEM/AFM are equally important for tracing their evolution process to clarify the ORR mechanism. These can reveal that some catalyst indicators, such as doping, metal ratio, charge transform, size, composition, distribution, porosity, and modification, can affect the ORR activity, further guiding the catalyst design.

### Monitoring of Electrolyte Interfacial Anion Chemisorption

The adsorption of anions in the electrolyte on the catalyst surface can trigger its poisoning and make the ORR process become more complex. It is difficult to track catalyst contamination with traditional technologies, making poisoning problem difficult to solve and making the elucidation of the mechanism difficult. It is essential to probe in-situ techniques to check the dynamics of anion adsorption/desorption. It can offer useful information for revealing the ORR mechanism and guiding the development of catalysts to inhibit anion adsorption without affecting catalytic performance.

**Fig. 13:**
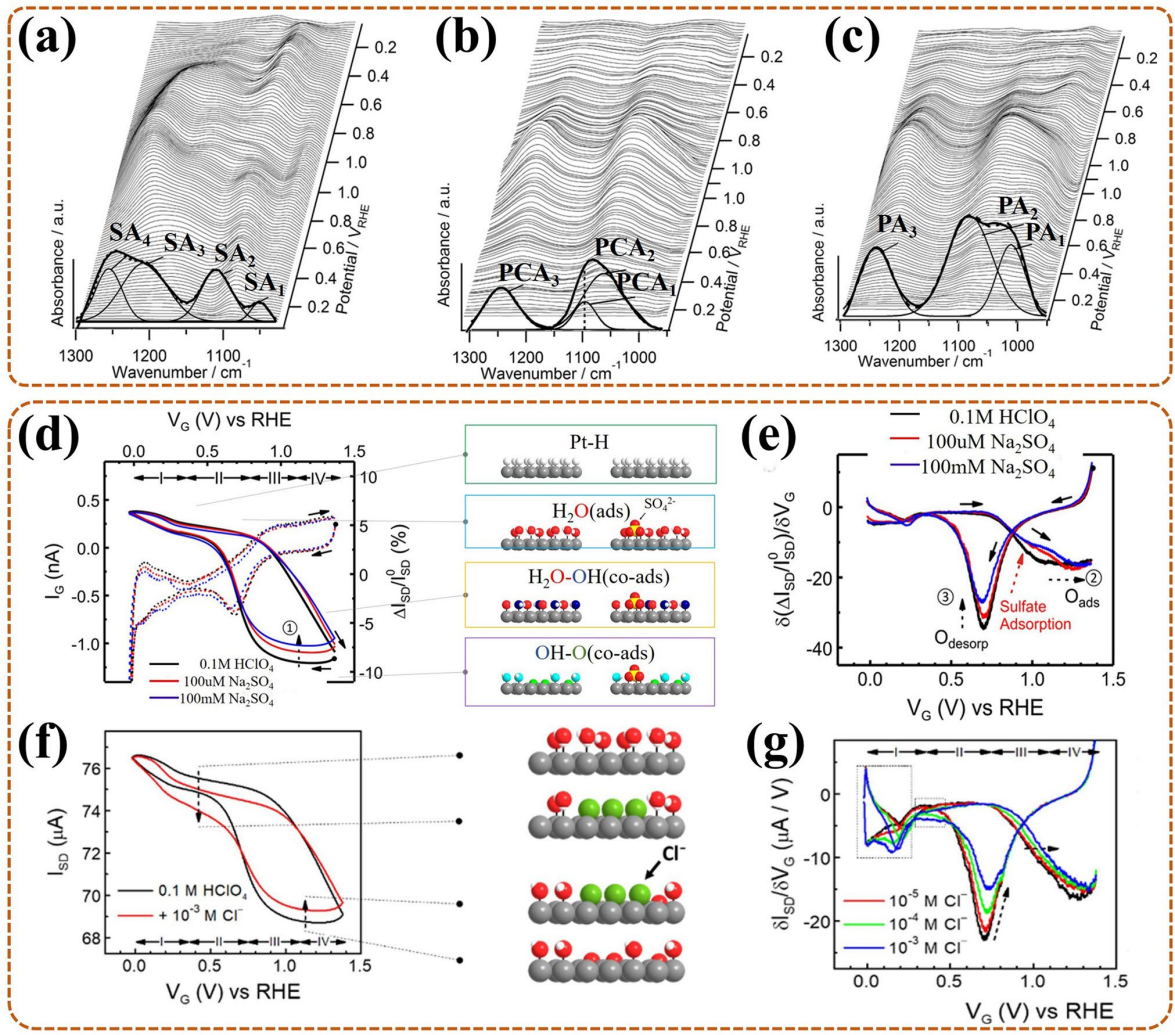
In-situ ATR-FTIR waterfall plots on Pt/C during ORR and with corresponding deconvolution by Gaussian fitting at 0.07 V, **a** in 0.5 M H_2_SO_4_ and for (bi)sulfate adsorption, **b** in 0.5 M HClO_4_ and for ClO_4_ adsorption, **c** in 0.5 M HClO_4_ + 10 mM H_3_PO_4_ and for relative adsorption [136]; Reused with approval; Copyright 2016 WILEY–VCH Verbg GmbH&Co. KGaA, Weinheim. **d** In-situ ETS curves (solid lines) and cyclic voltammetry curves (dash lines, without explain in this paper) of sulfate adsorptions on PtNW surface, with schematic of surface adsorption models in right side, with Pt (gray), H (white), O (red, in H_2_O_ads_; blue, in OH_ads_), **e** dETS curves in 0.1 M HClO_4_ and 100 uM/100 Mm NaSO_4_; **f** In-situ ETS curves of chlorides adsorption, also with modes schematic in right side, with Pt (gray), H (white), O (red), Cl (green); **g** dETS curves with changing chloride concentrations; All dashed arrows for the influence of sulfates/chlorides adsorption on the varies of ETS or dETS [103]. Reused with approval; Copyright 2018 American Chemical Society. (Color figure online)

For the detection of common anions, the technique of in-situ IR is widely used. For instance, Nesselberger et al. [136] observed some anion adsorption behaviors on Pt/C catalyst during ORR in PEMFC in real time utilizing in-situ ATR-FTIR. In 0.5 M H_2_SO_4_ (Fig. 13a), in-situ ATR-FTIR showed there could be fitted four bands for Pt/C catalyst, with bare C as a comparison. The SA_1_ (1045 cm^−1^) emerged on Pt/C and bare C, with less intensity, thus assigned to a bisulfate vibration mode [137]. For SA_3_ (1180 cm^−1^) or SA_4_ (1235 cm^−1^), both with increased intensities on Pt/C, thus belonged to the mutual effect of (bi)sulfate with carbon, or the superposed effect of (bi)sulfate with Pt and C–O stretching, respectively. In 0.5 M HClO_4_ (Fig. 13b), in-situ ATR-FTIR showed there firstly existed fitted three bonds, followed by transformed to two peaks. The PCA_1_ (1050 cm^−1^) or PCA_3_ (1250 cm^−1^) were assigned to the mutual effect of perchloric acid with carbon, or with C-O stretching, respectively. For PCA_2_ (1095 cm^−1^), attributed to the Cl-O vibration style of the perchlorate anion, with notable varieties on the Pt/C, and ultimately overlapped with PCA_1_ and vanished. In 10 mM H_3_PO_4_ + 0.5 M HClO_4_ (Fig. 13c), in-situ ATR-FTIR showed a new PA_1_ (1000 cm^−1^) emerged, while PA_2_ (1075 cm^−1^) and PA_3_ (1235 cm^−1^) remained in line with PCA_1_ and PCA_3_. The PA_1_ band (H_2_PO_4_^−^ mode) belonged to the mutual effect of phosphoric acid with Pt/C, and was transformed from the H_3_PO_4_ absorbed on Pt (induced by C_3v_ symmetry, 1040 cm^−1^). In summary, the fitted bands in different electrolyte could act as the special functions to depict the adsorption behaviors of different anions on Pt. This work provides the IR information about these common solution anions, also as a basis for characterizing the effect of anion adsorptions on ORR mechanism.

However, for some anions without obvious IR signal, such as halides, in-situ IR is unapplicable. In addition, to ensure in-situ qualitative and quantitative characterization of the anion adsorptions on the catalyst surface at a low level, the in-situ ETS has been used in special catalyst to monitor ORR process in fuel cell. For example, Ding et al. [103] detected dynamic changes of anion adsorptions (sulfates, chlorides etc.) and surface oxidation on ultra-thin Pt nanowire (Pt NW) by in-situ ETS and correlated them with the kinetics of ORR. For sulfates (Fig. 13d), at negative potential region, the ETS current increased/decreased notably, with the adsorption/desorption of monolayer H atom (H_ads/desop_) (green frame). At double-layer (D.L.) region, the ETS current remained flat, with H_2_O as the main adsorbed specie (blue frame). At positive potential region, the ETS current also changed notably, indicating the existence of reversible hydroxyl group (OH_ads_) adsorption (orange frame) and surface oxidation (purple frame). The differential ETS (dETS) (Fig. 13e) showed that both O_ads_ and O_desop_ (dashed arrow 2 and 3) decreased as sulfate content increased; also, OH_ads_ region shifted notably (red dashed arrow), while surface oxidation region remained unchanged. It meant that the sulfates adsorption on the Pt surface occurred by co-adsorption with hydroxyl groups. For chlorides (Fig. 13f), at D.L. region, the ETS current decreased notably (downward dashed arrow) due to the Cl_ads_ adsorption in the inner Helmholtz plane (IHP); at high potential region, it increased slightly (upward dashed arrow) owing to the slight surface oxidation. The dETS (Fig. 13g) showed that both O_ads_ and O_desop_ decreased as chloride content increased, owing to the decrease in O_ads_ adsorption sites blocked by Cl_ads_; also, O_ads_ peak shifted notably, suggesting that the overpotential of O adsorption increased due to the blocking of Cl_ads_. It meant that the chlorides adsorption on the Pt surface changed the kinetics of the intermediate ORR steps, resulting in Pt poisoning. These results suggested that the complex interaction between anions adsorption and oxide formation was the key factor for causing site blocking and then affecting ORR kinetics. This work provides the ETS information about the special solution anions without IR signal, such as chlorides. It indicates that in-situ ETS is beneficial to assist in-situ IR to reveal actual changes in the ORR process, thereby facilitating the elucidation of the ORR mechanism. In addition, it is essential to in-situ monitor both anions adsorption and surface oxidation information during ORR to guide the design of catalysts with ability to resist corrosion and oxidation.

The common SO_4_^2−^ adsorption on the Pt surface is more severe, while the interaction between ClO_4_^−^ and Pt is weaker. In-situ IR can act as the key monitor technique for these anions (with IR signal). In addition, more toxic to Pt surface is Cl^−^, which often has complex interactions with the formed oxides, blocking the catalytic site and affecting ORR kinetics. Due to without IR signal, it monitoring needs to be used in-situ ETS. Thus, for Pt-based catalyst, ORR testing is preferably performed in HClO_4_ solution. Comminated with in-situ techniques, anion adsorption on any modified catalyst can be traced to visualize the adsorption information and identify the soundness of the catalyst structure design. Further, based on the obtained information, it can light the structure design direction of the corrosion-resistant and anti-toxicant catalyst, and help to elucidate the ORR mechanism.

According to the above discussion, it can be further proved that each technique has its uniqueness for the monitoring of various species during ORR. The detection of dynamic changes of intermediates, products, and anions with different in-situ techniques is summarized in Table 2. Prominently, the evolution of intermediates, dominated by molecular vibrational changes, can be specifically provided by in-situ IR/Raman. The evolution of products is more prominent with morphological changes, and the application of in-situ techniques with morphological detection functions is more prominent. The solution anions signals can be offered by in-situ IR/Raman or in-situ ETS (especially for halides). Considering that not only one species is included in the complete ORR process, it is crucial to combine several techniques for obtaining the rich information on the various species, further guiding the revelation of the mechanism. For example, except for the intermediates, products and anions information, the coordination/bonding, spin state information detected by in-situ XAS and Mössbauer spectra (as discussed in Sect. 4) are also important. More detected species on some represent electrocatalysts are summarized in Table 3 by using various in-situ techniques.

**Table 3:** Detected species on some represent electrocatalysts using various in-situ techniques

Catalysts	Detected species	Conditions	Techniques	References
O-PtFe	XANES, metallic state Pt^0^; EXAFS, Pt–Pt, 2.708 Å < pure Pt, 2.775 Å, Fe CN, 2.6, Pt CN, 7	PEMFC, O_2_, 10 k ADT, 0.1 M HClO_4_	In-situ XAFS	[112]
Int-PtCuN/KB	XANES, less Cu oxidation, Pt oxidation; EXAFS, weak Cu–O, 1.5 Å, Pt–O, 1.6 Å, short Pt–Pt bond, 2.693 Å	PEMFC, O_2_, 0.1 M HClO_4_	In-situ XAS	[113]
Cu/Zn-NC	Zn–N, no changes in XANES and EXAFS; Cu–N, ~ 1.5 Å, weakened, CN, 4 → 2, Cu–Cu, ~ 2.2 Å, formed, at 0.4–0.3 V, Cu–Cu, disappeared at end. (EXAFS)	PEMFC, O_2_, 0.1 M KOH	In-situ XAS	[115]
Mn-SAS/CN	OH_ads_–Mn^H+^-N_4_ ↔ Mn^L+^-N_4_ + OH^−^ XANES, edge and white line → higher energy → initial state	Zn–Air battery, O_2_, 0.1 M KOH	In-situ XAS	[116]
Cu–N-C SAC	Cu^2+^–N_4_ → Cu^+^–N_3_, four Cu–N broken, one pyridinic N was protonated, three elongated by 0.04, 0.03, 0.13 Å; HO-Cu^+^-N_2_, two Cu–N, 1.985, 1.918 Å	PEMFC, O_2_, 0.1 M KOH	In-situ XAS	[138]
Pt/C	Bisulfate, 1045 cm^−1^; (bi)sulfate, 1100 cm^−1^; (bi)sulfate with carbon, 1180 cm^−1^; (bi)sulfate with Pt and C–O, 1235 cm^−1^	PEMFC O_2_, 0.5 M H_2_SO_4_	In-situ ATR-FTIR	[136]
	ClO_4,_ 1095 cm^−1;^ ClO_4_ with C–O, 1250 cm^−1^; ClO_4_ with carbon, 1050 cm^−1^	0.5 M HClO_4_		
	H_2_PO_4_^−^, 1000 cm^−1^; ClO_4,_ 1095 cm^−1^	10 mM H_3_PO_4_ + 0.5 M HClO_4_		
	ClO_4_ with C–O, 1250 cm^−1^			
Pt NWs	OH_ads_ and sulfate (HSO_4_^−^, SO_4_^2−^-H_3_O^+^, SO_4_^2−^) co-adsorption O_ads_, O_desop_ decreased as Cl_ads_ increased	100 μM/100 Mm NaSO_4_ 0.1 M HClO_4_ + 10^–3/−4/−5^ mM Cl^−^	In-situ ETS	[103]
Pt/C	O_2, ad_, 1468 cm^−1^, HOOH_ad_, 1386 ± 4 cm^−1^, OOH_ad_, 1212 ± 3 cm^−1^; ClO_4_, 1114 cm^−1^, ClO_3_, 1030 cm^−1^, C functional groups, 1435 cm^−1^, 1330 cm^−1^Si–O–Si, 1260 cm^−1^	Fuel cell, O_2_, 0.1 M HClO_4_	In-situ IR	[93]
N-doped carbon	O_2, ads_, 1468 cm^−1^, OOH*, 1019 cm^−1^, O_2_^−^*, 1052 cm^−1^	0.1 M KOH	In-situ ATR-IR	[126]
NiFe-MOF	*OOH, 1048 cm^−1^	0.1 M KOH	In-situ FT-IR	[127]
Co-SAC	OOH_ad_, 1224 cm^−1^ (main), HOOH_ad_, 1030 cm^−1^ (weak)	0.1 M HClO_4_	In-situ ATR-IR	[128]
Pt single crystal	Pt (111), HO_2_*, 732 cm^−1^;	Fuel cell, O_2_, 0.1 M HClO_4_ 0.1 M NaClO_4_	In-situ Raman	[89]
	Pt (110), (100), OH*, 1080 cm^−1;^ Pt (111), (110), (100), O_2_^−^, 1150 cm^−1^			
Pt single crystal	Pt (311), OH, 1041 cm^−1^; OOH, 766 cm^−1^; Pt (211), OH, 1042 cm^−1^; OOH, 765 cm^−1^; ClO_4_^−^, 933 cm^−1^	0.1 M HClO_4_	In-situ Raman	[129]
Au@PtNix NPs	*OOH, 728 cm^−1^ → 719 cm^−1^ (0.15 V); Au@Pt NPs, *OOH, 731 cm^−1^; ClO_4_^−^, 933 cm^−1^	0.1 M HClO_4_	In-situ Raman	[130]
AuCu BN	0%- AuCu BN, *OH, 714 cm^−1^; 30%-AuCu BN, *OH, 716 cm^−1^; 60%-AuCu BN, *OH, 709 cm^−1^/671 cm^−1^, Cu–Oad 625 cm−1; 90%-AuCu BN, *OH, 670 cm^−1^, Cu–O_ad_ 623 cm^−1^	M NaClO_4_ + 0.1 mM NaOH	In-situ Raman	[131]
Pt black	Pt–S, 300 cm^−1^, Pt–O, 580 cm^−1^	0.1 M HClO_4_	In-situ Raman	[132]
FeEs_4_	LS Fe^III^–OOH, 830 and 631 cm^−1^; Fe^IV^=O, 782 cm^−1^; HS Fe^II^–OH_2_, 1352 and 1540 cm^−1^; LS Fe^III^–OOH, 1369 and 1565 cm^−1^; Fe^IV^=O, 1371 and 1571 cm^−1^; HS Fe^III^–OH, 1364 and 1555 cm^−1^	pH 7 buffers	In-situ Raman	[139]
Pt_3_Co NP	*OOH, 697 cm^−1^, O_2_*, 860 cm^−1^; ClO_4_^−^, 933 cm^−1^, Pt-O, 558 cm^−1^	Fuel cell, O_2_, 0.1 M HClO_4_	In-situ Raman	[140]
	*OOH, 698 cm^−1^, *OH, 750 cm^−1^; Pt-O, 557 cm^−1^	0.1 M NaClO_4_ + 0.1 mM NaOH		
FePc/C	Fe-N_4_, 593 cm^−1^; C/N, 685, and 754 cm^−1^	PEMFC, O_2_, M HClO_4_ 0.1 M KOH	In-situ Raman	[141]
	O_2_^−^, 1100–1200 cm^−1^ band peak			
Fe–NC–S (Six N-coordination)	Fe^2+^ HS → LS, on N–Fe^II^N_4_C^10^ (D3), form O_2_^−^–Fe^II^N_5_, at 0.9 V, QS, 2.60 → 0.97 mm s^−1^, IS, 0.77 → 0.39 mm s^−1^; Fe^2+^ LS → HS, on Fe^II^N_4_C_12_ (D1), form O_2_^−^–Fe^II^N_4_, at 0.7 V, QS, 0.80 → 2.46 mm s^−1^, IS, 0.37 → 0.77 mm s^−1^; O_2_^−^–Fe^II^N_4_ with large content at 0.5 V	PEMFC, O_2_, M KOH, at room temperature	In-situ Mössbauer spectra	[109]
Fe–N-C	HS FeN_4_C_12_ (D1/S1), QS, 0.9–1.2 mm s^−1^; LS/MS FeN_4_C_10_ (D2/S2), QS, 1.8–2.8 mm s^−1^	PEMFC, Ar, in acidic medium, 50 °C	In-situ Mössbauer spectra	[124]
S-doped Fe1-NC (For Fe_1_-3 NS_1.3_C)	LS Fe^3+^ in X-Fe^3+^N_4_-Y (D1), QS, 1.649 mm s^−1^, IS, 0.111 mm s^−1^; HS Fe^2+^ in Fe^2+^N_4_-Y (D2), QS, 1.92 mm s^−1^, IS, 0.92 mm s^−1^; HS Fe^2+^ in X-Fe^2+^N_4_-Y (D3), QS, 2.75 mm s^−1^, IS, 1.22 mm s^−1^	PEMFC, O_2_, M KOH, at room temperature	In-situ Mössbauer spectra	[125]
FeNC	HS Fe^II^N^4^ (D3) decreased, 0.9 V → 0.75 V, δ_iso_, 1.03 mm s^−1^, ∆*E*_Q_, 1.55 mm s ^−1^; Held steady, 0.6 V → 0.2 V, δ_iso_, 0.91 mm s^−1^, ∆E_Q_, 2.2 mm s ^−1^, then vanished, 0.9 V	PEMFC, N_2_ 0.1 M H_2_SO_4_	In-situ Mössbauer spectra	[142]
Fe–N–C (FeN_4_ site)	LS FeN_4_C_8_, HS FeN_4_C_12_ (D1), LS/MS FeN_4_C_10_ (D2), FeN_4_ (D4), QS, ~ 1.3 mm s^−1^, ~ 2.9 mm s^−1^, ~ 0.8 mm s^−1^, IS, 0.31–0.39 mm s^−1^; N–FeN_2+2_…N_prot_/C (D3), high QS, IS	PEMFC, O_2_, 0.1 M HClO_4_, at 298 K	In-situ Mössbauer spectra	[143]

### Inferring Reaction Mechanism by Associating In-Situ Studies with Theoretical Calculations

Different from conventional techniques, in-situ techniques can supply expedient experimental guidance for the ORR of catalysts by checking the dynamic evolution of intermediates, corresponding catalytic products, electrolyte anions, and external adsorbents, etc. in real time. In addition, in conjunction with theoretical computational techniques, the construction of theoretical models for imitating the catalytic process is beneficial for further in-depth investigation of the elements impacting ORR performance from the kinetic direction at the atomic standard. This is essential to reveal the ORR mechanism and the relevant works are summarized below.

Ab initio FEFF8 calculation, as a typical auxiliary tool for XAS, is always used to combine with in-situ XAS during ORR to reveal the electronic structure changes of the catalyst and carry out the reason for its enhanced activity. For example, to inquire into the causality of the higher activity for de-alloyed Pt_1_Co_1_ NP catalyst during catalyzed process, Jia et al. [144] merged in-situ XAS, ex-situ HAADF, with FEFF8 to identify the changes during ORR in PEMFCs, also in comparison with pure Pt catalyst. The experimental results showed that the de-alloyed Pt_1_Co_1_ NP had a peculiar core–shell structure, with the ordered Pt_1_Co_1_ as the core, and the ultrathin and nonporous Pt skin as the shell. Combined with FEFF8 to reveal the effect of the electronic properties, it showed that the d-band center (*ε*_d_) of Pt_19_Co_6_ (111) or Pt_10_Co_4_ (100) with the presence of Co was lower than Pt_25_ (111) or Pt_14_ (100), respectively. In conclusion, the higher catalyst activity of de-alloyed Pt_1_Co_1_ NP for ORR was due to the lower ε_d_ induced by both ligand effects and compressive-strain, which was initially triggered by the Co enriched in the ordered Pt_1_Co_1_core and near the subsurface. It proves that FEFF8 plays an important role to reveal the relationship of the catalytic activity and electronic structure changes of the catalysts.

**Fig. 14:**
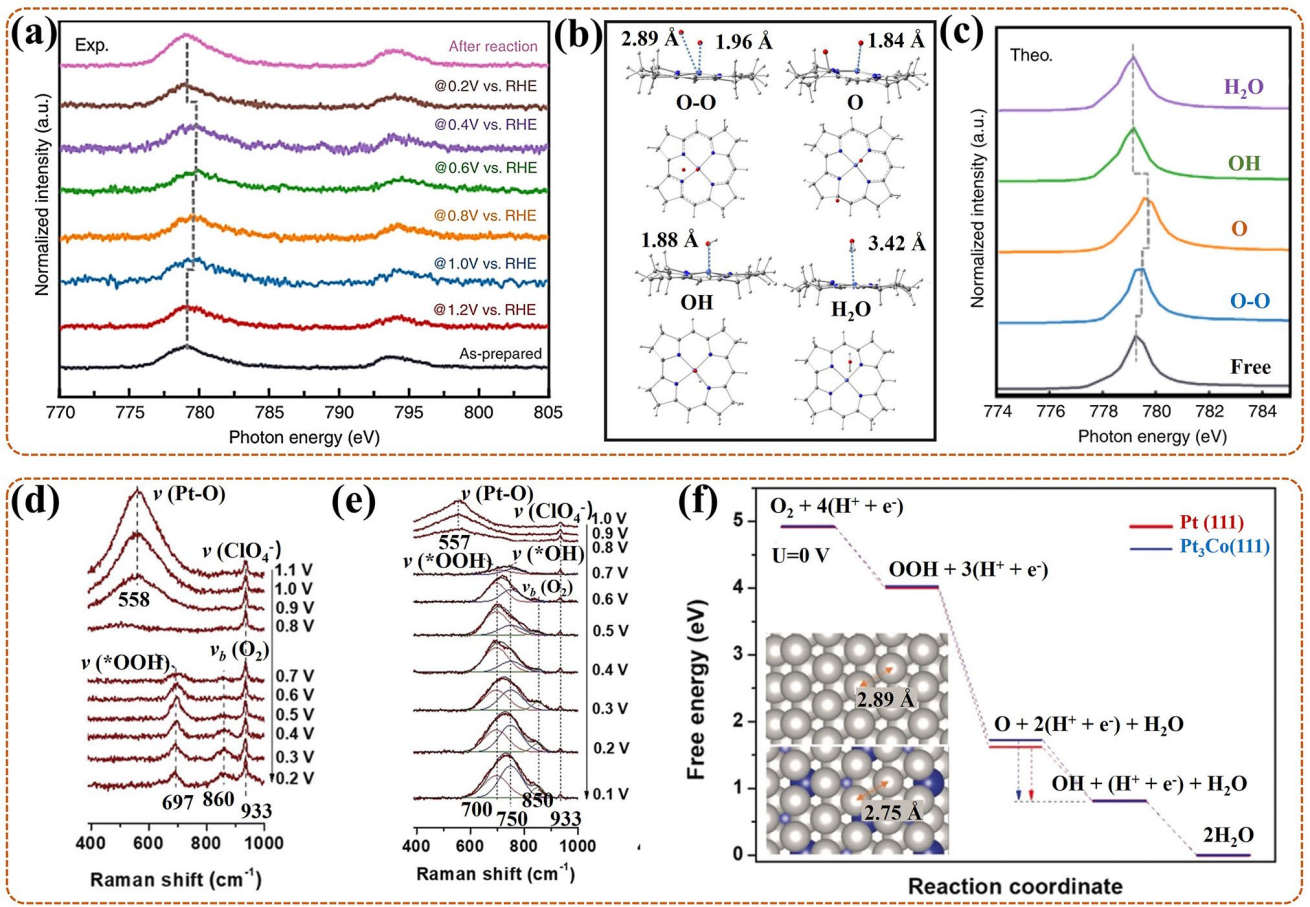
**a** In-situ Co L_3,2_-edge XANES spectrum of py-B12/C catalyst at different potentials; **b** Optimized structures for OO, O, OH, and H_2_O models of four-electron pathway; **c** FEFF8 calculated Co L_3,2_-edge XANES spectrum with different oxygenated intermediates [145]; Reused with approval; Copyright 2020 Nature. In-situ EC-SHINERS spectra of Pt_3_Co during ORR in O_2_ saturated in **d** 0.1 M HClO_4_, or **e** 0.1 M NaClO_4_ + 1 mM NaOH; **f** At *U* = 0 V, the Gibbs Free Energy plots of each ORR steps on the Pt_3_Co (111) (blue) and Pt (111) (red), with their atomic models in the left bottom inset [140]. Reused with approval; Copyright 2019 WILEY-VCH Verbg GmbH&Co. KGaA, Weinheim. (Color figure online)

Furthermore, the FEFF8 calculation can also help to identify the real active site of the catalyst, such as for the M–N–C, especially the derived SAC, still with an elusive active site, it is very crucial to apply FEFF8 in in-situ experimental analysis. In the work of Lien et al. [145], to identify the bonding site of oxygen/ derived species on the pyrolyzed Vitamin B12 (py-B12/C, i.e., Co–Nx/C including biomimetic ligands) during ORR in fuel cell, they used in-situ XAS and FEFF8 to analysis the experimental speculations and results. As shown in in-situ Co L_3,2_-edge XANES (Fig. 14a), there were no changes for the Co^2+^ peak in the precatalytic process (at 1.2 V) compared to the original sample; at 1.0–0.4 V; this peak occurred in a positive shift; at 0.2 V, this peak returned to its initial position. These were due to that at first the O_2_ adsorbed on py-B12/C only by diffusion control. Then a chemical reaction occurred with an electron transfer from the Co 3*d* orbital to O_2_*. Finally, the product desorbed from the Co site, making it available again. Thus, these meant that the metal Co, as the active center, was partially oxidized during ORR, more importantly, the changes of Co^2+^ peak (at 1.0–0.2 V) were thought to be attributed to the sum of several Co^2+^-oxo intermediates. In detail (Fig. 14b), the O-O spontaneously dissociated after O_2_ adsorption and then occurred chemisorption on Co site, forming a 1.96 Å Co–O bond, boosting the O–O separation. The separated O bonded with Co by a 1.84 Å Co–O bond, then reacted with H to form HO-Co, further producing two H_2_O. Based on FEFF8 calculations (Fig. 14c), the bond of O–O and O with Co trigged Co oxidation state changes, reflecting a positive Co peak shift, with the latter contributing more, while the products of OH and H_2_O did not trigger any changes, as reflected in the lack of peak shift. It identified that the active site was metal Co rather than Co–N, also, Co–O bond could be used as a complement to Co–N bond, thus improving the activity of Co–Nx/C-type catalysts. This work brings us guidance to combine FEFF8 with in-situ XAS to identify the actual active site, especially, regarding the interaction of O_2_ with relevant intermediates.

Density functional theory (DFT) calculation, as a powerful auxiliary tool to reveal the ORR mechanism, has been widely used to predict the adsorption energy of reaction intermediate, and provide the information on activation energy and electronic structure of catalyst [58]. Based on the DFT results, a large amount of works have been done to explain experimental phenomena at the atomic scale. To make clear the possible ORR mechanism for Pt_3_Co NP catalyst during reaction in fuel cell, Wang et al. [140] delved the association mechanism of *OOH on its surface via in-situ SHINERS combined with DFT. In 0.1 M HClO_4_, in-situ EC-SHINERS spectrum showed that (Fig. 14d), only the 933 cm^−1^ peak (ClO_4_^−^) remained constant, while, the 558 cm^−1^ peak (Pt–O) occurred in decrease with decreasing potential and disappeared at 0.8 V, and the 697 and 860 cm^−1^ peaks started to emerge at 0.7 V. Combining the DFT, the 711 and 876 cm^−1^ peaks were attributed to the O–O extending of *OOH and O_2_*, also both with a bridge site on the Pt surface, responding to the 697 and 860 cm^−1^ peaks in the experiment. In 0.1 M NaClO_4_ + 0.1 mM NaOH (Fig. 14e), the 557 cm^−1^ peak (Pt–O) was only visible at high potential, and a broad peak appeared at 0.7 V then split into the 698 cm^−1^ (*OOH) and 750 cm^−1^ peaks with decreasing potential. Combining the DFT, the 750 cm^−1^ peak was related to the *OH adsorbed on the Co site. Notably, the *OH signal only existed in alkaline, proving that Co had a high oxygen affinity and could co-exist with Pt on the catalyst surface in alkaline, thereby resulting in worse ORR activity, contrarily, Co would leach out in acid, so without *OH signal, and with better activity. More importantly, taking *OOH as the intermediate, the possible ORR process in acid solution was explored by DFT (Fig. 14f). Firstly, * + O_2_ + H^+^  + *e*^−^ → *OOH, secondly, *OOH + H^+^ + *e*^−^ → *O + H_2_O, thirdly, *O + H^+^  + *e*^−^ → *OH, fourthly, *OH + H^+^  + *e*^−^ → H_2_O. Thereinto, the energy deviation among *O and *OH of Pt_3_Co (blue arrow) increased by 0.11 eV than pure Pt (red arrow), supporting that the adsorption of *O on Pt_3_Co was weak, so its ORR activity was higher. Further based on DFT (left bottom inset), this phenomenon was caused by the strain effect, and the lattice constant of Pt_3_Co decreased by 2.3%. In summary, this work shows that the DFT can help to exactly identify the intermediate on the catalyst surface during ORR, further predicting the possible ORR mechanism. Notably, it supplies evidence that the DFT also can help to reveal the essence of the performance improvement from the aspect of intermediate adsorption, and lattice strain.

**Fig. 15:**
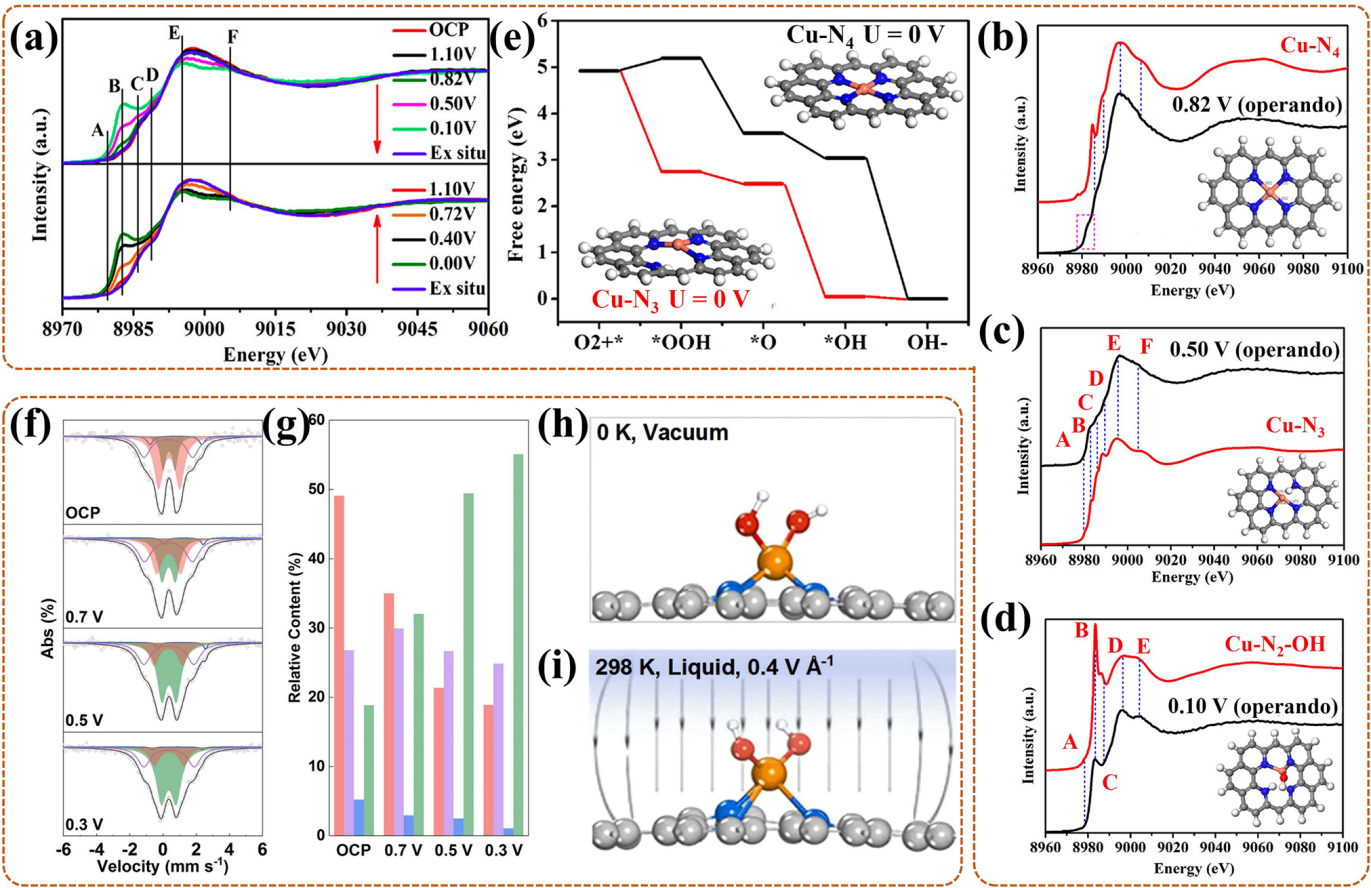
**a** In-situ XANES spectra of Cu K-edge for Cu–N–C SAC catalyst in the cathode (top) and anode (bottom) at different potentials; The selecting XANES spectra at **b** 0.82 V, **c** 0.50 V, **d** 0.10 V, with theoretical Cu–N_4_, Cu–N_3_, Cu–N_2_–OH as comparison, respectively, also with each model in each right bottom inset; **e** At *U* = 0 V, the Gibbs Free Energy plots of each ORR steps on Cu–N_4_ and Cu–N_3_ sites [138]; Reused with approval; Copyright 2021 American Chemical Society. **f** In-situ Mössbauer spectroscopy of the ^57^Fe–N–C at various potentials and **g** the matching relative content of D1, D2, D3, D4 for FeN_4_; The AIMD simulations for the average bond distance in FeN_4_C_8_ * 2OH at **h** 0 K, vacuum, **i** 298 K, electrolyte, electric field of 0.4 V Å^−1^ [143]. Reused with approval; Copyright 2022 Elsevier

The DFT calculation can also be used as the key tool for the identification of the real active site of the catalyst. Moreover, for the M–N–C-like SAC catalyst, the identification of its active site is crucial, as described in Sect. 4.4, yet the real active site remains elusive. Thus, its determination requires not only in-situ techniques to characterize but also DFT calculation to verify experimental speculations and results. To exposit the real active site of the Cu–N–C SAC during ORR in PEMFCs, Yang et al. [138] monitored the dynamic evolution of the Cu–N site by in-situ XAS, and in combination with DFT. Concretely, as shown in in-situ Cu K-edge XANES (Fig. 15a), the white-line peak (E) occurred in decline and negative shift, while the B peak belonging to Cu^+^ gradually appeared and obviously increased, under potential drive. This implied that Cu^2+^ was gradually reduced to Cu^+^, while Cu^+^ was produced at the cost of Cu^2+^, thus presumably Cu single atom shuttled between Cu^+^ and Cu^2+^ during catalyzed ORR. To clarify the dynamic changes of the active center (Fig. 15b–d), in-situ spectra at 0.82, 0.50, and 0.10 V were modeled by the finite difference method near edge structure calculation, and finally exhibited good matches with the structures of Cu–N_4_, Cu–N_3_, and Cu–N_2_–OH, respectively. This meant that the active center of the Cu–N–C catalyst changed dynamically under potential drive as follows: Cu^2+^–N_4_ → Cu^+^–N_3_ → HO–Cu^+^–N_2_. To determine the reason for Cu^+^–N_3_ with higher ORR activity (Fig. 15e), the ORR free energy changes of Cu^+^–N_3_ and Cu^2+^–N_4_ were calculated by DFT under *U*_RHE_ = 0 V, and the energy profile of the former (with a constant decline) was always lower than that of the latter (with an increase in first step) at all the process. This indicated the rational reason was that the O_2_ → *OOH proceeded spontaneously on the Cu^+^–N_3_ site, while, it needed to overcome 0.28 eV barrier on the Cu^2+^–N_4_ site, and subsequent evolution of *O and *OH intermediates were also easier to occur on the Cu^+^–N_3_ site. So, Cu^+^–N_3_ was the real active site structure for the Cu–N–C SAC. This work complements the role of DFT in that it facilitates auxiliary in-situ testing to reveal structure varies in the active site and to clarify the main active site of the catalyst during ORR.

Furthermore, to resolve the question of low metal loadings for M–N–C-like SAC, more studies focus on the creation of defective carbon (such as with vacancy and heteroatom) and dual-metal atomic sites. For the latter improvement option, the accurate identification of the master active site is relatively complex, so it is necessary to combine DFT calculations with the in-situ characterizations for the analysis. For example, to identify the master active site of the Fe/Co dual single atoms loaded porous N-doping carbon nanofibers (Fe, Co SAs-PNCF) during its catalysis reaction, Jiang et al. [146] combined DFT, and in-situ XAS, Raman to produce the analysis. In detail, the Fe and Co K-edges EXAFS spectra of the Fe, Co SAs-PNCF were fitted with a series of DFT simulated FeCo–N models, and the result in good agreement with the N_3_–Fe–Co–N_3_ model, implying that the theoretical master active site was N_3_–Fe–Co–N_3_. Then in-situ Raman analysis showed that the FeOOH and CoOOH species were observed to gradually transform to the Fe(OH)_2_ and Co(OH)_2_ species during ORR, indicating both Fe and Co species as the active sites. Further, in-situ XAS analysis showed that the Fe and Co oxidation states all occurred in reduce during ORR, conducting that there existed Fe and Co sites as the active centers. Thus, the N_3_–Fe–Co–N_3_ with Fe and Co dual sites was identified as the master active site. It ensured high metal loadings for the Fe, Co SAs-PNCF, namely, it increased the number of effective active sites, further decreasing the reaction barriers and boosting the ORR activity in metal–air/O_2_ battery and fuel cell. This work provides evidence that the DFT can help accurately identify the master active site of the catalyst in complex conditions and reveal the nature of the activity improvement.

Notably, the electrocatalytic activity of Fe–N–C derived SAC, as the higher activity M–N–C-like SAC, has been widely proved to be related to its spin state, as shown in Sect. 4.4. However, the relationship between the catalytic activity and the spin state of this SAC is still unclear, also, the reason for its poor stability remains unclear. It is essential to use the ab initio molecular dynamics (AIMD) to simulate the interaction of O_2_ with Fe–N–C under diverse conditions further helping to exposit the structural evolution during ORR. Recently, to reveal the above-mentioned relationship, and poor stability reason, Xu et al. [143] minutely analyzed the dynamic evolution of the active sites in Fe–N–C catalysts during ORR in PEMFCs via in-situ Mössbauer spectroscopy, DFT and AIMD. As shown in in-situ ^57^Fe–N–C Mössbauer spectra (Fig. 15f), there existed D1, D2, D3, and D4 four double peaks, as the potential decreased, the D1 (low-spin (LS) FeN_4_C_8_ and high-spin (HS) FeN_4_C_12_) decreased notably, and the D2 (LS or intermediate-spin (MS) FeN_4_C_10_) only occurred in decrease at higher potential then remained stable at below 0.7 V, in contrast, the D4 (FeN_4_ adsorbed with oxygen species [124, 147]) increased significantly. This meant that both D1 and D2 sites underwent demetallation during catalyzed ORR, thus resulting in the degradation of Fe–N–C catalyst, notably, the D1 with a faster demetallation rate was regarded as the main reason of catalyst degradation. Moreover (Fig. 15g), the D1 occurred more obvious transition during catalyzed ORR at lower potential, when there were more intermediates in the reaction. This implied that the D1 was the main active site and largely contributed to the higher ORR activity. Combined with DFT, the active sequence was FeN_4_C_12_ (D1) > FeN_4_C_8_ (D1) > FeN_4_C_10_ (D2), in line with that D1 had high activity. Further, the stability sequence was calculated by DFT as FeN_4_C_12_ (D1) > FeN_4_C_10_ (D2) > FeN_4_C_8_ (D1), also supporting the results: FeN_4_C_8_ (low stability, D1) suffered demetallation, and leading to the rapid degradation of Fe–N–C in early stage; while FeN_4_C_12_ (high activity and stability, D1) persisted existence, and deciding the subsequent high activity of Fe–N–C. In-depth studies showed that Fe–N–C would decay rapidly only in the occurrence of both more intermediates and potentials. This might be due to that the Fe–N bond strength decreased sharply after extensive intermediate adsorption also under the electric field condition, further aggravating the demetallation. According to the real ORR state simulated by AIMD, for FeN_4_C_8_, its Fe–N bond length was 1.841 Å under ideal condition (Fig. 15h), while the length reached 2.730 Å after intermediate adsorption and under an electric field of 0.4 V Å^−1^ (Fig. 15i). The length of Fe–N bond for FeN_4_C_8_ was only almost 20% of that in the ideal state, so it was prone to breakage and demetallation, in line with the poor stability result, also verifying the speculation of the reason. This work supports that AIMD is a powerful method to simulate real operating conditions for showing structure varies of the active site further revealing the real reason of poor stability. Also, it proved that it is necessary to combine DFT with AIMD to assist in-situ analysis.

For transition metal-based catalysts, except for the above discussed M–N–C-like catalysts, there exists some valence states complex catalysts discussed in Sect. 4.2, a combination of calculation and in-situ characterization is also necessary to reveal the essence of enhanced activity. Such as, for Li/Na/K/Rb/Cs-MnO_X_ catalysts with complex valence states, the affection of inserted alkaline cations (Li/Na/K/Rb/Cs) among adjacent layers of multilayer MnO_X_ towards ORR was explored by Kosasang et al. [148] via coupling DFT with in-situ XAS. The results showed the ORR activities were as follows: first, Li-MnOx, then, Na-MnOx and K-MnOx, finally, Rb-MnOx and Cs-MnOx. Based on DFT, the Gibbs free energy plots of each ORR steps on these catalysts showed that the free energy variation of the first step (with adsorbed *OH) was the largest, which was considered as the decisive step of the reaction. Notably, Li-MnO_x_ exhibited the smallest first free energy; thus, it possessed the highest catalytic activity for the ORR. Further, the excellent catalyzed process should accompany by the decrease of Mn oxidation state, similar as the work of Yang et al. [84]. In this work, in-situ XANES of Mn K-edge of Li-MnO_x_ revealed the valence states of Mn did occurred in change from + 3.28 (among Mn_2_^III^O_3_ and Mn^IV^O_2_) to + 2.85 (among Mn_3_^II,III,III^O_4_ and Mn_2_^III^O_3_). Thus, the DFT and in-situ XAS together proved that inserted Li cation induced the phase transition (reflected as valence changes) in Li-MnO_x_, further resulting in the highest catalytic activity.

Based on the above works, it can show that the substantial relevant information, such as d-band center, electron transfer, adsorption energy, and Gibbs free energy, can be obtained through the above theoretical calculations. Further, these can help to elucidate the relationship of structural information (such as atomic arrangement and chemical composition) and the performance for the catalyst, also help to identify the real catalytic site/center, thereby guiding the synthesis of a novel catalyst with wide application (not only in fuel cell but also in metal–air/O_2_ batteries). Moreover, these can help reveal the adsorption conformations of intermediates, further clarifying the ORR mechanism. However, there exists some inherent drawbacks in these calculations. In detail, FEFF8, as a dedicated calculation for XAS, is not suitable for other in-situ techniques. For DFT [149–151], it is always carried out in vacuum, which differs from the real complex reaction conditions, resulting in some discrepancies among experimental and calculated results. Also, the large number of approximations and the limited number of calculated atoms make the DFT results somewhat inaccurate and lacking in generality. For AIMD [152, 153], the large computational workload makes it unsuitable for complex systems. In addition, the established descriptors are scanty and with strong specificity, which further limits the use of these calculations. The d-band center can only be used for transition metal-based catalysts, but not for main group element series. In conclusion, considering that these calculations have some inherent drawbacks, it is not appropriate to use them directly, but it is better to use them as an aid to in-situ characterization during ORR. Also, it is necessary to develop more new descriptors for monitoring the catalyst activities of multifarious catalysts based on the theoretical calculation.

### Designing In-Situ Test System and Coupling Multiple Techniques for ORR Detections

Although great progress has been made in the use of in-situ techniques for structural monitoring of representative catalysts and for the detection of the evolutionary state of species on their surfaces, there are many challenges to the widespread application of each technique (Table 1). As shown in Sect. 2, each in-situ technology has its own specific self-made cell for testing; additionally, even when in-situ hard X-ray with good penetration is used to test ORR, the reaction solution, air bubble, and cell component all also will cause interference for the signal. Thus, a rational coupled in-situ characterization technique with other variables, such as in-situ cell, or other ex-situ technique, is critical when performing in-situ testing during ORR and beneficial for collecting high-quality data to elucidate the ORR mechanism. And the corresponding progress is further reviewed in this part. Moreover, the abbreviations in the following works, including WE, CE and RE, mean working electrode, counter electrode, and reference electrode, respectively, that will not be repeated later.

**Fig. 16:**
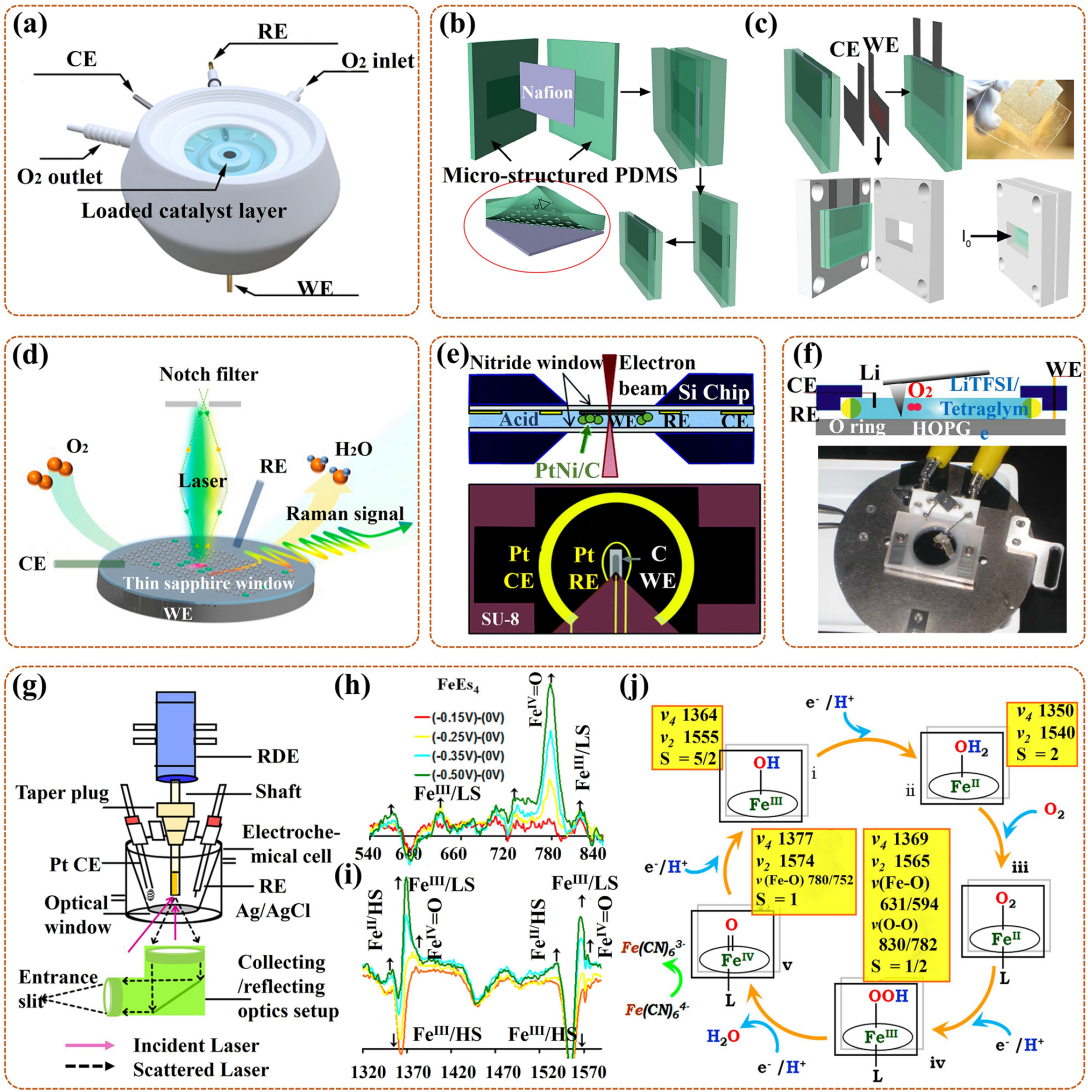
**a** Schematic of in-situ open XRD cell; [154] Reused with approval; Copyright 2020 Elsevier. **b** Fabrication schematic of the PDMS pouch, with enlarged part of the morphology for micro-structured PDMS; **c** Assembly schematic of in-situ XAS cell, with optical photo of the PDMS pouch [155]; Reused with approval; Copyright 2012 American Chemical Society. **d** Schematic of in-situ modified Raman cell [141]. Reused with approval; Copyright 2022 American Chemical Society. **e** Schematic of in-situ TEM liquid electrochemical chip, with a cross-sectional view of the holder for the cell on the top and the three electrodes view on the bottom [156]; Reused with approval; Copyright 2019 Royal Society of Chemistry. **f** Schematic of in-situ AFM cell on the top, with its optical photo on the bottom [157]; Reused with approval; Copyright 2012 American Chemical Society. **g** Schematic of in-situ SERRS with the RDE electrochemical cell; In-situ difference spectra of the SERRS-RDE data for Iron Porphyrin (FeEs_4_) **h** in the low-frequency extent, and **i** in the high-frequency extent; **j** Schematic of probable ORR mechanism [139]. Reused with approval; Copyright 2016 American Chemical Society

To guide the rational design of in-situ cell, its core skills and modification methods are summarized. For hard X-ray tests, such as in-situ XRD test, to clarify the relationship among intermediates coverage and catalytic activity of Pt-based catalyst during ORR in Zn–air battery, Ji et al. [154] designed an open cell to reflect information in terms of Pt (111) peak intensity changes not phase changes. In detail, the open cell resembled an upside-down three-electrode electrochemical testing system, and the WE with catalyst layer was right in the center for facilitating ray detection (Fig. 16a). Moreover, the design of the recessed chamber not only ensured that the electrolyte did not overflow, but also avoided the influence of the window on the ray signal. Subsequently, in-situ XRD observation coupling with open cell showed that there existed significant (111) peak intensity changes, due to continuous intermediates coverage, and corresponding to the higher ORR activity. This meant that the rich oxygen adsorption resulted in higher activity for the Pt-based catalyst, and the change in peak intensity could act as a bridge between them. In conclusion, this work offers a new idea to design a cell with a special open chamber. The open cell design helps to ensure that in-situ XRD observation of peak intensity can serve as a descriptor of catalytic activity for catalyst, thus guiding in-situ analysis from multiple perspectives, not only in phase changes. Extensively, the use of open cell is beneficial to assist in-situ XRD to reveal ORR mechanism.

Also, for in-situ XAS analysis, the typical cell design has been reported by Erickson et al. [155] in the early stages. To ensure in-situ XAS test during ORR of fuel cell under high working current density, they developed an in-situ electrochemical cell with a thin polydimethylsiloxane (PDMS) window and a high oxygen flux WE. Concretely, the core PDMS pouch was prepared as followed (Fig. 16b): two micro-structured PDMS were thermally pressed on both sides of the Nafion membrane to form a PDMS membrane, accompany with a sacrificial bulking layer forming, and was peeled off subsequently, finally, the pouch was made by cutting away the top of this PDMS membrane. Notably, the microstructure of PDMS, with a pillared arcade, ensured the proper distance among PDMS and Nafion membrane (Fig. 16b, enlarged part). The assembly of in-situ cell was as followed (Fig. 16c); the WE and CE were separated to both chambers of the PDMS pouch. Then, two Teflon tubes were connected to each chamber to supply electrolyte. Next, a syringe without the plunger, as the RE reservoir, was connected to electrolyte by one tube. Finally, the cell was fixed by Teflon compression plate also with a transmissive window. Notably, the PDMS pouch was much thin, being able to ensure the ample oxygen flux to WE (Fig. 16c, optical photo). In-situ XAS testing of Pt-catalyzed ORR showed that this in-situ cell ensured accurate monitoring of the reaction, and proved that the electronic structure evolution of the metal (Pt) cluster was in dependent on potential and current. Thus, this work offers a serious reference for the prepared of the core part and assemble of the cell. It also proves that the rational design of in-situ cell can ensure that in-situ monitoring of the electronic structure of the catalyst surface is performed under operational conditions. It can ensure more accurate results of the actual changes in the ORR process and facilitate the revelation of the ORR mechanism.

For surface testing techniques, such as in-situ Raman and FT-IR, considering that they can only capture the atomic layer signals on the catalyst surface and cannot detect the deeper signals, it is more necessary to design reasonable in-situ cells to assist the testing. The following takes Raman analysis as an example. To probe changes in the structure of the active site of a typical Fe–N–C catalyst, and reveal the ORR mechanism, Wei et al. [141] coupled a self-modified Raman cell with in-situ Raman test to obtain a more accurate monitoring of intermediates for reverse revelation. In detail (Fig. 16d), a three-electrode (including WE, CE, and RE) epoxy pool cell, assembled with a thin sapphire window (0.5 mm), and linked with the potentiostat, was as in-situ modified cell. Notably, the distance among thin window and WE surface was controlled to within 0.1 mm, ensuring that the solution layer between them was as thin as possible, thus ensuring that the weakening effect of thin layer on the Raman signal was minimized. Moreover, a notch filter (532 nm) was added in front of the receiver to cancel the laser beam as the backward scattered light returned. Subsequent in-situ Raman tests in the modified cell during ORR in PEMFC showed that for Fe–N–C catalyst, expect for Fe–Nx site, two C–N sites (graphitic/pyridinic N) were identified as independent active sites, and with *O^2−^ and *OOH as adsorption intermediates, respectively. Thus, this work provides guidance for the improvement of the Raman cell by focusing not only on in-situ cell itself, but also on the filtering of the laser. It also proves that the design of a rational in-situ Raman cell can make the decoupling of the multiple coexisting active sites become possible and help ensure the accurate identification of intermediates to confirm the ORR mechanism.

For catalyst morphology evolution, two in-situ techniques occupy the dominant position. One is in-situ TEM testing, more studies focus on the real-time monitoring of in-situ heating synthesis process (like discussed in Sect. 4.3) to guide the synthesis of high activity ORR catalyst, and the design of in-situ heating cell is more mature (such as in-situ heating holder). This does not mean that in-situ monitoring during electrochemistry process is not important, contrarily, this monitoring is essential to reveal the relationship between the structural stability of the catalyst and elucidate the ORR mechanism. Beermann et al. [156] coupled an in-situ TEM with in-situ electrochemical liquid cell to monitor varies (i.e., degradation pathways) that occurred on the carbon-supported octahedral Pt–Ni alloy nanoparticle (Pt–Ni/C NP) catalyst during ORR in PEMFC. Specifically, this in-situ cell was a flow cell chip based Proto-chips Poseidon holder, and the chip integrated WE, CE, RE and electrolyte to ensure operating conditions, also with a silicon nitride window and a thin liquid layer to ensure imaging (Fig. 16e). Such cell could help track the changes occurred on the Pt-Ni/C NP surface in a few seconds under standard or extreme cycling, further accurately relating the respond of microstructure to the applied potential. The test results showed that there was no dissolution of the Ni in the octahedral Pt-Ni alloy under cycling up to 1.0 V, even holding stable at 1.2 V, eventually promptly coarsening at 1.4 V. Additionally, carbon corrosion accelerated the migration and agglomeration of Pt–Ni NP along the octahedral (111) crystal plane. Based on their observations in this in-situ cell, it was necessary to construct stable carbon carrier and alloy to solve the catalyst degradation problem, also, the control of reaction conditions, such as potential, was also important. Thus, it is essential to design a reasonable in-situ electrochemistry cell to ensure the real-time monitoring of the catalyst degradation mechanism, thereby guiding its structure design.

Another in-situ imaging test, i.e., AFM, for metal–air/O_2_ battery to monitor the phase evolution of intermediates/products, is only force-based and requires relatively simple cell to assist test. To observe the reaction details of the ORR process on the air electrode (with a highly oriented pyrolytic graphite, i.e., HOPG, as the catalyst), Wen et al. [157] designed a simple cell to aid in-situ AFM test during ORR in Li-O_2_ battery. Precisely (Fig. 16f), the cell contained HOPG as WE, Li wire as both CE and RE, also with O_2_-saturated organic electrolyte. Notably, the HOPG as the catalyst underwent a series of cleaning treatments, such as heat-treated, up-layer stripping, to provide conditions for better phase evolution detecting. In addition, the Li wire was suspended along the Pt wire and the inner cell wall to shun contact with the insulated AFM tip (made of triangulated silicon nitride). Based on this self-made cell, the results offered by in-situ AFM showed that the formed nanoparticles were observed to grow quickly at the HOPG step edge, and formed nanoplates then grown continually to large size, and finally grown to form a Li_2_O_2_ film, during ORR. Thus, this work proves that the couple of in-situ AFM and modified cell can ensure to provide the visual evidence of the direct observation for the details of intermediates/products evolution. This actual obtained information can be used to clarify the ORR mechanism. It also offers a guidance that keeping the distance of the electrode and AFM tip is the key skill for the design of in-situ AFM cell.

It is worth noting that rotating disk electrode (RDE), as a typical method to reduce mass transfer effect, is often used in the study of ORR which is greatly affected by mass transfer. Thus, in addition to the critical importance of various in-situ cell development, the concatenation of RDE is also important for in-situ characterization of ORR. For example, Sengupta et al. [139] used in-situ SERRS coupled with RDE (Fig. 16g) to identify intermediates and investigate the ORR mechanism with iron porphyrin as the catalyst. Taking iron α 4-tetra-2-(4-carboxymethyl-1,2,3-triazolyl)-phenyl porphyrin (FeEs_4_) as an example, the differential spectrum of FeEs_4_ indicated that, in the low-frequency region (Fig. 16h), there were observed with several peaks; thereinto, the 830, 782, and 631 cm^−1^ peaks all with notable increased intensities as the potential decreasing. After the experiments in buffers of ^16^O_2_ and ^18^O_2_, it confirmed that the 830 and 631 cm^−1^ peaks represented the of O–O and Fe–O vibrations in the LS Fe^III^–OOH, the 782 cm^−1^ peak represented the Fe–O stretching in the Fe^IV^=O. More, in the high frequency region (Fig. 16i), there were observed with two class peaks, i.e., *ν*_*4*_ and *ν*_*2*_ vibrations, and the HS Fe^II^–OH_2_ (1352 and 1540 cm^−1^), LS Fe^III^–OOH (1369 and 1565 cm^−1^), and Fe^IV^=O (1371 and 1571 cm^−1^) all with increased intensities, while HS Fe^III^–OH (1364 and 1555 cm^−1^) with decreased intensity, as the potential decreasing. The above in-situ SERRS-RDE results revealed that, from kinetic control to mass transfer control region, HS Fe^II^–OH_2_, LS Fe^III^–OOH, and Fe^IV^=O all gradually accumulated at the electrode, with at the cost of HS Fe^III^–OH. Therefore, in the kinetic region ensured by RDE, the ORR mechanism and the conversion of Fe was as follows (Fig. 16j): as HS Fe^III^–OH largely reduced to HS Fe^II^–OH_2_, the O_2_ derivative species rapidly decreased, (i → ii, rate-determining step), then, the further combination with O_2_ and gradually with H^+^/*e*^−^ all occurred slowly, proving by the slow decay of HS Fe^II^–OH_2_ (ii → iii), LS Fe^III^–OOH (iv → v) and Fe^IV^=O (v → i). The application of two combined techniques ensures the direct recognition of the dynamic changes of O_2_ derivative species on the surface of iron porphyrin-like electrode during ORR. It also provides a new idea for the combined application of in-situ characterization technique and electrochemical testing technique.

Rotating ring disc electrode (RRDE), with an additional Pt ring electrode than RDE, is also always used in the research of ORR. Recently, Ni et al. [142] also coupled a RRDE with in-situ Mössbauer spectroscopy to reveal the relationship of the active sites and the ORR pathway in PEMFC, with porphyrin-based FeNC as the catalyst. Thereinto, the Pt ring was mainly used for detecting the formation of H_2_O_2_. The results showed that, for the D3 (N–FeN_4_C_10_) active site, it appeared at 0.8 V, and mainly contributed to the direct oxygen reduction, i.e., four-electron pathway, with only little contribution to the formation of H_2_O_2_. For the D2 (FeN_4_C_10_) site, it emerged at 0.6 V or lower potential, and mainly devoted to the indirect oxygen reduction, i.e., peroxide reduction reaction (PRR). Thus, the “start potential” of the two Fe active site were different, and were related to different catalytic pathways, in which the pathway was indirectly revealed by the identification of the H_2_O_2_ through RRDE. This further strengthens our awareness of combining in-situ characterization with electrochemical testing. The coupling strategies help to ensure any in-situ technique to reveal the actual ORR process, thereby boosting the clarification of the ORR mechanism.

This part further confirms that to accurately monitor any changes in real time during ORR, the construction of in-situ test system is essential. Notably, in liquid solutions, more optical spectra can be used to reflex the signals from the molecular level, while, there still exists difficulties for electron microscopy to visualize the changes (such as migration and agglomeration) at the atomic level. Thus, it is still urgent to explore and develop novel in-situ cell for the electron microscopy. In addition, more coupling techniques, except for the above mentions, should receive attention and the technical difficulty that needs to be broken is the modification of the laser beam channel.

In conclusion, the application of in-situ optical techniques (IR, Raman), electron technique (TEM), and scanning probe technique (AFM) for probing the evolution states of intermediates and products are summarized (Fig. 1). These are helpful to clarify the ORR mechanism, and can help to indirectly reveal the active site of catalyst for guiding the synthesis of the catalyst. In addition, in-situ IR/Raman monitoring of the anion chemisorption and in-situ ETS for halides are conducive to provide a realistic in-situ detection of external species on the catalyst surface under actual operating conditions. These external species tend to occupy the active sites of catalysts affecting their activity; thus, their accurate detection is beneficial to guide the structural design of anti-toxic catalysts. The combination of theoretical calculations to assign the detected in-situ signals, and associating hardware development to ensure the accuracy of experimental detection, are also proved to be non-negligible.

## Conclusions and Perspectives

**Fig. 17:**
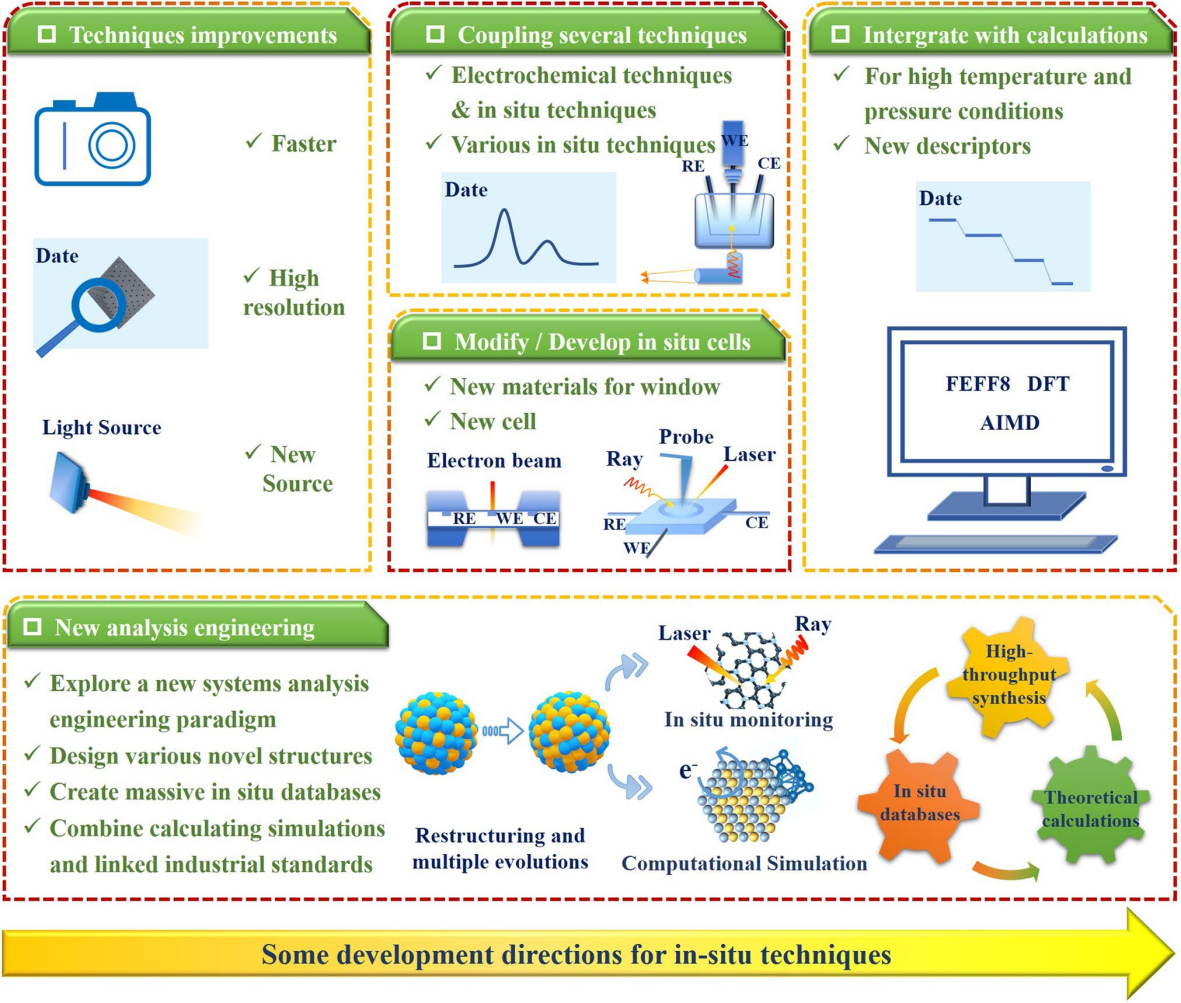
Summary of some development directions for in-situ techniques on questing ORR processes

This paper furnishes an overview of the applications of various in-situ characterization techniques in probing active sites of catalysts and revealing ORR mechanisms. In detail, the direct detection progress of catalyst structure evolution is outlined; the adsorption/desorption behaviors of intermediates/solvent anions, the formation and evolution of products are summarized; how to combine theoretical calculations to assist in assigning in-situ signals is discussed; other factors affecting the accuracy of the characterization results are also summarized, such as the designing of in-situ cells and the coupling of various techniques. The conclusion, focusing on Pt-based, M–N–C and some oxide catalysts, can be categorized into two points (Fig. 5). For one thing, the phase, valence, electronic transfer, coordination, and spin states varies of the catalysts during ORR can be directly characterized by in-situ XRD, HRTEM, XAS, SECM, and Mössbauer spectroscopy. These can help to identify the active site of the catalyst, and clarify the factors that enhance catalyst activity to guide how to design optimal catalyst structure. The extensive in-situ works have further demonstrated that catalysts synthesized with specific morphologies, high index crystal planes, vacancies, etc. tend to exhibit high oxygen reduction activity. Prominently, the use of in-situ TEM to monitor the morphological changes in the catalyst synthesis process is beneficial in finding the right temperature time at source for the synthesis of highly active catalysts. For another, in-situ detecting of intermediates using in-situ FT-IR, Raman, ETS, and in-situ detecting of products using in-situ XRD, TEM, and AFM can also indirectly achieve the above purpose. The determination of evolution states of intermediates and products facilitates the clarification of reaction pathways and reveals reaction mechanisms. Notably, the catalysts with multi-dimensional morphologies can increase the active sites utilization and promote the adsorption and desorption of intermediates and products on their surfaces, facilitating a more complete conversion of O_2_ along the 4*e* pathway. These results point to the fact that each technique has its own unique detection characteristics and should be used according to different needs. In addition, there are still many challenges and opportunities for future research in ORR in-situ characterization. There are several recommendations for the development of in-situ research on questing ORR processes as follows (Fig. 17):

1. Further improvement of in-situ observation techniques to directly observe the active site evolutions under dynamic changes.

Massive studies have illustrated that the structure of the catalyst is always reconfigured and the atomic coordination environment is altered during ORR, especially under real or high potential conditions, which further induce the dynamic evolution of the active site. To identify the active site, it is necessary to monitor the catalyst structure evolution by in-situ techniques. However, both the beam damage and the requirement of sample transparency limit the application of electron beam or low-energy photon techniques in the ORR process. High-speed and high-resolution diffraction tests should be developed and used to track the rapid reactions, and then fine structural parameters extracted from the obtained data can directly demonstrate the evolution of the active site during ORR. In addition, it will be a relative long time for the activation and deactivation of catalysts. Currently, the dedicated beamlines of some techniques (such as synchrotron radiation technique) are not applicable, so it is particularly important to find alternatives.

2. Further monitoring or visualizing variations of reaction intermediates coupled with multinomial techniques or modified cells.

The real-time monitoring of the ORR process can be crucial to approval the intuitionistic observation of the transient transformation of reaction intermediate configurations further to illustrate the catalytic mechanism. Unfortunately, the coinstantaneous monitoring of all variations of the intermediates during ORR remains a challenge due to the connatural localizations of the single test technique. Thus, the exploitation of devices integrated with in-situ techniques (such as in-situ SERRS coupled with RDE) will undoubtedly play an assignment in better understanding the ORR process. And the coupling of various in-situ techniques is also important to offer more information about the intermediate structure. Furthermore, for in-situ technology using an electron beam, it is not often acceptable to use a reaction cell containing large amounts of solution, because the solvent medium will have a strong absorption effect on the transmitted/scattered X-rays. The researcher should design the reaction cells based on the characteristics of in-situ X-rays techniques to ensure the veracity of experimental data while saving beam time. In particular, the reasonable design of reaction cells for in-situ TEM can ensure the visualizing of dynamic changes of the intermediates formed at the active sites during ORR under more realistic conditions in real time.

3. Further theoretical analysis by the integration of the insight from theory into the various in-situ techniques.

Incorporating in-situ monitoring process with the theoretical calculations is an efficient strategy for identifying active sites and uncovering ORR mechanisms. It is an inevitable trend to develop a variety of computational models to predict the structural evolution of catalysts during ORR. Especially, under extreme conditions (such as high temperature and pressure), it is difficult to realize in-situ characterization of catalyst structural evolution using experimental techniques. Meanwhile, the construction of a computational model with optimized reaction parameters to simulate the entire ORR process appears particularly important. It can help to predict the related structural evolution of the catalyst and infer ORR mechanism from theoretical analysis. Moreover, it is equally significant to continue model optimization to ensure the integration of theoretical calculations with different experimental techniques. It will simplify the process of mechanism research and allows one to rapidly construct a highly efficient catalyst.

4. Explore a new systems analysis engineering paradigm to guide the design of catalysts to advance their industrial ORR applications.

Many novel structures have been devised and utilized to regulate the intrinsic activity of Pt-based, M–N–C, oxide catalysts, yet the true structures of their active moieties and the structure-effect relationships remain fuzziness. Exploring a new systems analysis engineering paradigm that combines high-throughput synthesis with theoretical calculations and in-situ databases is central to elucidating their ORR catalytic processes. As a proof of concept, the promising single particle catalyst, with enhanced activity through the modification of each unique facets and their interfaces [158], always occurs dynamically restructuring and multiple evolutions during ORR, thus urgently requiring in-situ techniques to monitor them in real time. More mature studies on spectroscopic testing can reflect the average structural information of each single particle, but individual facets are still difficult to discern limited by resolution. Recent developments of in-situ field electron microscopy (FEM) technique [159] is expected to be implemented on single particle level characterization to enrich the databases. With the integration of various in-situ technologies, the massive databases can be created to accelerate the search speed of appropriate single particle active structures for industrial ORR applications. Further combing with theoretical calculations can provide more insight into the precise electronic environment information on the minimum catalytic block of the single particle catalyst from atomic levels. Finally, the calculating simulations should be associated with industrial standards to assist in the devise of superior ORR catalysts from the source to meet the actual industrialization.
